# Age of onset of obsessive-compulsive disorder differentially affects white matter microstructure

**DOI:** 10.1038/s41380-023-02390-8

**Published:** 2024-01-16

**Authors:** Chris Vriend, Niels T. de Joode, Petra J. W. Pouwels, Feng Liu, Maria C. G. Otaduy, Bruno Pastorello, Frances C. Robertson, Jonathan Ipser, Seonjoo Lee, Dianne M. Hezel, Page E. van Meter, Marcelo C. Batistuzzo, Marcelo Q. Hoexter, Karthik Sheshachala, Janardhanan C. Narayanaswamy, Ganesan Venkatasubramanian, Christine Lochner, Euripedes C. Miguel, Y. C. Janardhan Reddy, Roseli G. Shavitt, Dan J. Stein, Melanie Wall, Helen Blair Simpson, Odile A. van den Heuvel

**Affiliations:** 1 Amsterdam UMC, Vrije Universiteit Amsterdam, Department of Psychiatry, and Department of Anatomy and Neuroscience, de Boelelaan 1117, Amsterdam, the Netherlands; 2 Compulsivity, Impulsivity and Attention, Amsterdam Neuroscience, de Boelelaan 1117, Amsterdam, the Netherlands; 3 Amsterdam UMC, Vrije Universiteit Amsterdam, Department of Radiology and Nuclear Medicine, Amsterdam Neuroscience, de Boelelaan 1117, Amsterdam, the Netherlands; 4 Brain Imaging, Amsterdam Neuroscience, de Boelelaan 1117, Amsterdam, the Netherlands; 5 Columbia University Irving Medical Center, Columbia University, New York, NY 10032 USA; 6 The New York State Psychiatric Institute, New York, NY 10032 USA; 7 LIM44, Hospital das Clinicas HCFMUSP, Instituto e Departamento de Radiologia da Faculdade de Medicina, Universidade de São Paulo, São Paulo, SP Brazil; 8 Cape Universities Body Imaging Centre, University of Cape Town, Cape Town, South Africa; 9 SAMRC Unit on Risk & Resilience in Mental Disorders, Department of Psychiatry & Neuroscience Institute, University of Cape Town, Cape Town, South Africa; 10 Obsessive-Compulsive Spectrum Disorders Program, LIM23, Hospital das Clinicas HCFMUSP, Instituto & Departamento de Psiquiatria da Faculdade de Medicina, Universidade de São Paulo, São Paulo, SP Brazil; 11 Department of Methods and Techniques in Psychology, Pontifical Catholic University, Sao Paulo, SP Brazil; 12 National Institute of Mental Health & Neurosciences (NIMHANS), Bangalore, India; 13 SAMRC Unit on Risk & Resilience in Mental Disorders, Department of Psychiatry, Stellenbosch University, Stellenbosch, South Africa

**Keywords:** Psychiatric disorders, Neuroscience

## Abstract

Previous diffusion MRI studies have reported mixed findings on white matter microstructure alterations in obsessive-compulsive disorder (OCD), likely due to variation in demographic and clinical characteristics, scanning methods, and underpowered samples. The OCD global study was created across five international sites to overcome these challenges by harmonizing data collection to identify consistent brain signatures of OCD that are reproducible and generalizable. Single-shell diffusion measures (e.g., fractional anisotropy), multi-shell Neurite Orientation Dispersion and Density Imaging (NODDI) and fixel-based measures, were extracted from skeletonized white matter tracts in 260 medication-free adults with OCD and 252 healthy controls. We additionally performed structural connectome analysis. We compared cases with controls and cases with early (<18) versus late (18+) OCD onset using mixed-model and Bayesian multilevel analysis. Compared with healthy controls, adult OCD individuals showed higher fiber density in the sagittal stratum (*B[SE]* = 0.10[0.05], *P* = 0.04) and credible evidence for higher fiber density in several other tracts. When comparing early (*n* = 145) and late-onset (*n* = 114) cases, converging evidence showed lower integrity of the posterior thalamic radiation —particularly radial diffusivity (*B[SE]* = 0.28[0.12], *P* = 0.03)—and lower global efficiency of the structural connectome (*B[SE]* = 15.3[6.6], *P* = 0.03) in late-onset cases. Post-hoc analyses indicated divergent direction of effects of the two OCD groups compared to healthy controls. Age of OCD onset differentially affects the integrity of thalamo-parietal/occipital tracts and the efficiency of the structural brain network. These results lend further support for the role of the thalamus and its afferent fibers and visual attentional processes in the pathophysiology of OCD.

## Introduction

Previous diffusion-weighted imaging (dMRI) studies observed alterations in the white matter microstructure and global structural connectivity of individuals with an obsessive-compulsive disorder (OCD), a debilitating psychiatric disorder characterized by repetitive thoughts (obsessions) and behaviors (compulsions) that affects approximately 2% of the world population [1]. The largest study to date from the OCD workgroup of Enhancing Neuro Imaging Genetics Through Meta-Analysis (ENIGMA) reported lower integrity of white matter fiber bundles, such as the corpus callosum (CC), uncinate fascicle (uncF), sagittal stratum (SagS) and posterior thalamic radiation (PTR) in adults with OCD relative to healthy controls (HC) [2]. Other case-control differences were observed in the cingulum bundle [3–6], and superior longitudinal fascicle (SLF) [4, 7–10]. Previous studies also showed alterations in the global organization of the structural network in OCD compared with HC [11–13]. Together, these studies support the hypothesis that OCD is associated with alterations in brain structures beyond the cortico-striatal-thalamo-cortical (CSTC) circuits that are classically associated with OCD [1]. Nevertheless, there is considerable heterogeneity in the findings across studies, which is likely due to differences between samples in demographic and clinical characteristics (e.g., age of onset and medication history), differences in the acquisition parameters and quality of the dMRI scans, and underpowered samples. Indeed, the ENIGMA OCD meta-analysis by Piras et al. (2021) showed that only case-control differences in the SagS and PTR showed low variance between sites and were robust against leave-one-site-out cross-validation, while meta-regression showed that a younger age of onset, longer illness duration and being medicated (approximately 40% of the total sample) were all associated with lower fractional anisotropy (FA) in the SagS. This indicates that clinical and medication status explains at least some of the observed differences. Still, the lack of harmonization of clinical measures across sites and differences in acquisition parameters, prevent more fine-grained analyses.

To overcome some of these limitations, we conducted the OCD Global study that used harmonized prospective methods for clinical phenotyping, neurocognitive testing and neuroimaging to collect data from a large group of medication-free adults with OCD and HC across five international sites [14]. As described in our previous neuroimaging methods paper [15], we employed state-of-the-art neuroimaging sequences that were available across different vendors while still feasible on a clinical MRI scanner (sequences available on request). Image quality was continuously monitored by acquiring phantom scans and each human acquisition was quantitatively and qualitatively inspected for artifacts (CV, NdJ).

In this manuscript, we describe the results of our pre-registered dMRI analyses (osf.io/m97kp [16]), where we investigated case-control differences in the microstructure of specific white matter tracts and characteristics of the structural brain network and how these measures are influenced by clinical characteristics (i.e., age of onset, symptom severity, illness duration and medication history). To identify robust brain signatures of OCD that are not influenced by current medication status and are reproducible and generalizable across different countries and (diverse) cultures, we employed (1) several dMRI methods (i.e., classical tensor-based measures, Neurite Orientation Dispersion and Density imaging (NODDI), fixel-based and connectome analysis), (2) several statistical approaches (tract-wise null hypothesis significance testing (NHST) and Bayesian hypothesis testing (BHT) and whole-brain voxel/fixel analysis), and (3) two different site correction procedures (ComBat [17], and random intercept for site). We hypothesized that relative to HC, medication-free adults with OCD would show lower microstructural integrity of OCD-related white matter tracts and a less optimal topology of the structural brain network. Within the OCD group, we expected inverse associations between severity or illness duration, and tract integrity or network topology.

## Materials and Methods

### Participants

The OCD Global Study recruited medication-free (at least six weeks) adults with OCD, and age, sex and education-matched HC across five research sites in Brazil, India, the Netherlands, South Africa, and the U.S.A [14]. Participants had to be between 18-50 years. OCD had to be the primary diagnosis established using the Structured Clinical Interview for DSM-5 (SCID) with at least a Yale-Brown Obsessive-Compulsive scale (YBOCS) score ≥16. Exclusion criteria included any current psychotropics or cognitive behavior therapy use within the previous six weeks and an IQ < 80. Other exclusion criteria are detailed in the supplements. All participants provided written informed consent according to the Declaration of Helsinki and the study was approved by the five local Medical Ethical Committees. Of the 524 eligible participants (268 individuals with OCD; 256 healthy controls), dMRI data was excluded from 12 participants leaving 260 OCD cases and 252 HC for analysis (see flowchart in supplementary Fig. 1).

### Clinical measures

A full list of administered measures is provided in the supplements and in a previous study [14]. For the current study, we utilized the YBOCS as a measure for overall OCD symptom severity and the dimensional YBOCS (DY-BOCS) to rate the severity of distinct symptom dimensions of OCD. OCD participants were divided into an early-onset (<18 years) and late-onset (≥18 years) group based on the youngest age at which symptoms of OCD first interfered with activities, became time-consuming (>1 h a day) or caused significant distress. Age of onset was also used as a continuous measure (see data analysis). Duration of illness, medication history and years of education were also recorded. Interrater reliability of the clinical measures across the sites, particularly of the (D)Y-BOCS, was excellent [18].

### Image acquisition and processing

Each of the five sites acquired harmonized multi-shell dMRI, blip-up/blip-down scans with opposite phase-encoding directions to correct for susceptibility-induced distortions and 3D T1-weighted structural images. See the supplementary material for the acquisition parameters and preprocessing steps. See our previous work for details on the harmonization of the MRI protocol [15]. Raw data will be uploaded to the NIMH Data Archive. Scans were excluded in case of >3 volumes per shell with motion artifacts. Volumes (<2) had to be excluded from three subjects but these scans were kept in the analysis.

### Tensor and NODDI measures

We calculated tensor-based FA, mean diffusivity (MD), axial diffusivity (AD) and radial diffusivity (RD) maps from the *b* = 1000 s/mm^2^ shell of the preprocessed dMRI [19], and used DTI-TK to register the dMRI scans to a common space [20]. We calculated the tensor-based measures for the *b* = 1000 s/mm^2^ shell only for higher comparability with previous studies and because the diffusion tensor model does not account for non-Gaussian diffusion at higher b-values [21]. We investigated OCD-related alterations in white matter microstructure of several tracts of interest (TOI) that were chosen based on the previous ENIGMA-OCD study [2] and other previous dMRI studies showing case-control differences [3–10, 22–24]: SagS, PTR, genu, body and splenium of the CC, dorsal (along cingulate gyrus) and ventral (along parahippocampal gyrus) cingulum, SLF and uncinate fascicle derived from the JHU-ICBM-DTI-81 atlas [25]. Additionally, we used Neurite Orientation Dispersion and Density imaging (NODDI) and the NODDI-Watson model in the CUDA Diffusion Modelling Toolbox [26] to calculate neurite density (ND) and orientation dispersion (OD) maps. These maps were warped to the same DTI-TK template as the tensor maps. We extracted the median value from each TOI for further analyses. Bilateral tracts were averaged.

### Fixel measures

A fixel (i.e., fiber population within a voxel) analysis calculates fiber bundle-specific measures such as the fiber density (FD), fiber cross-section (FC) and fiber density and cross-section (FDC) [27] to overcome the problem of crossing fibers [28]. Details of the pipeline are presented in the supplementary material.

### Tractography and network analysis

We performed multi-shell anatomically-constrained (probabilistic) tractography with 50 million seeds from the gray/white matter boundary to construct a tractogram for each participant in MRtrix3 and applied SIFT2 to improve the accuracy of the reconstructed fibers and reduce false positive connections [29, 30]. The resulting tractogram was converted to a weighted structural connectivity matrix with 300 cortical areas derived from the Schaefer 300P7N atlas and 14 individually segmented subcortical areas with FreeSurfer 7.1.1. Four nodes (i.e., left/right Limbic_OFC_3 and Limbic_TempPole_1) were removed from the matrix as a high percentage of participants did not have any streamlines originating from them. This resulted in a 310 × 310 connectivity matrix per participant. We subsequently calculated network measures that describe different properties of the network organization on the global level: global efficiency, modularity, small-worldness, and rich club coefficient (see supplements for details). On the nodal level, we calculated the betweenness centrality and local efficiency for subcortical nodes that are highly implicated in the pathophysiology of OCD: amygdala, pallidum, hippocampus, thalamus and putamen.

### Statistical analysis

Analyses were pre-registered with the Open Science Framework (osf.io/m97kp). We employed two different statistical approaches: null hypothesis significance testing (NHST) and Bayesian hypothesis testing (BHT). Within the framework of NHST, we performed two sets of models for all dMRI analyses where we corrected for site using either ComBat [17], or added site as a random intercept. We performed multivariate mixed model analyses in R (v4.1.3; R CORE team, lme4 and lmerTest packages) with the four diffusivity measures and two NODDI measures in each TOI as dependent variables and diagnosis, age of onset (early vs late onset) or prior exposure to selective serotonin reuptake or serotonin-norepinephrine reuptake inhibitors (SSRI/SNRI) as independent variable. We checked for equal variance between groups. All six tensor/NODDI measures were Z-transformed, and the MD, RD and OD values were inverted to ensure that higher values signified better microstructural integrity. We added age, sex and educational level as nuisance covariates in separate adjusted models. Fixel-based and connectome measures were analyzed using univariate mixed models using the three fixel measures per TOI or global or nodal measures as dependent variables and diagnosis, age of onset or SSRI/SNRI medication history as independent variable (age, sex and educational level as nuisance covariates in separate models). Intracranial volume (ICV) was added as a covariate for the FC and FDC measures [27].

Tensor/NODDI, fixel and connectome measures were additionally linearly associated with age of onset, symptom severity (YBOCS) and duration of illness in OCD cases using mixed model analysis, with age, sex and education as covariates in adjusted models. To account for the large proportion of zero scores on sub-dimensions of the D-YBOCS (i.e. zero-inflated) [31], we added a binary regressor per sub-dimension score that indicated whether a participant scored >0 on a given sub-dimension (conform Harisson et al. [32].). Sub-dimension scores (Harm, Sexual/Religious, Symmetry/Ordening, Contamination and Collecting/Hoarding) and binary regressors were added simultaneously to the model (along with age, sex and education). For all these analyses alpha was set to *P* < 0.05 (two-sided) and we corrected for multiple comparisons across the different TOIs or nodes using the False Discovery Rate (FDR; *q* < 0.05), except for the association with D-YBOCS dimensions where we corrected for multiple comparisons using a D/AP-Sidak correction that takes into account the mutual correlation between outcome measures [33]. For the tensor/NODDI, fixel and global and nodal network analyses, the adjusted *P*-value was set at *P*_*ad*j_ = 0.0009, *P*_*adj*_ = 0.001, *P*_*adj*_ = 0.01 and *P*_*adj*_ = 0.005, respectively. We previously calculated that our sample size (at *P* < 0.05 and 80% power) is sufficient to detect effect size differences of Cohen’s d = 0.25 and correlations of size r = 0.12 [14].

BHT was applied to the ComBat corrected tensor/NODDI and fixel measures using the Region-Based Analysis Program through Bayesian Multilevel Modeling (RBA, v1.0.10) tool to consider all measures across all tracts and incorporate this shared information into one statistical model [34]. Rather than a *P*-value, BHT produces posterior distributions. The distance of the median of the posterior density from a zero-effect line represents the magnitude (and direction) of the effect, whereas the area under the curve of the distribution to the right or left of the zero line (i.e. posterior probability or *P* + ), represents the credibility (or uncertainty) of there being an effect. Although inferences should be based on the entire posterior distribution, we classified the credibility of there being an effect as moderate (*P+* between [1-]0.05 and [1-]0.10), strong (*P+* between [1-]0.01 and [1-]0.05), and very strong (*P* + < 0.01 or >0.99). Effects with a *P+* of <0.10 or >0.90 are reported in the main text.

Lastly, we performed exploratory whole-brain skeletonized voxel-wise (randomise) and fixel-wise analyses (cfestats) on the tensor/NODDI and fixel-based measures, respectively. We adjusted for age, sex and years of education (and ICV were appropriate) and limited the analyses to the voxels/fixels within the skeletonized JHU-ICBM-DTI-81 atlas and used permuted (10,000) threshold-free cluster enhancement (TFCE) and family-wise error (FWE) correction (*P* < 0.05).

## Results

### Demographics, clinical information and image quality

Demographic and clinical information is provided in Table 1. Groups were well matched on age and sex, but not years of education (*t*(510) = 3.53, P < 0.001) or estimated IQ (*t*(510) = 2.21, *P* = 0.03). The late-onset OCD group was on average older (mean age 31.2 vs 28.2 years, *t*(257) = −3.07, *P* = 0.002), had a shorter illness duration (*t*(255.9) = 8.5, *P* < 0.001) and lower estimated IQ *t*(257) = −3.18, *P* = 0.002) compared with the early onset group, but there were no differences in sex distribution, OCD symptom severity, sub-dimension scores, previous medication use or (lifetime) comorbidities (supplementary Table 2). Individuals with OCD with or without prior SSRI/SNRI history showed a significant difference in YBOCS score, prior use of other treatments and comorbidities; particularly comorbid depression, panic disorder and generalized anxiety disorder and hoarding symptoms were more prevalent in the group with exposure to SSRI/SNRI (Supplementary Table 2). Image quality was similar across all groups (Supplementary Table 3).

**Table 1:** Demographic and clinical characteristics.

	OCD (*N* = 260)	HC (*n* = 252)	Statistics
**Sex (*N*** **(%))**			
** Male**	117 (45.0%)	105 (41.7%)	χ^2^_(1)_ = 0.58, *P* = 0.45
** Female**	143 (55.0%)	147 (58.3%)	
** Age (years)**	29.6 (8.0)	30.0 (8.2)	t_(510)_ = 0.61, *P* = 0.54
** Education (years)**	15.2 (2.8)	16.0 (2.5)	t_(510)_ = 3.52, *P* < 0.001
** Estimated IQ**	104.7 (12.4)	107.1 (12.3)	t_(509)_ = 2.21, *P* = 0.03
** YBOCS**	24.8 (4.9)	0.12 (0.68)	t_(510)_ = −78.7, *P* < 0.001
** Duration of illness**^**a**^	12.3 (8.6)		
** Age of onset (years)**	17.3 (7.1)	–	
**OCD onset (*N*** **(%))**^**a**^			
Child onset	145 (55.8%)	–	
Adult onset	114 (43.8%)	–	
**Medication naïve (%)**			
SSRI	58.5%	99.2%	
SNRI	95.4%	100%	
Benzodiazepines	90.4%	99.6%	
Antipsychotics	91.9%	99.6%	
Mood stabilizers	97.3%	100%	
** CBT naïve**	75.8	100%	
**DYBOCS**			
Harm & Aggression	5.3 (4.7)	0.0 (0.4)	
Sexual & Religious	4.5 (4.9)	0.0 (0.0)	
Symmetry & Ordering	5.7 (4.4)	0.0 (0.2)	
Contamination	6.3 (5.0)	0.0 (0.2)	
Collecting & Hoarding	1.3 (2.7)	0.0 (0.0)	

Data are presented as mean (SD) unless otherwise indicated. a = duration of illness/age of onset missing for 1 individual with OCD. *YBOCS* Yale-Brown Obsessive-compulsive Scale, *SSRI* selective serotonin reuptake inhibitor, *SNRI* serotonin-noradrenaline reuptake inhibitor, *CBT* Cognitive behavioral therapy.

### Tensor and NODDI measures

**Fig. 1:**
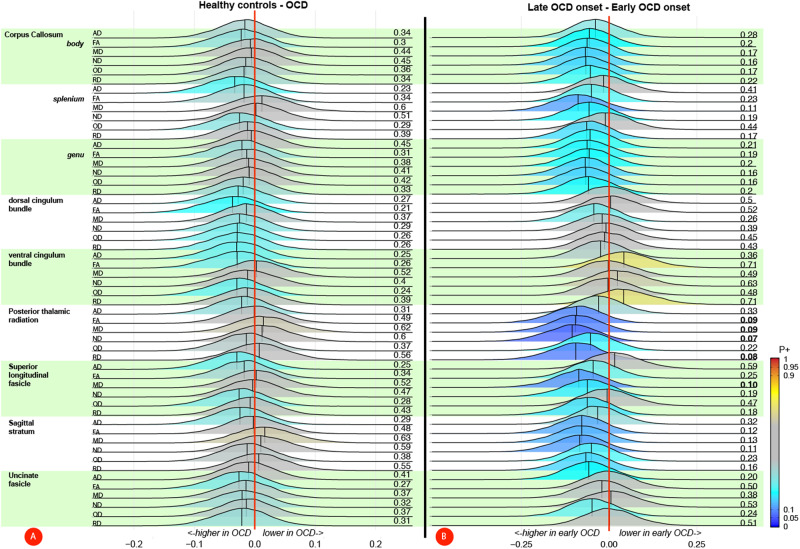
Bayesian posterior distribution plots of the differences in white matter microstructure between (a) OCD patients and healthy controls and (b) early and late onset OCD patients. The posterior distribution communicates the credibility of an effect. Posterior probabilities of a positive effect (*P* + ) are shown next to each distribution and color coded. *P+* values ≥ 0.90 (moderate to very high credibility for a positive effect) or ≤0.10 (moderate to very high credibility for a negative effect) are presented in bold. The meaning of the direction of effects are shown next to the red zero-effect line. Values on the X-axis represent (inverted) Z-scores (i.e., the unit to which the tensor/NODDI measures were converted; see methods section). **A** There was no credible evidence for differences between OCD patients and healthy controls (all 0.10 < *P* + < 0.90). **B** *P+* values were ≤ 0.10 for several tensor/NODDI measures of the posterior thalamic radiation and superior longitudinal fascicle, signifying moderate to high credibility for a higher value in early onset OCD patients. abbreviations: AD: axial diffusivity, FA: fractional anisotropy, MD mean diffusivity, ND neurite density, OD orientation dispersion, RD radial diffusivity, NODDI Neurite orientation dispersion and density imaging. Plots were produced using the Region-Based Analysis program through Bayesian Multilevel Modeling implemented in AFNI (Chen et al. 2019).

None of the tracts of interest showed a case-control difference, neither when adjusting for the site using ComBat (Table 2) nor when adjusting for a site with a random intercept (Supplementary Table 4). These results were also confirmed by the Bayesian multilevel analyses (see Fig. 1A) showing little credibility for an effect of diagnosis. When comparing cases with an early or late onset of OCD, the ComBat adjusted data showed that late-onset cases exhibit lower overall white matter microstructural integrity in the PTR (*B[SE]* = 0.20[0.08], *P* = 0.012), driven by a lower FA (*B[SE]* = 0.29[0.12], *P* = 0.02), higher RD (*B[SE]* = 0.28[0.12], *P* = 0.025), higher MD (*B[SE]* = 0.25[0.12], *P* = 0.048), and lower ND (*B[SE]* = 0.29[0.12], *P* = 0.02) and in the SLF a higher MD (*B[SE]* = 0.28[0.12], *P* = 0.03; see Table 3). These results were no longer significant after adjusting for covariates or correcting for multiple comparisons. Conversely, before adjusting for covariates we observed higher overall microstructural integrity in late-onset cases in the ventral cingulum (*B[SE]* = −0.16[0.08], *P* = 0.04), driven by a lower RD (*B[SE]* = −0.34[0.12], *P* = 0.005), which was still significant after adjusting for covariates (*B[SE]* = −0.32[0.12], *P* = 0.008). The models adjusted for site (rather than with Combat) showed very similar results for the PTR and SLF with the differences in the PTR surviving FDR correction, but conversely, no differences were observed in the ventral cingulum (Supplementary Table 5). In support of the mixed model analysis, the Bayesian multilevel analysis showed moderate (*P*+ < 0.1) credible evidence for the difference in microstructural integrity in the PTR (ND, FA, RD, MD), SLF (MD) but not ventral cingulum (see Fig. 1B). Comparing OCD individuals with and without prior use of SSRI/SNRI showed that medication-naïve cases had a lower AD in the body of the corpus callosum using ComBat and after adjusting for covariates *B[SE]* = 0.25[0.12], *P* = 0.04; see supplementary Table 6). When adjusting for the site in the mixed model analysis, medication-naïve OCD cases showed a lower ND across multiple tracts (Supplementary Table 7). Bayesian results are shown in supplementary Fig. 2, indicating lower microstructural integrity in medication-naïve OCD cases across multiple tracts but none with *P+* values > 0.9.

**Table 2:** Case-Control mixed model analyses of white matter microstructure – ComBat corrected.

	HC	OCD	Group difference (crude model)			Group difference (adjusted model)*		
	M ± SD	M ± SD	B [SE]	95% CI	P (unc)	B [SE]	95% CI	*P* (unc)
**CC genu**								
Overall diffusion			0.046 [0.050]	−0.06 | 0.15	0.905 (0.389)	0.023 [0.050]	−0.08 | 0.12	0.940 (0.652)
AD	1.522 ± 0.052	1.523 ± 0.050	0.004 [0.090]	−0.17 | 0.18	0.963 (0.963)	−0.019 [0.090]	−0.19 | 0.15	0.831 (0.831)
FA	0.749 ± 0.036	0.753 ± 0.034	0.096 [0.090]	−0.08 | 0.27	0.664 (0.279)	0.073 [0.090]	−0.10 | 0.24	0.753 (0.403)
MD	0.732 ± 0.035	0.730 ± 0.036	0.052 [0.090]	−0.12 | 0.22	0.638 (0.559)	0.029 [0.090]	−0.14 | 0.20	0.833 (0.740)
RD	0.339 ± 0.046	0.336 ± 0.044	0.073 [0.090]	−0.10 | 0.25	0.608 (0.410)	0.050 [0.090]	−0.12 | 0.22	0.726 (0.565)
ND	0.646 ± 0.040	0.649 ± 0.038	0.082 [0.090]	−0.09 | 0.26	0.898 (0.355)	0.059 [0.090]	−0.11 | 0.23	0.906 (0.499)
OD	0.086 ± 0.009	0.086 ± 0.010	−0.032 [0.090]	−0.20 | 0.14	0.833 (0.720)	−0.054 [0.090]	−0.22 | 0.12	0.879 (0.533)
**CC body**								
Overall diffusion			0.083 [0.050]	−0.02 | 0.19	0.905 (0.127)	0.068 [0.050]	−0.04 | 0.17	0.940 (0.211)
AD	1.508 ± 0.046	1.512 ± 0.046	0.086 [0.090]	−0.09 | 0.26	0.454 (0.333)	0.070 [0.090]	−0.10 | 0.24	0.570 (0.426)
FA	0.735 ± 0.042	0.742 ± 0.037	0.172 [0.090]	0.00 | 0.34	0.468 (0.052)	0.156 [0.090]	−0.02 | 0.33	0.693 (0.077)
MD	0.727 ± 0.035	0.725 ± 0.033	0.051 [0.090]	−0.12 | 0.22	0.638 (0.567)	0.035 [0.090]	−0.14 | 0.21	0.833 (0.688)
RD	0.353 ± 0.051	0.347 ± 0.047	0.135 [0.090]	−0.04 | 0.31	0.603 (0.126)	0.120 [0.090]	−0.05 | 0.29	0.690 (0.174)
ND	0.655 ± 0.034	0.656 ± 0.033	0.026 [0.090]	−0.15 | 0.20	0.898 (0.772)	0.010 [0.090]	−0.16 | 0.18	0.906 (0.906)
OD	0.083 ± 0.009	0.083 ± 0.009	0.030 [0.090]	−0.14 | 0.20	0.833 (0.735)	0.015 [0.090]	−0.16 | 0.19	0.879 (0.867)
**CC splenium**								
Overall diffusion			0.029 [0.060]	−0.08 | 0.14	0.944 (0.604)	0.016 [0.060]	−0.09 | 0.12	0.940 (0.780)
AD	1.586 ± 0.051	1.591 ± 0.051	0.091 [0.090]	−0.08 | 0.26	0.454 (0.303)	0.077 [0.090]	−0.10 | 0.25	0.570 (0.380)
FA	0.834 ± 0.024	0.835 ± 0.021	0.073 [0.090]	−0.10 | 0.25	0.664 (0.411)	0.059 [0.090]	−0.11 | 0.23	0.753 (0.502)
MD	0.699 ± 0.023	0.699 ± 0.026	−0.001 [0.090]	−0.17 | 0.17	0.990 (0.990)	−0.015 [0.090]	−0.19 | 0.16	0.868 (0.868)
RD	0.243 ± 0.030	0.242 ± 0.027	0.049 [0.090]	−0.12 | 0.22	0.650 (0.578)	0.036 [0.090]	−0.14 | 0.21	0.773 (0.687)
ND	0.724 ± 0.031	0.724 ± 0.03	−0.011 [0.090]	−0.18 | 0.16	0.898 (0.898)	−0.025 [0.090]	−0.2 | 0.15	0.906 (0.778)
OD	0.070 ± 0.010	0.070 ± 0.010	−0.025 [0.090]	−0.20 | 0.15	0.833 (0.775)	−0.039 [0.090]	−0.21 | 0.13	0.879 (0.659)
**Dorsal cingulum**								
Overall diffusion			0.026 [0.070]	−0.11 | 0.16	0.944 (0.702)	0.025 [0.060]	−0.10 | 0.15	0.940 (0.703)
AD	1.269 ± 0.057	1.274 ± 0.055	0.102 [0.090]	−0.07 | 0.28	0.454 (0.248)	0.101 [0.090]	−0.07 | 0.27	0.570 (0.243)
FA	0.588 ± 0.045	0.591 ± 0.044	0.068 [0.090]	−0.10 | 0.24	0.664 (0.443)	0.067 [0.090]	−0.10 | 0.24	0.753 (0.441)
MD	0.720 ± 0.025	0.722 ± 0.026	−0.068 [0.090]	−0.24 | 0.10	0.638 (0.444)	−0.069 [0.090]	−0.24 | 0.10	0.710 (0.430)
RD	0.445 ± 0.038	0.445 ± 0.037	−0.012 [0.090]	−0.18 | 0.16	0.896 (0.896)	−0.012 [0.090]	−0.18 | 0.16	0.887 (0.887)
ND	0.598 ± 0.033	0.598 ± 0.033	0.016 [0.090]	−0.16 | 0.19	0.898 (0.860)	0.015 [0.090]	−0.16 | 0.18	0.906 (0.866)
OD	0.138 ± 0.018	0.138 ± 0.017	0.048 [0.090]	−0.12 | 0.22	0.833 (0.584)	0.048 [0.090]	−0.12 | 0.22	0.879 (0.584)
**Ventral cingulum**								
Overall diffusion			0.002 [0.060]	−0.11 | 0.11	0.968 (0.968)	0.004 [0.050]	−0.10 | 0.11	0.940 (0.940)
AD	1.247 ± 0.082	1.255 ± 0.079	0.101 [0.090]	−0.07 | 0.27	0.454 (0.255)	0.103 [0.090]	−0.07 | 0.27	0.570 (0.240)
FA	0.490 ± 0.0470	0.490 ± 0.044	0.001 [0.090]	−0.17 | 0.17	0.988 (0.988)	0.003 [0.090]	−0.17 | 0.17	0.997 (0.971)
MD	0.745 ± 0.051	0.749 ± 0.044	−0.087 [0.090]	−0.26 | 0.09	0.638 (0.327)	−0.085 [0.090]	−0.26 | 0.09	0.710 (0.332)
RD	0.49 ± 0.053	0.493 ± 0.047	−0.063 [0.090]	−0.24 | 0.11	0.608 (0.473)	−0.062 [0.090]	−0.23 | 0.11	0.720 (0.480)
ND	0.508 ± 0.039	0.506 ± 0.035	−0.034 [0.090]	−0.21 | 0.14	0.898 (0.705)	−0.032 [0.090]	−0.20 | 0.14	0.906 (0.717)
OD	0.166 ± 0.022	0.164 ± 0.022	0.095 [0.090]	−0.08 | 0.27	0.833 (0.283)	0.097 [0.090]	−0.07 | 0.27	0.879 (0.267)
**Posterior thal rad**								
Overall diffusion			−0.047 [0.060]	−0.16 | 0.06	0.905 (0.402)	−0.051 [0.060]	−0.16 | 0.06	0.940 (0.352)
AD	1.370 ± 0.049	1.374 ± 0.045	0.086 [0.090]	−0.09 | 0.26	0.454 (0.332)	0.081 [0.090]	−0.09 | 0.25	0.570 (0.354)
FA	0.607 ± 0.030	0.605 ± 0.031	−0.070 [0.090]	−0.24 | 0.10	0.664 (0.427)	−0.075 [0.090]	−0.25 | 0.10	0.753 (0.396)
MD	0.771 ± 0.030	0.775 ± 0.029	−0.142 [0.090]	−0.32 | 0.03	0.638 (0.109)	−0.146 [0.090]	−0.32 | 0.03	0.710 (0.096)
RD	0.465 ± 0.034	0.469 ± 0.035	−0.118 [0.090]	−0.29 | 0.06	0.603 (0.183)	−0.122 [0.090]	−0.29 | 0.05	0.690 (0.164)
ND	0.569 ± 0.037	0.567 ± 0.036	−0.056 [0.090]	−0.23 | 0.12	0.898 (0.530)	−0.060 [0.090]	−0.23 | 0.11	0.906 (0.495)
OD	0.119 ± 0.012	0.119 ± 0.012	0.019 [0.090]	−0.15 | 0.19	0.833 (0.833)	0.014 [0.090]	−0.16 | 0.19	0.879 (0.872)
**Sag Stratum**								
Overall diffusion			−0.012 [0.060]	−0.13 | 0.11	0.944 (0.839)	−0.009 [0.060]	−0.13 | 0.11	0.940 (0.886)
AD	1.323 ± 0.046	1.329 ± 0.043	0.133 [0.090]	−0.04 | 0.31	0.454 (0.133)	0.136 [0.090]	−0.04 | 0.31	0.570 (0.125)
FA	0.571 ± 0.031	0.570 ± 0.032	−0.004 [0.090]	−0.18 | 0.17	0.988 (0.965)	−0.000 [0.090]	−0.17 | 0.17	0.997 (0.997)
MD	0.778 ± 0.027	0.781 ± 0.028	−0.124 [0.090]	−0.30 | 0.05	0.638 (0.160)	−0.121 [0.090]	−0.29 | 0.05	0.710 (0.174)
RD	0.510 ± 0.034	0.513 ± 0.034	−0.067 [0.090]	−0.24 | 0.11	0.608 (0.449)	−0.063 [0.090]	−0.24 | 0.11	0.720 (0.475)
ND	0.545 ± 0.034	0.542 ± 0.035	−0.061 [0.090]	−0.23 | 0.11	0.898 (0.492)	−0.057 [0.090]	−0.23 | 0.12	0.906 (0.520)
OD	0.142 ± 0.013	0.141 ± 0.012	0.050 [0.090]	−0.12 | 0.22	0.833 (0.575)	0.053 [0.090]	−0.12 | 0.23	0.879 (0.550)
**SLF**								
Overall diffusion			−0.013 [0.060]	−0.13 | 0.11	0.944 (0.835)	−0.027 [0.060]	−0.15 | 0.09	0.940 (0.659)
AD	1.117 ± 0.032	1.119 ± 0.034	0.082 [0.090]	−0.09 | 0.26	0.454 (0.353)	0.068 [0.090]	−0.10 | 0.24	0.570 (0.443)
FA	0.512 ± 0.028	0.511 ± 0.026	−0.031 [0.090]	−0.20 | 0.14	0.936 (0.728)	−0.045 [0.090]	−0.22 | 0.13	0.784 (0.610)
MD	0.693 ± 0.022	0.694 ± 0.022	−0.068 [0.090]	−0.24 | 0.10	0.638 (0.442)	−0.082 [0.090]	−0.26 | 0.09	0.710 (0.352)
RD	0.480 ± 0.028	0.482 ± 0.026	−0.070 [0.090]	−0.24 | 0.10	0.608 (0.430)	−0.084 [0.090]	−0.26 | 0.09	0.720 (0.341)
ND	0.672 ± 0.031	0.672 ± 0.031	−0.019 [0.090]	−0.19 | 0.15	0.898 (0.833)	−0.033 [0.090]	−0.21 | 0.14	0.906 (0.709)
OD	0.196 ± 0.012	0.195 ± 0.013	0.028 [0.090]	−0.14 | 0.20	0.833 (0.753)	0.013 [0.090]	−0.16 | 0.19	0.879 (0.879)
**uncF**								
Overall diffusion			0.079 [0.060]	−0.05 | 0.21	0.905 (0.228)	0.072 [0.070]	−0.06 | 0.20	0.940 (0.272)
AD	1.246 ± 0.061	1.248 ± 0.052	0.026 [0.090]	−0.15 | 0.20	0.864 (0.768)	0.020 [0.090]	−0.15 | 0.19	0.831 (0.825)
FA	0.503 ± 0.049	0.508 ± 0.046	0.112 [0.090]	−0.06 | 0.28	0.664 (0.205)	0.106 [0.090]	−0.07 | 0.28	0.753 (0.234)
MD	0.767 ± 0.033	0.765 ± 0.032	0.070 [0.090]	−0.10 | 0.24	0.638 (0.427)	0.064 [0.090]	−0.11 | 0.24	0.710 (0.473)
RD	0.532 ± 0.045	0.527 ± 0.044	0.113 [0.090]	−0.06 | 0.29	0.603 (0.201)	0.107 [0.090]	−0.07 | 0.28	0.690 (0.230)
ND	0.503 ± 0.032	0.506 ± 0.032	0.084 [0.090]	−0.09 | 0.26	0.898 (0.341)	0.078 [0.090]	−0.10 | 0.25	0.906 (0.382)
OD	0.154 ± 0.019	0.152 ± 0.016	0.066 [0.090]	−0.11 | 0.24	0.833 (0.458)	0.059 [0.090]	−0.11 | 0.23	0.879 (0.506)

Reported Tensor/NODDI measures are adjusted for site using Combat and Z-transformed. MD, RD and OD values are inverted so that higher values represent better microstructural integrity. False Discovery rate (FDR) significant differences are marked in bold. *P* < 0.05, uncorrected significant results are underlined. *Corrected for age, sex and education in years; *FA* fractional anisotropy, *MD* mean diffusivity, *RD* radial diffusivity, *AD* axial diffusivity, *ND* neurite density, *OD* orientation dispersion, *SLF* superior longitudinal fascicle, *CC genu* genu of the corpus callosum, *CC body* body of the corpus callosum, *CC splenium* splenium of the corpus callosum, *uncF* uncinate fascicle, *Sag Stratum* sagittal stratum, *Posterior thal rad* posterior thalamic radiation.

**Table 3:** Age of OCD onset mixed model analyses of white matter microstructure – ComBat.

	Adult	Child	Group difference (crude model)			Group difference (adjusted model)*		
	M ± SD	M ± SD	B [SE]	95% CI	P (unc)	B [SE]	95% CI	*P* (unc)
**CC genu**								
Overall diffusion			0.119 [0.070]	−0.03 | 0.26	0.222 (0.111)	0.035 [0.070]	−0.10 | 0.17	0.804 (0.625)
AD	1.521 ± 0.051	1.524 ± 0.050	0.066 [0.120]	−0.18 | 0.31	0.813 (0.592)	−0.017 [0.120]	−0.26 | 0.22	0.925 (0.888)
FA	0.751 ± 0.036	0.754 ± 0.031	0.105 [0.120]	−0.14 | 0.35	0.512 (0.394)	0.022 [0.120]	−0.22 | 0.26	0.964 (0.857)
MD	0.732 ± 0.038	0.729 ± 0.034	0.095 [0.120]	−0.15 | 0.34	0.627 (0.445)	0.011 [0.120]	−0.23 | 0.25	0.966 (0.927)
RD	0.338 ± 0.046	0.334 ± 0.042	0.098 [0.120]	−0.14 | 0.34	0.559 (0.430)	0.014 [0.120]	−0.22 | 0.25	0.907 (0.907)
ND	0.645 ± 0.040	0.652 ± 0.037	0.189 [0.120]	−0.05 | 0.43	0.223 (0.127)	0.105 [0.120]	−0.13 | 0.34	0.500 (0.389)
OD	0.087 ± 0.010	0.086 ± 0.010	0.158 [0.120]	−0.08 | 0.40	0.751 (0.201)	0.075 [0.120]	−0.16 | 0.31	0.931 (0.541)
**CC body**								
Overall diffusion			0.127 [0.070]	−0.01 | 0.27	0.222 (0.081)	0.096 [0.070]	−0.05 | 0.24	0.594 (0.198)
AD	1.511 ± 0.047	1.513 ± 0.045	0.043 [0.120]	−0.19 | 0.28	0.813 (0.723)	0.012 [0.120]	−0.23 | 0.25	0.925 (0.925)
FA	0.739 ± 0.036	0.744 ± 0.039	0.125 [0.120]	−0.11 | 0.36	0.512 (0.304)	0.093 [0.120]	−0.14 | 0.33	0.716 (0.445)
MD	0.727 ± 0.032	0.723 ± 0.034	0.100 [0.120]	−0.14 | 0.34	0.627 (0.411)	0.068 [0.120]	−0.17 | 0.31	0.897 (0.577)
RD	0.349 ± 0.045	0.345 ± 0.048	0.086 [0.120]	−0.15 | 0.32	0.559 (0.477)	0.055 [0.120]	−0.18 | 0.29	0.735 (0.653)
ND	0.652 ± 0.032	0.660 ± 0.033	0.221 [0.120]	−0.02 | 0.46	0.223 (0.069)	0.189 [0.120]	−0.05 | 0.43	0.328 (0.122)
OD	0.084 ± 0.009	0.082 ± 0.009	0.188 [0.120]	−0.05 | 0.43	0.751 (0.122)	0.157 [0.120]	−0.08 | 0.40	0.931 (0.200)
**CC splenium**								
Overall diffusion			0.109 [0.070]	−0.04 | 0.26	0.222 (0.149)	0.057 [0.070]	−0.09 | 0.20	0.673 (0.449)
AD	1.588 ± 0.053	1.594 ± 0.05	0.118 [0.120]	−0.12 | 0.36	0.813 (0.344)	0.066 [0.120]	−0.18 | 0.31	0.925 (0.597)
FA	0.834 ± 0.021	0.836 ± 0.02	0.105 [0.120]	−0.14 | 0.35	0.512 (0.398)	0.053 [0.120]	−0.19 | 0.30	0.860 (0.669)
MD	0.700 ± 0.027	0.698 ± 0.025	0.047 [0.120]	−0.20 | 0.29	0.708 (0.708)	−0.005 [0.120]	−0.25 | 0.24	0.966 (0.966)
RD	0.244 ± 0.028	0.24 ± 0.026	0.132 [0.120]	−0.11 | 0.38	0.559 (0.289)	0.080 [0.120]	−0.16 | 0.32	0.735 (0.520)
ND	0.722 ± 0.031	0.725 ± 0.03	0.113 [0.120]	−0.13 | 0.36	0.407 (0.362)	0.062 [0.120]	−0.18 | 0.30	0.692 (0.621)
OD	0.071 ± 0.01	0.069 ± 0.01	0.139 [0.120]	−0.1 | 0.38	0.751 (0.264)	0.087 [0.120]	−0.16 | 0.33	0.931 (0.484)
**Dorsal cingulum**								
Overall diffusion			0.063 [0.090]	−0.12 | 0.25	0.567 (0.504)	0.026 [0.090]	−0.16 | 0.21	0.807 (0.782)
AD	1.279 ± 0.057	1.271 ± 0.053	−0.133 [0.120]	−0.38 | 0.11	0.813 (0.284)	−0.170 [0.120]	−0.41 | 0.07	0.747 (0.166)
FA	0.590 ± 0.045	0.592 ± 0.043	0.033 [0.120]	−0.21 | 0.28	0.790 (0.790)	−0.004 [0.120]	−0.24 | 0.24	0.973 (0.973)
MD	0.724 ± 0.027	0.719 ± 0.025	0.202 [0.120]	−0.04 | 0.44	0.234 (0.104)	0.165 [0.120]	−0.07 | 0.40	0.407 (0.181)
RD	0.447 ± 0.038	0.443 ± 0.036	0.109 [0.120]	−0.13 | 0.35	0.559 (0.380)	0.072 [0.120]	−0.17 | 0.31	0.735 (0.559)
ND	0.595 ± 0.032	0.601 ± 0.033	0.180 [0.120]	−0.06 | 0.42	0.223 (0.149)	0.142 [0.120]	−0.1 | 0.38	0.404 (0.248)
OD	0.137 ± 0.018	0.138 ± 0.016	−0.013 [0.120]	−0.26 | 0.23	0.952 (0.916)	−0.050 [0.120]	−0.29 | 0.19	0.931 (0.682)
**Ventral cingulum**								
Overall diffusion			−0.156 [0.080]	−0.30 | −0.01	0.184 (0.041)	−0.137 [0.070]	−0.28 | 0.01	0.387 (0.070)
AD	1.252 ± 0.081	1.258 ± 0.078	0.068 [0.120]	−0.17 | 0.30	0.813 (0.570)	0.087 [0.120]	−0.15 | 0.32	0.925 (0.466)
FA	0.496 ± 0.043	0.486 ± 0.045	−0.212 [0.120]	−0.45 | 0.02	0.351 (0.078)	−0.193 [0.120]	−0.43 | 0.04	0.482 (0.107)
MD	0.743 ± 0.046	0.753 ± 0.042	−0.219 [0.120]	−0.45 | 0.02	0.204 (0.068)	−0.200 [0.120]	−0.43 | 0.03	0.407 (0.095)
RD	0.483 ± 0.046	0.500 ± 0.047	−0.336 [0.120]	−0.57 | −0.1	**0.045** (0.005)	−0.317 [0.120]	−0.55 | −0.08	0.072 (0.008)
ND	0.510 ± 0.033	0.503 ± 0.037	−0.195 [0.120]	−0.43 | 0.04	0.223 (0.105)	−0.176 [0.120]	−0.41 | 0.06	0.328 (0.142)
OD	0.163 ± 0.021	0.164 ± 0.022	−0.042 [0.120]	−0.28 | 0.19	0.952 (0.728)	−0.023 [0.120]	−0.26 | 0.21	0.931 (0.849)
**Posterior thal rad**								
Overall diffusion			0.201 [0.080]	0.04 | 0.36	0.108 (0.012)	0.136 [0.080]	−0.02 | 0.29	0.387 (0.086)
AD	1.374 ± 0.046	1.374 ± 0.044	0.005 [0.120]	−0.24 | 0.25	0.969 (0.969)	−0.060 [0.120]	−0.30 | 0.18	0.925 (0.626)
FA	0.600 ± 0.031	0.609 ± 0.032	0.291 [0.120]	0.05 | 0.53	0.171 (0.019)	0.226 [0.120]	−0.01 | 0.47	0.482 (0.067)
MD	0.779 ± 0.03	0.772 ± 0.028	0.245 [0.120]	0.00 | 0.49	0.204 (0.048)	0.180 [0.120]	−0.06 | 0.42	0.407 (0.145)
RD	0.474 ± 0.035	0.465 ± 0.035	0.278 [0.120]	0.04 | 0.52	0.112 (0.025)	0.213 [0.120]	−0.03 | 0.45	0.382 (0.085)
ND	0.561 ± 0.036	0.571 ± 0.035	0.285 [0.120]	0.04 | 0.53	0.198 (0.022)	0.220 [0.120]	−0.02 | 0.46	0.328 (0.076)
OD	0.120 ± 0.012	0.119 ± 0.012	0.101 [0.120]	−0.14 | 0.34	0.751 (0.417)	0.036 [0.120]	−0.20 | 0.28	0.931 (0.774)
**Sag Stratum**								
Overall diffusion			0.116 [0.090]	−0.05 | 0.28	0.222 (0.173)	0.089 [0.090]	−0.08 | 0.26	0.635 (0.306)
AD	1.327 ± 0.043	1.330 ± 0.044	0.070 [0.120]	−0.17 | 0.31	0.813 (0.575)	0.043 [0.130]	−0.20 | 0.29	0.925 (0.733)
FA	0.567 ± 0.032	0.573 ± 0.031	0.183 [0.120]	−0.06 | 0.43	0.425 (0.144)	0.156 [0.130]	−0.09 | 0.40	0.654 (0.218)
MD	0.782 ± 0.028	0.780 ± 0.027	0.087 [0.120]	−0.16 | 0.33	0.627 (0.488)	0.060 [0.130]	−0.19 | 0.30	0.897 (0.637)
RD	0.514 ± 0.035	0.511 ± 0.034	0.085 [0.120]	−0.16 | 0.33	0.559 (0.497)	0.058 [0.130]	−0.19 | 0.30	0.735 (0.647)
ND	0.539 ± 0.035	0.545 ± 0.036	0.167 [0.120]	−0.08 | 0.41	0.235 (0.183)	0.140 [0.130]	−0.11 | 0.39	0.404 (0.269)
OD	0.142 ± 0.011	0.141 ± 0.013	0.103 [0.120]	−0.14 | 0.35	0.751 (0.410)	0.076 [0.130]	−0.17 | 0.32	0.931 (0.548)
**SLF**								
Overall diffusion			0.116 [0.080]	−0.05 | 0.28	0.222 (0.166)	0.078 [0.080]	−0.09 | 0.24	0.635 (0.353)
AD	1.122 ± 0.034	1.117 ± 0.034	−0.168 [0.120]	−0.41 | 0.08	0.813 (0.178)	−0.205 [0.120]	−0.45 | 0.04	0.747 (0.101)
FA	0.509 ± 0.026	0.513 ± 0.025	0.164 [0.120]	−0.08 | 0.41	0.425 (0.189)	0.126 [0.120]	−0.12 | 0.37	0.704 (0.313)
MD	0.698 ± 0.022	0.691 ± 0.022	0.278 [0.120]	0.04 | 0.52	0.204 (0.026)	0.240 [0.120]	0.00 | 0.48	0.407 (0.055)
RD	0.485 ± 0.026	0.479 ± 0.025	0.222 [0.120]	−0.02 | 0.47	0.222 (0.074)	0.185 [0.120]	−0.06 | 0.43	0.420 (0.140)
ND	0.668 ± 0.031	0.675 ± 0.030	0.219 [0.120]	−0.02 | 0.46	0.223 (0.079)	0.182 [0.120]	−0.06 | 0.42	0.328 (0.146)
OD	0.195 ± 0.013	0.195 ± 0.012	−0.019 [0.120]	−0.26 | 0.22	0.952 (0.877)	−0.057 [0.120]	−0.30 | 0.19	0.931 (0.649)
**uncF**								
Overall diffusion			−0.019 [0.090]	−0.19 | 0.15	0.832 (0.832)	−0.022 [0.090]	−0.20 | 0.15	0.807 (0.807)
AD	1.247 ± 0.054	1.249 ± 0.049	0.044 [0.120]	−0.19 | 0.28	0.813 (0.717)	0.040 [0.120]	−0.20 | 0.28	0.925 (0.741)
FA	0.510 ± 0.046	0.507 ± 0.045	−0.083 [0.120]	−0.32 | 0.15	0.551 (0.490)	−0.086 [0.120]	−0.32 | 0.15	0.716 (0.477)
MD	0.766 ± 0.035	0.764 ± 0.029	0.050 [0.120]	−0.18 | 0.28	0.708 (0.675)	0.047 [0.120]	−0.19 | 0.28	0.897 (0.698)
RD	0.525 ± 0.048	0.528 ± 0.040	−0.070 [0.120]	−0.3 | 0.16	0.560 (0.560)	−0.073 [0.120]	−0.31 | 0.16	0.735 (0.546)
ND	0.506 ± 0.033	0.505 ± 0.031	−0.045 [0.120]	−0.28 | 0.19	0.709 (0.709)	−0.048 [0.120]	−0.28 | 0.19	0.692 (0.692)
OD	0.152 ± 0.015	0.152 ± 0.017	−0.007 [0.120]	−0.24 | 0.23	0.952 (0.952)	−0.011 [0.120]	−0.25 | 0.23	0.931 (0.931)

Reported Tensor/NODDI measures are adjusted for site using Combat and Z-transformed. MD, RD and OD values are inverted so that higher values represent better microstructural integrity. False Discovery rate (FDR) significant differences are marked in bold. *P* < 0.05, uncorrected significant results are underlined. *Corrected for age, sex and education in years; *FA* fractional anisotropy, *MD* mean diffusivity, *RD* radial diffusivity, *AD* axial diffusivity, *ND* neurite density, *OD* orientation dispersion, *SLF* superior longitudinal fascicle, *CC genu* genu of the corpus callosum, *CC body* body of the corpus callosum, *CC splenium* splenium of the corpus callosum, *uncF* uncinate fascicle, *Sag Stratum* sagittal stratum, *Posterior thal rad* posterior thalamic radiation.

Consistent with the tract-based analyses, analyses of the skeletonized white matter voxels within the entire JHU-ICBM-DTI-81 atlas (with randomise) showed widespread lower ND in late onset (compared with early onset) cases involving amongst others the corpus callosum, SLF, coronal radiation, PTR and SagS, but predominantly on the right side (TFCE, *P*_*FW*E_ < 0.05; See supplementary Fig. 3). There were no case-control differences nor between cases with and without a history of SSRI/SNRI use.

Associations with clinical measures are reported in supplementary tables 8–11. After adjusting for age, sex and educational level and multiple comparisons, we observed a positive association between duration of illness and ND in the uncinate fascicle (*B[SE]* = 0.018[0.01], *P*_*FDR*_ = 0.03). There were no significant associations with the subscales of the D-YBOCS.

### White matter fiber density and cross-section

**Fig. 2:**
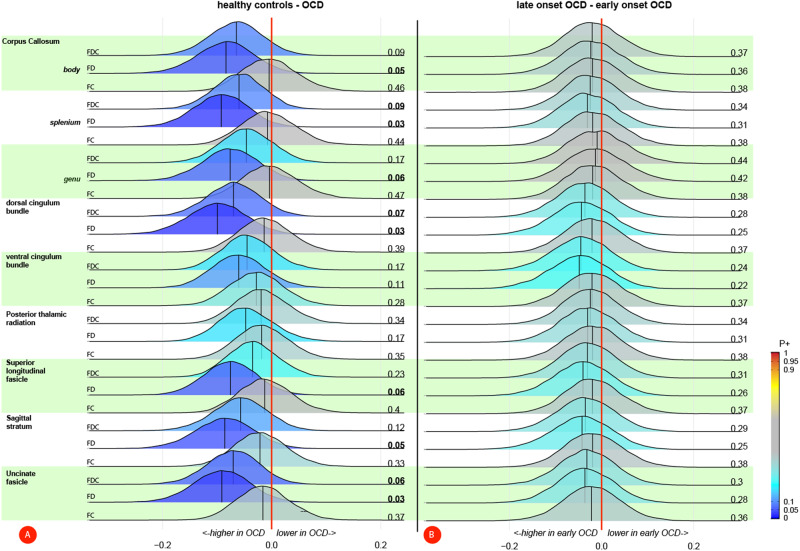
Bayesian posterior distribution plots on fixel-based measures between (a) OCD patients and healthy controls and (b) early and late onset OCD patients. **A** The posterior distributions showed credible evidence for a higher FD (and to a lesser extent FDC) in individuals with OCD compared with healthy controls in the majority of the tracts. There was no credible evidence for a difference in FC. **B** Credibility for a difference in fixel-based measures between early and late onset OCD patients was low. Posterior probabilities of a positive effect (P+) are shown next to each distribution and color-coded. *P*+ values ≥ 0.90 (moderate to very high credibility for a positive effect) or ≤0.10 (moderate to very high credibility for a negative effect) are presented in bold. FDC fiber density and cross-section, FD fiber density, FC fiber cross-section.

After adjusting for covariates, FD in the SagS (*B[SE]* = 0.12[0.05], *P* = 0.02) was higher in individuals with OCD compared with HC, but no comparison survived the FDR correction, neither when correcting for site using ComBat (supplementary Table 12) nor within the mixed model analysis (supplementary Table 13). The Bayesian multilevel analysis showed moderate to very high credibility for a higher FD in OCD compared with HC in all tracts but the ventral cingulum bundle and PTR (see Fig. 2A). Neither the mixed-model nor Bayesian multilevel analysis showed evidence for differences between early onset and late onset OCD (supplementary Tables 14–15; Fig. 2B). Comparing OCD cases with and without a prior exposure to SSRI/SNRI showed a lower FD (*B[SE]* = −0.27[0.12], *P* = 0.03; supplementary Tables 16–17) in the uncinate fascicle in medication-naïve OCD cases, but these results did not survive FDR correction and credibility for these findings was relatively low (see supplementary Fig. 4). The whole atlas-based fixel analysis also showed no differences between cases and controls, OCD cases with different ages of onset or SSRI/SNRI exposure and there were no linear associations with age of onset, illness duration or severity (supplementary Tables 18–20). After multiple comparison corrections, there were also no significant associations with the subscales of the D-YBOCS (supplementary Table 21)

### Structural Connectome

**Fig. 3:**
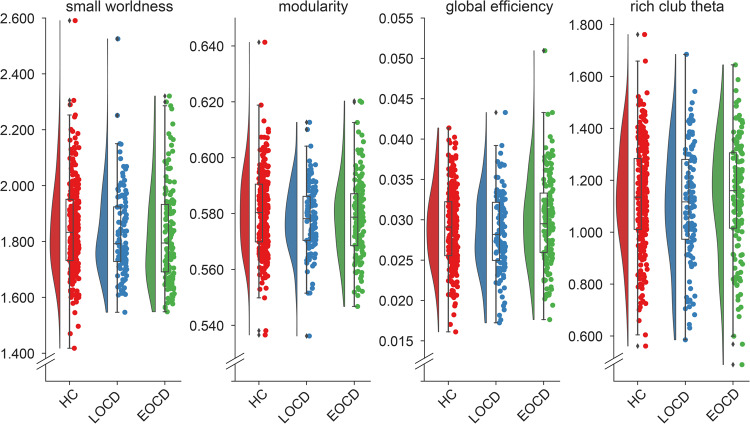
Raincloud plots of global connectome measures of healthy controls, early and late onset OCD patients. Early onset OCD patients showed a significantly higher global efficiency compared with late onset patients. abbreviations: HC healthy controls, LOCD Late onset OCD, EOCD early onset OCD.

There were no case-control connectome differences on either the global or nodal level (Supplementary Table 22; Fig. 3). After adjusting for covariates, global efficiency was lower in late onset (compared with early onset) OCD (*B[SE]* = 14.8[6.7], *P* = 0.03; Supplementary Table 23; Fig. 3). There were no differences in the other global measures, no differences in OCD cases with or without prior SSRI/SNRI exposure (supplementary table 24), no associations with clinical variables (supplementary table 25–27) and none of the comparisons on the nodal measures survived multiple comparison correction. Modelling site as a random effect gave the same results (supplementary table 28–30).

### Post-hoc analysis

To better understand the observed differences between the early and late onset of OCD we additionally compared the tensor/NODDI, fixel, and global connectome measures of both OCD onset groups to HC. For the tensor/NODDI and fixel measures we used the Bayesian multilevel analysis to combine all ComBat corrected measures across the tracts into one model to limit the number of additional analyses, while we used ANCOVA’s for the four global connectome measures. All analyses were adjusted for age, sex and education. Analysis of the tensor/NODDI measures showed differential direction of effect of the two OCD groups relative to HC, with early onset OCD cases primarily showing *higher* microstructural integrity relative to HC; predominantly in the PTR, SLF and SagS, while late-onset OCD cases generally showed *lower* microstructural integrity relative to HC (supplementary Fig. 5). Fiber density and cross-section were generally higher in both OCD groups compared with HC, with slightly higher credibility for the early onset group (supplementary Fig. 6). Although global efficiency was higher in the early onset group and lower in the late-onset group relative to HC, neither comparison reached statistical significance (early onset vs. healthy control: *P* = 0.10, late onset vs. HC: *P* = 0.21).

## Discussion

The main finding of this dMRI study of the OCD global study involving a large group of medication-free adults with OCD and HC from five different international sites was that, relative to early-onset OCD cases, late-onset OCD cases show a lower capacity to integrate information across the network (i.e., lower efficiency) and lower integrity of associative white matter tracts, particularly the PTR. Voxel-based analysis of the skeletonized atlas showed even more widespread right lateralized reductions in ND in late-onset OCD. There were no case-control differences in microstructure or network topology, except for a higher fiber density of the SagS in OCD individuals that did not survive multiple comparison corrections but did show high credibility in the Bayesian analysis. We also observed differences between individuals with OCD and with and without a history of SSRI/SNRI use although between-group differences in comorbidities likely confound these results and all participants were free from current SSRI/SNRI medication.

The PTR—which includes the optic radiation—consists of reciprocal axons between the posterior thalamic regions and the parietal and occipital regions. Its integrity has previously been associated with better cognitive control and the ability to suppress interfering information in children [35]. In the ENIGMA-OCD study [2], it was FA in the PTR that showed the highest effect size in the case-control comparisons (Cohen’s d = −0.26). While we did not observe a case-control difference when considering all individuals with OCD together, our post-hoc Bayesian analyses showed differential directions of the effect of the early and late OCD onset groups relative to HC. Late-onset OCD cases exhibited lower microstructural integrity of PTR (particularly FA, MD, ND and RD) whereas early-onset OCD cases show higher microstructural integrity relative to HC. We saw similar patterns for the SLF. Piras and colleagues, unfortunately, did not report any age of onset group comparisons, but they did not observe any *linear* associations with age of onset. In Piras et al.‘s study, mean OCD onset was 19.1 ± 8.4 years, while in our study, it was 17.3 ± 7.1 years for the overall sample and 23.4 ± 5.6 years for late-onset OCD cases. Severity and age at study participation were comparable between our late-onset OCD cases and the complete ENIGMA-OCD sample. The individuals with OCD in the ENIGMA-OCD study may resemble our late-onset cases more closely, but without additional detailed clinical information, this remains speculative. In the absence of differences in OCD symptom severity, sex distribution, medication history or psychiatric comorbidities between our early and late OCD onset cases, our results suggest that – at least for the microstructure of the PTR—individuals with either early or late OCD onset represent distinct groups with different underlying neurobiology. Axonal myelination and synaptic pruning continue throughout adolescence and young adulthood and follow a complex spatial and temporal pattern across neurodevelopment [36, 37]. Disruption of these processes at a specific time might, therefore, differentially affect the normal development of the PTR and other white matter fiber bundles. Whether it is developmental dysregulation that leads to OCD, or the onset of OCD that disrupts the normal process is unknown. Interestingly, a recent UK Biobank study showed that highly stressful life events during either childhood or adulthood had differential effects on the brain and cognition, including the microstructural integrity of the PTR [38], which may give credence to the latter direction of events.

The PTR connects different cortical regions with the thalamus, a brain structure that has been repeatedly implicated in the pathophysiology of OCD, particularly in morphometric studies. A previous ENIGMA-OCD study showed that the thalamus is *larger* in pediatric OCD cases and *smaller* in late OCD cases, relative to their respective matched HC [39]. OCD cases with a late onset also had a smaller thalamus, while individuals with early onset OCD showed no differences. Together these studies may suggest that the emergence of OCD at different lifespan periods has a differential effect on circuitry that involves the thalamus and surrounding white matter fiber bundles, but the mechanism and clinical ramifications are still unclear. Analyses of potential morphometric differences in the OCD global sample are ongoing (osf.io/bvywf). The PTR also contains afferents to the visual cortices and therefore these findings lend support to the growing body of literature that implicates visual (attentional) processes in the pathophysiology of OCD [40–42].

Our Bayesian (but not multiple comparisons corrected NHST) analyses showed credible evidence for higher fiber density in the SagS and several other tracts in OCD compared with HC. The OCD-related higher fiber density seems at odds with the previously reported lower FA in the SagS [2]. To the best of our knowledge, no previous study has performed fixel-based analyses in OCD. However, a recent study in a large sample of typically developing youth showed associations between the severity of symptom dimensions of OCD (e.g., repetition/checking) and fixel measures in the splenium of the corpus callosum, while associations with FA were found in spatially distinct brain regions [41]. The authors argued that FA and fixel measures may represent different features of the white matter and showed low correlations between tract-specific FA and fixel measures. Indeed, we also observed low correlations between the tensor/NODDI-based measures and fixel measures (Supplementary Fig. 7). The low consistency between tensor-based (e.g., FA) and fixel measures may be especially true for white matter areas that are rich in kissing or crossing fibers as tensor-based measures provide an average across the entire voxel, whereas in fixel-based analysis, measures are calculated for specific fiber bundles within a voxel [27, 43]. The SagS is not one bundle but a complex crossroads of different associational fibers. Two recent independent dissection studies show that it consists of the inferior-fronto-occipital fascicle, the inferior and middle longitudinal fascicle, optic radiation, the PTR and anterior commissure, which in some areas of the SagS cross each other [44, 45]. The previously observed lower FA in OCD by Piras and colleagues [2] may therefore be due to a lower directionality of the dominant diffusion direction caused by a higher incidence of crossing fibers. Crossing fibers similarly affects MD and other tensor measures [46]. Unfortunately, our current fixel-based analyses cannot disentangle which specific fiber population (e.g. inferior longitudinal fascicle, optic radiation, etc.) showed a higher fiber density as this would require a different approach with individualized tract segmentation (e.g. using TractSeg [47]) which was beyond the scope of this paper.

Compared with early onset cases, individuals with late onset OCD also exhibited a lower global efficiency, indicative of a reduced ability of the brain network to integrate information from different regions. As the capacity to integrate information relies on the integrity of long-range associative fibers, this finding aligns with our results on the PTR and the widespread lower ND in right hemispheric white matter. We did not observe any topological differences when comparing all OCD cases with HC, although our post-hoc analyses suggested that relative to HC, the early onset and late onset OCD group exhibited a higher and lower global efficiency, respectively, albeit not statistically significant. Previous case-control studies on the structural connectome have shown similar [11, 48], higher [13], and lower [12] global efficiency in OCD cases compared with HC. These discrepancies across studies have previously been suggested to be due to differences in age of onset (ranging from 14-25 years across these studies) [13], but no associations with age of onset have previously been reported. The current study suggests that, similar to the observed changes in microstructure, that age of onset impacts the global topology of the structural connectome. Nevertheless, additional validation is necessary to confirm these findings.

This study has a number of strengths. Firstly, the diverse and large sample size, second only to the study of the ENIGMA OCD consortium, and the use of deep phenotyping, inclusion of medication-free individuals, harmonized acquisition protocols, and the use of multi-shell dMRI (none of which the ENIGMA OCD consortium could do due to the use of legacy data). Secondly, the multi-shell dMRI allowed us to use several dMRI methods to approach the data from different angles and two statistical approaches to make inferences. To this day, NHST is the prevailing statistical method of choice and reporting those outcomes makes our results more comparable with previous literature. Nevertheless, this framework has repeatedly been criticized in neuroscientific research (and beyond) for overemphasizing and misinterpreting the *p*-values, the ‘pass’/’fail’ dichotomization that comes with it and its contribution to publication bias and the replication crisis [34, 49, 50]. In the context of neuroimaging, Bayesian Multilevel Modeling has several advantages over NHST: rather than fitting separate models for each region of interest under the assumption of independence, the BHT framework builds one integrative model across all regions that embraces their interrelations - a more rational strategy given that they are derived from the same brain – that also eliminates the need to perform multiple comparison corrections [34, 51]. Furthermore, this framework is more respectful of the continuous nature of biological measures, better controls magnitude and sign errors, and stimulates full reporting of the results thereby improving transparency and reproducibility [50, 51]. The use of integrative models (both the multivariate mixed and multilevel Bayesian models) also prevents the isolated interpretation of tensor-based measures that by themselves do not reliably capture the underlying biophysics of the white matter microstructure [52].

A number of limitations also deserve mentioning. Although the results were consistent overall, the use of multiple different (complementary) approaches also increased the risk of contrasting findings: there were some differences in the outcomes between the NHST and BHT analyses and between the two different site correction procedures. This seems mainly due to the need to perform multiple comparison corrections under the NHST framework and the pass/fail dichotomization in reporting as all reported results showed similar signs and magnitudes and with the exception of the SSRI/SNRI analyses, the confidence intervals of the ComBat and random intercept mixed models were similar. Cases and controls showed significant differences in years of education and IQ but with a mean difference of less than 1 year of education and 2.5 IQ points. Although statistically significant, these differences are likely too small to be of clinical relevance. Similarly, the difference in age (3 years) and IQ (4 points) between early and late-onset OCD cases had negligible influence on the reported results and all models were adjusted for inter-individual differences in age, sex and education level. Adjusting the models for IQ rather than education level had little influence on the results (data not shown).

In conclusion, the results of this OCD global study reveal a notable reduction in microstructural integrity of the white matter in late-onset OCD cases, particularly in thalamo-parietal/occipital tracts, concomitant with reduced efficiency of the structural connectome. These results lend further support for the role of the thalamus and afferent fibers and visual attentional processes in the pathophysiology of OCD.

### Supplementary information

supplementary material

## Data Availability

Raw imaging and clinical data will be made available in the NIMH Data Archive. Processing scripts are available from the author’s github page: github.com/chrisvriend/dwi-prep4tract, github.com/chrisvriend/dwi-dtitk, github.com/chrisvriend/dwi-fba
