# Supplementary material for: Age of onset of obsessive-compulsive disorder differentially affects white matter microstructure
Source: Mol Psychiatry. 2024 Jan 16;29(4):1033–45. doi: 10.1038/s41380-023-02390-8 (PMC11176057; doi:10.1038/s41380-023-02390-8)
Supplement: Supplementary file 1 — supplementary material [file 41380_2023_2390_MOESM1_ESM.docx]

**SUPPLEMENTARY MATERIALS**

**SUPPLEMENTARY METHODS**

**Participants and measures**

Exclusion criteria for individuals with OCD were a lifetime diagnosis of psychosis, bipolar disorder, anorexia, autism or Tourette disorder or a current diagnosis of a chronic tic, substance-use or binge-eating disorder, bulimia or suicidality, and current use of psychotropic medication or cognitive behavioral therapy for OCD. For HC, exclusion criteria were any current or lifetime psychiatric disorder except major depressive or anxiety disorders before the previous year, use of psychotropic medication or having a first-degree relative with OCD or tic disorder. Other exclusion criteria for all participants were any major medical or neurological disease, an IQ < 80 or contraindications to MRI (e.g. metal implants).

All participants underwent a standardized assessment of IQ but because there are no globally available IQ tests, each site performed their own validated IQ that was available in their language: Brazil; Wechsler Abbreviated Scale of Intelligence First Edition (WASI-I); India: Binet Kamat Test; Netherlands: selected subscales from the Wechsler Adult Intelligence Scale Fourth Edition (WAIS-IV); South Africa: WASI Second Edition (WASI-II); USA: WASI-II). The main text reports the clinical measures relevant for the current study. Supplementary Table 31 shows the full list of clinical measures that were administered.

**MRI acquisition and preprocessing**

We acquired multi-shell dMRI with 73 diffusion-weighted directions (25 b1000, 24 b2000, and 24 b3000 s/mm^2^) and seven interleaved non-diffusion-weighted volumes (b0 s/mm^2^) and 3D T1-weighted structural images that included correction of 3D geometric distortion and intensity non-uniformity. Additional blip-up/blip-down scans were acquired with opposite phase-encoding directions to estimate a map for distortion correction. These MRI sequences were harmonized across the five MRI scanners. See supplementary Table 1 for the spatial and timing parameters and our previous work for details on the study MRI protocol and its harmonization.(Pouwels et al. 2023) Diffusion images were denoised using the *dwidenoise* tool in MRtrix3,(Tournier et al. 2019) and subsequently corrected for eddy currents, susceptibility-induced distortions and motion, both within and between volumes using eddy from FMRIB Software Library (FSL; v6.0.5). We used eddyqc(Bastiani et al. 2019) to extract image quality measures (IQMs). Outliers on IQMs were subjected to further visual inspection to identify volumes with residual (motion-related) artifacts and deleted if necessary. Scans were excluded in case of >3 volumes per shell with motion artifacts. IQMs were also compared between groups (see data analysis).

**Fixel measures**

A fixel (i.e., fiber population within a voxel) analysis calculates fiber bundle-specific measures such as the fiber density (FD), fiber cross-section (FC) and fiber density and cross-section (FDC)(Raffelt et al. 2017) to overcome the problem of crossing fibers that are present in approximately 90% of all brain voxels.(Jeurissen et al. 2013) For this we estimated the fiber orientation distributions (FOD) from the full multi-shell dMRI data of each participant using multi-tissue spherical deconvolution (msmt_csd) after upsampling the data to 1.25 mm voxels and calculating averaged three tissue (white matter, grey matter, and cerebrospinal fluid) response functions across the whole sample. We subsequently created an unbiased FOD template from a random representative sample of 125 participants (25 per site). See Supplementary Table 32 for the demographic and clinical characteristics of this subsample. All data were thereafter registered and warped to the template and we extracted the median FD, FDC and log-transformed FC (logFC) for all aforementioned (bilateral) tracts after non-linearly registering the JHU-ICBM atlas to the group-template in ‘fixel-space’. A step-by-step breakdown of this pipeline is provided in the developer’s manual (mrtrix.readthedocs.io)

**Tractography and network analysis**

A gray/white matter boundary was constructed after generating a five-tissue-type (5TT) segmentation based on Hybrid Surface and Volume segmentation (HSVS) using existing FreeSurfer output (v7.1.1). We calculated a number of different global and nodal graph measures:

*Global:* global efficiency, modularity, small-worldness, and rich club coefficient. Global efficiency represents how efficiently information can be transferred through the network. Modularity measures represent how divided the network is into interconnected groups of nodes (i.e., modules) with a higher modularity indicating a more modular structure. Small-worldness represents the ratio between local clustering and global integration. A healthy network balances these two properties for efficient information transfer. Lastly, the rich club coefficient indicates the presence of a set of highly interconnected nodes (i.e. hubs) that form an influential backbone of the network and has been shown to be altered in OCD (Baldi et al. 2022). The normalized rich club coefficient (theta) was calculated according to (Alstott et al. 2014). Our pre-registration stated that we would calculate the normalized rich club coefficient for a range of cut-offs of the most strongly connected nodes where theta>1 across all participants. We discovered, however, that there was no cut-off for which theta>1 across all participants. We therefore calculated the normalized theta for the cut-off at which most participants had theta>1; a cut-off of 12 nodes.

*Nodal:* Betweenness centrality and local efficiency. Betweenness centrality represents the number of shortest paths that pass through a node to connect any two other nodes and represents the influence of that node on global network connectivity. Local efficiency indicates how well a node facilitates and contributes to communication among its immediate neighbors.

**SUPPLEMENTARY FIGURES**


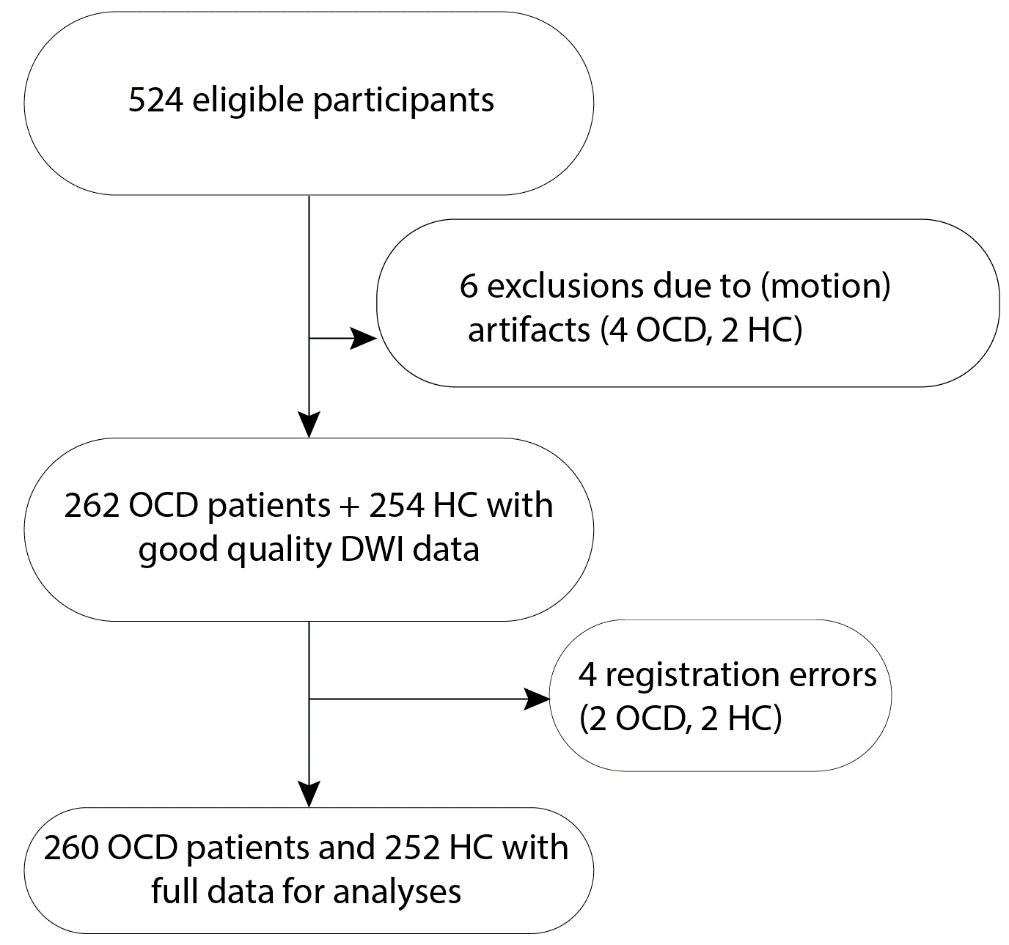


**Supplementary Figure 1 - Flowchart**


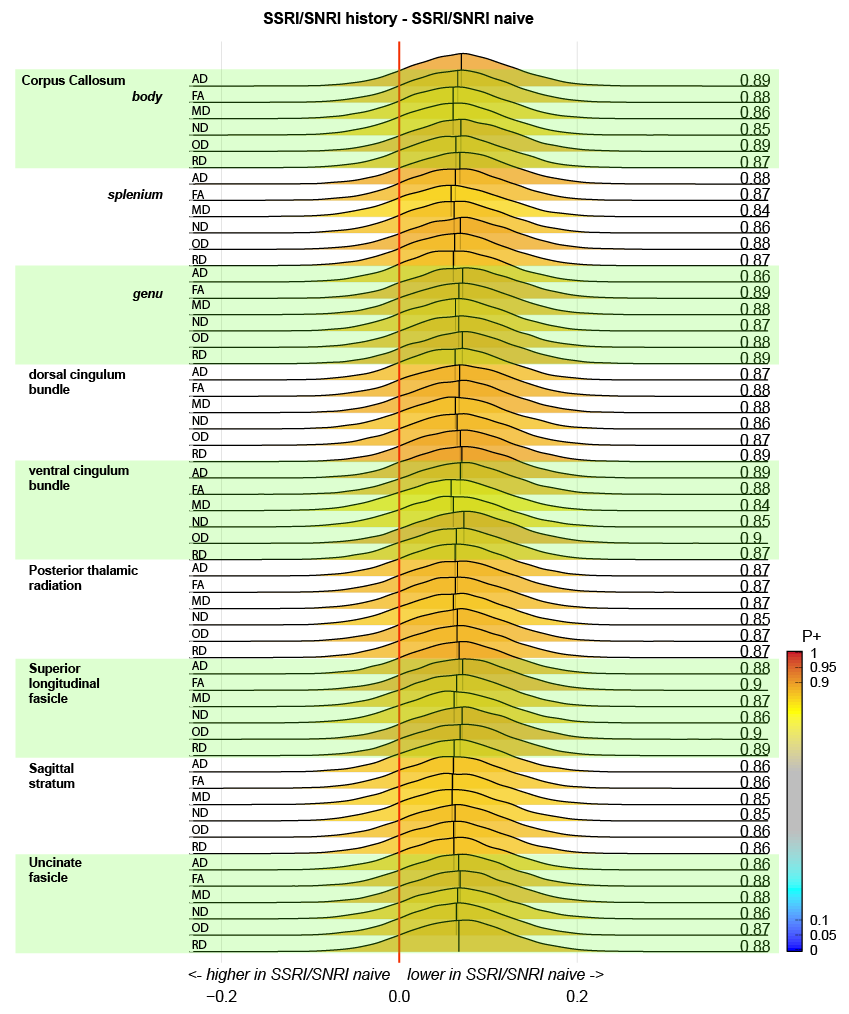


**Supplementary Figure 2** – **Bayesian posterior distribution plots of the differences in white matter microstructure between individuals with OCD that do or do not have a history of serotonin/noradrenaline reuptake inhibitors (SSRI/SNRI) use.** Posterior probabilities of a positive effect (P+) are shown next to each distribution and color coded. The meaning of the direction of effects are shown next to the red zero-effect line. Values on the X-axis represent (inverted) Z-scores (i.e. the unit to which the tensor/NODDI measures were converted; see methods section). Across the regions of interest the probability distributions show evidence for lower integrity of the white matter in SSRI/SNRI naive individuals although none of the P+ values ≥0.90. Abbreviations: AD: axial diffusivity, FA: fractional anisotropy, MD: mean diffusivity, ND: neurite density, OD: orientation dispersion, RD: radial diffusivity, NODDI: Neurite orientation dispersion and density imaging. Plots were produced using the Region-Based Analysis program through Bayesian Multilevel Modeling implemented in AFNI (Chen et al. 2019)

**
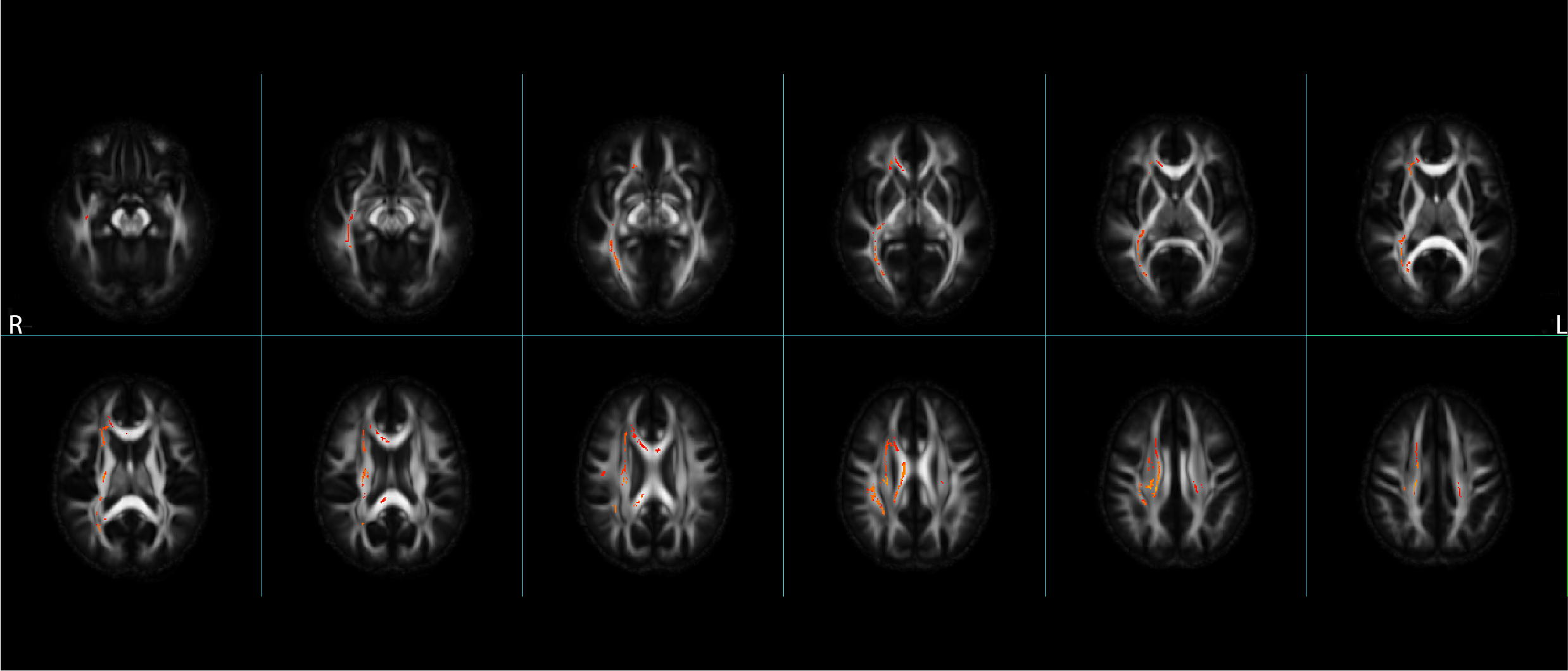
**

**Supplementary Figure 3 - Lower neurite density in late onset compared with early onset OCD patients.**

Tract-based spatial statistics neurite density (ND) map limited to voxels of the skeletonized white matter voxels of the JHU-ICBM-DTI-81 atlas. Results are shown at threshold free cluster enhancement (TFCE) *P_FWE_*<0.05 and adjusted for age, sex, years of education and site.


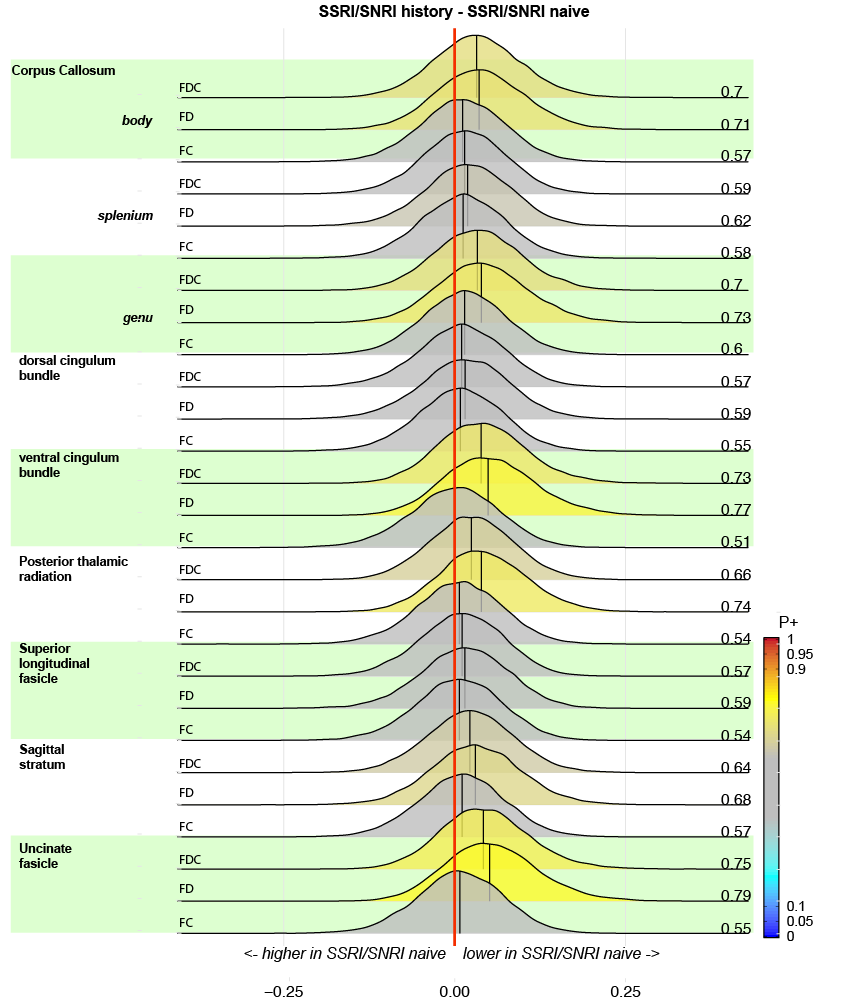


**Supplementary Figure 4** – **Bayesian posterior distribution plots on fixel-based measures between individuals with OCD that do or do not have a history of selective serotonin/noradrenaline reuptake inhibitors (SSRI/SNRI) use.** The posterior distributions show no credible evidence for a difference in any of the fixel measures. Posterior probabilities of a positive effect (P+) are shown next to each distribution and color coded. abbreviations: FDC: fiber density and cross-section, FD: fiber density, FC: fiber cross section.


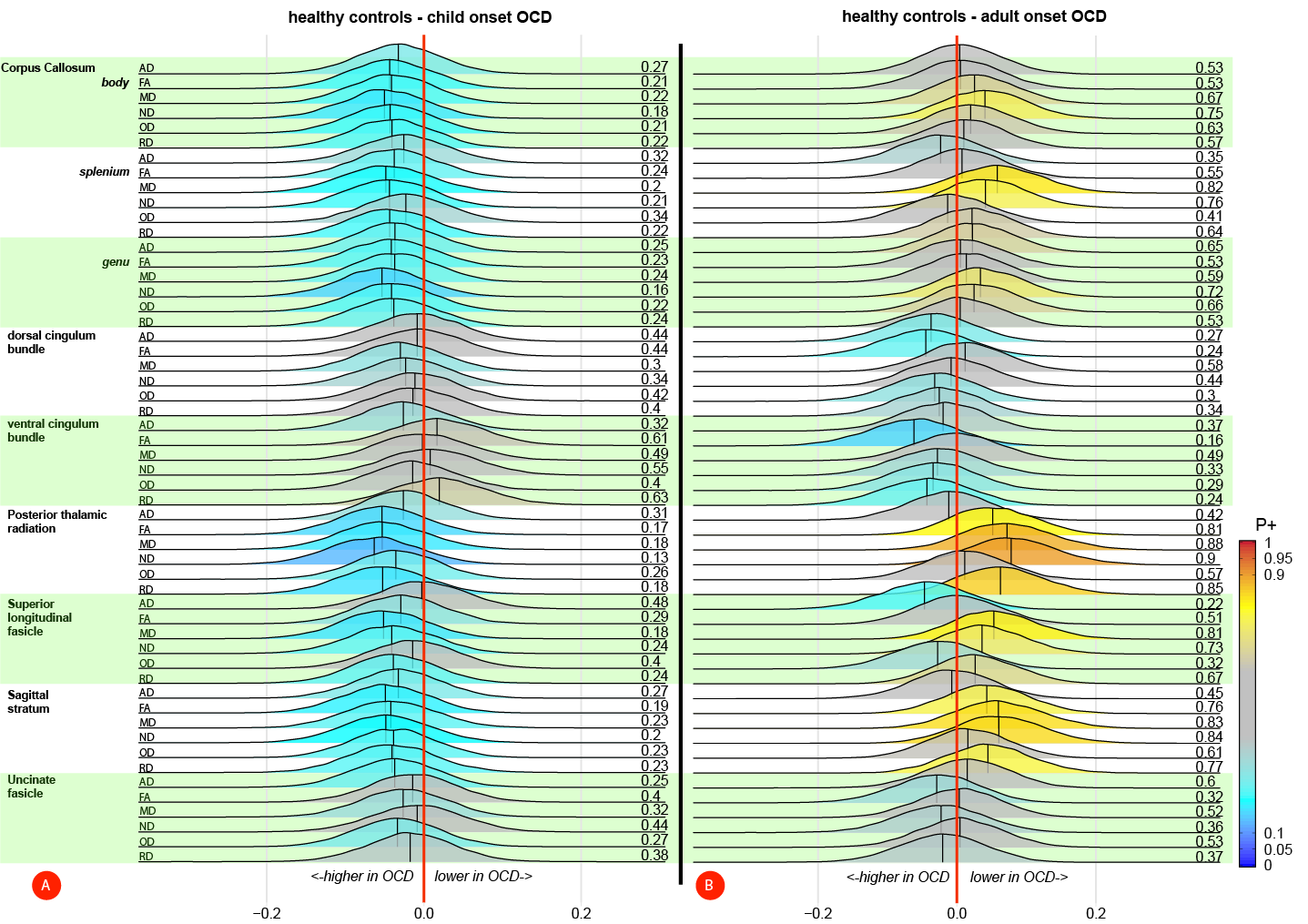


**Supplementary Figure 5** – **Bayesian posterior distribution plots on tensor/NODDI measures between individuals with healthy controls and (A) early onset or (B) late onset OCD.** Posterior probabilities of a positive effect (P+) are shown next to each distribution and color coded. The meaning of the direction of effects are shown next to the red zero-effect line. Values on the X-axis represent (inverted) Z-scores (i.e. the unit to which the tensor/NODDI measures were converted; see methods section). Across the regions of interest, particularly the posterior thalamic radiation, superior longitudinal fascicle and sagittal stratum, the probability distributions show evidence for higher integrity of the white matter in individuals with early onset OCD (a), while individuals with late onset OCD (b) show the opposite pattern. Abbreviations: AD: axial diffusivity, FA: fractional anisotropy, MD: mean diffusivity, ND: neurite density, OD: orientation dispersion, RD: radial diffusivity, NODDI: Neurite orientation dispersion and density imaging. Plots were produced using the Region-Based Analysis program through Bayesian Multilevel Modeling implemented in AFNI (Chen et al. 2019).


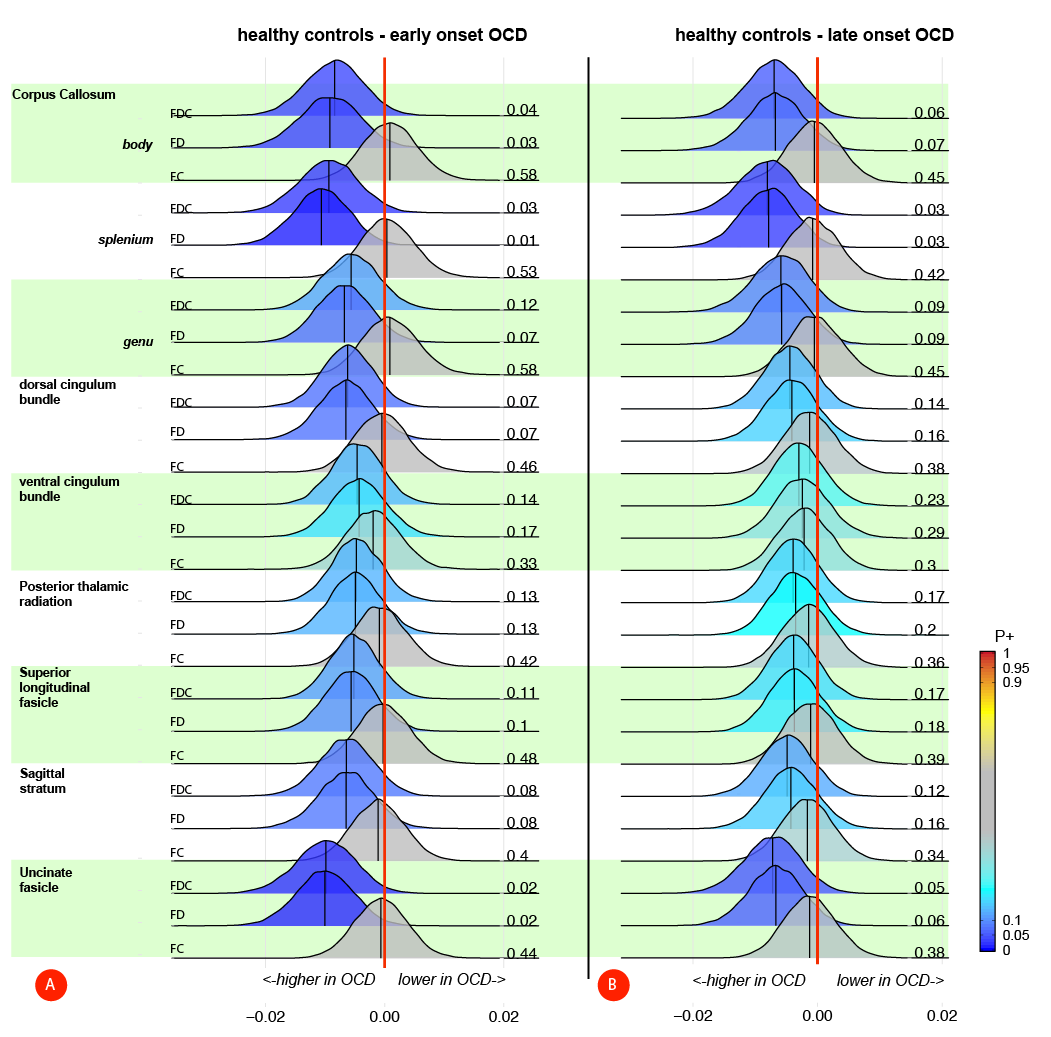


**Supplementary Figure 6** – **Bayesian posterior distribution plots on fixel measures between individuals with healthy controls and (A) early onset or (B) late onset OCD.** Posterior probabilities of a positive effect (P+) are shown next to each distribution and color coded. The meaning of the direction of effects are shown next to the red zero-effect line. Unlike the tensor/NODDI measures, these analyses show evidence for a higher fiber density (FD) and fiber density & cross-section (FDC) in individuals with either early or late onset OCD relative to healthy controls (no credible evidence for differences in fiber cross-section).


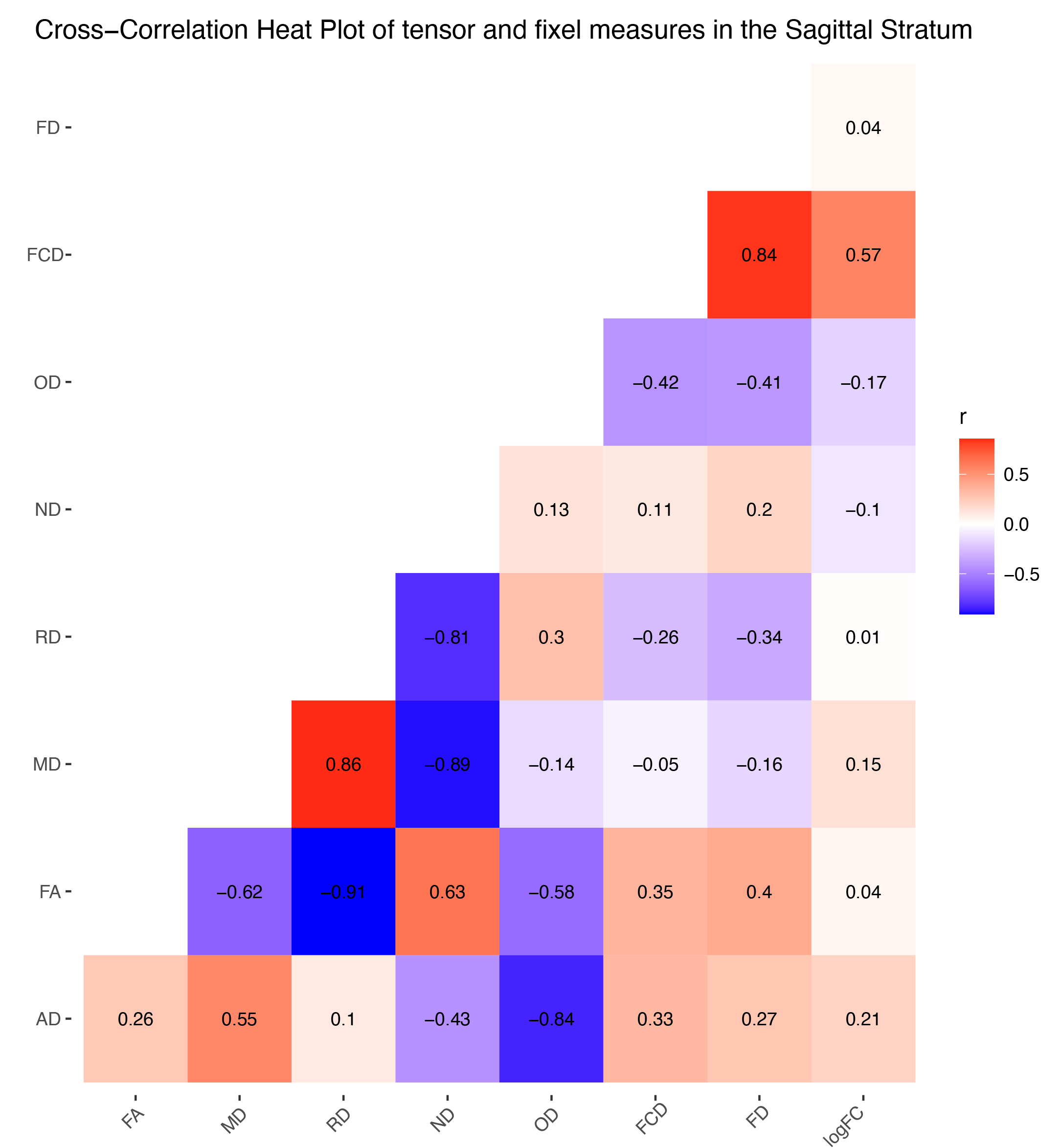


**Supplementary Figure 7** - correlation heat map of NODDI/tensor and fixel based measures. Apparent from this plot is that correlations are relatively low between tensor and fixel based measures, although the correlations between the multi-shell NODDI and fixel-based measures were relatively higher. The Sagittal stratum was chosen here, although the same pattern was seen for the other tracts of interest.

**SUPPLEMENTARY TABLES**

| **Supplementary Table 1 – Imaging parameters** | | | | | |
| --- | --- | --- | --- | --- | --- |
| **site** | **1** | **2** | **3** | **4** | **5** |
| **Scanner** | Philips Achieva 3.0T | Philips Ingenia 3.0T CX | GE 3.0T Discovery MR750 | Siemens MAGNETOM Skyra 3.0T | GE SIGNA 3.0T Premier |
| **Head coil** | 32-channel | 32-channel | 32-channel | 32-channel | 48-channel |
| **3D sagittal T1-weighted MPRAGE** | | | | | |
| **TR (ms) ^a^** | 6.5 | 6.5 | 6.9 | 2300 | 2235 |
| **TI (ms)** | 900 | | | | |
| **TE (ms)** | 2.9 | 2.9 | 3 | 2 | 2.8 |
| **Flip angle (⁰)** | 9 | | | | |
| **Voxel size (mm)** | 1 x 1 x 1 | | | | |
| **Matrix** | 256 x 256 | | | | |
| **dMRI** | | | | | |
| **TR (ms)** | 7220 | 7220 | 6310 | 7300 | 7000 |
| **TE (ms)** | 100 | 100 | 81 | 100 | 74 |
| **Flip angle (⁰)** | 90 | | | | |
| **# slices** | 56 | | | | |
| **Voxel size (mm)** | 2.5 x 2.5 x 2.5 | | | | |
| **Matrix** | 96 x 96 | | | | |

Footnote: a) values for TR are highly variable due to different definitions of TR for this pulse sequence. Abbreviations: TR = repetition time, TE = echo time, TI = inversion time, T1w = T1 weighted, MPRAGE = magnetization-prepared rapid acquisition gradient-echo, dMRI = diffusion weighted magnetic resonance imaging

| **Supplementary Table 2 – Demographic and clinical characteristics of age of onset and medication groups** | | | | | | |
| --- | --- | --- | --- | --- | --- | --- |
|  | **Early (N=145)**  M (SD) | **Late (n=114)**  M (SD) | **Statistics** | **SSRI/SNRI naïve (N=149)**  M (SD) | **SSRI/SNRI history (N=111)** M (SD) | **Statistics** |
| **Sex (N (%))** |  |  |  |  |  |  |
| **Male** | 71 (49.0%) | 46 (40.4%) | χ^2^_(1)_= 1.9, P = 0.21 | 67 (45.0%) | 50 (45.0%) | χ^2^_(1)_< 0.001, P = 0.99 |
| **Female** | 74 (51.0%) | 68 (59.6%) |  | 82 (55.0%) | 61 (55.0) |  |
| **Age (years)** | 28.2 (7.9) | 31.2 (7.8) | t_(257)_= -3.07, P =0.002 | 28.8 (7.8) | 30.5 (8.0) | t(258) = -1.7, P=0.08 |
| **Age at onset (years)** | 12.4 (3.3) | 23.4 (5.6) | _t(172,9)_=-18.6, P<0.001 | 17.2 (7.3) | 17.2 (6.9) | t(257) = 0.09, P=0.93 |
| **Education (years)** | 15.2 (2.9) | 15.3 (2.7) | t_(257)_= -0.26, P = 0.79 | 15.3 (2.7) | 15.2 (2.9) | t(258) = 0.27, P=0.79 |
| **IQ** | 106.8 (12.1) | 102.0 (12.4) | t_(257)_= 3.18, P = 0.002 | 105.5 (12.5) | 103.8 (12.2) | t(258) = 1.10, P=0.27 |
| **YBOCS** | 24.6 (4.6) | 25.0 (5.4) | t_(219,9)_= -0.50, P = 0.61 | 24.2 (4.8) | 25.6 (5.1) | t(258) = -2.13, P=0.03 |
| **Duration of illness** | 15.8 (8.5) | 7.8 (6.2) | t_(255,9)_= 8.5, P < 0.001 | 11.5 (8.7) | 13.3 (8.3) | t(258) = -1.70, P=0.09 |
| **Medication naïve (% of sample)** |  |  |  |  |  |  |
| SSRI | 56.6% | 60.5% | χ^2^_(1)_= 0.42, P = 0.52 | 100% | 3% | χ^2^_(1)_= 248, P < 0.001 |
| SNRI | 94.5% | 96.5% | χ^2^_(1)_= 0.58, P = 0.45 | 100% | 89% | χ^2^_(1)_= 16.9, P < 0.001 |
| Benzodiazepines | 89.0% | 92.1% | χ^2^_(1)_= 0.72, P = 0.40 | 96.6% | 82.0% | χ^2^_(1)_= 15.7, P < 0.001 |
| Antipsychotics | 88.3% | 96.5% | χ^2^_(1)_= 5.8, P = 0.02 | 98.6% | 82,8% | χ^2^_(1)_= 21.3, P < 0.001 |
| Mood stabilizers | 95.9% | 99.1% | χ^2^_(1)_= 2.6, P = 0.11 | 98.6% |  |  |
| **CBT naïve** | 70.3% | 82.5% | χ^2^_(1)_= 5.1, P = 0.02 | 81.9% | 67.5% | χ^2^_(1)_= 7.1, P = 0.008 |
| **Comorbidities (% of sample)** |  |  |  |  |  |  |
| Tourette | - | - |  |  |  |  |
| ADHD – current | 8.3% | 5.3% | χ^2^_(1)_= 0.90, P = 0.34 | 5.3% | 9.0% | χ^2^_(1)_= 1.3, P = 0.25 |
| ADHD - lifetime | 11.0% | 5.3% | χ^2^_(1)_= 2.74, P = 0.10 | 6.0% | 11.7% | χ^2^_(1)_= 2.6, P = 0.10 |
| MDD - current | 24.1% | 20.2% | χ^2^_(1)_= 0.58, P = 0.45 | 17.4% | 28.9% | χ^2^_(1)_= 4.7, P = 0.03 |
| Dysthymia – current | 6.2% | 4.4% | χ^2^_(1)_= 0.41, P = 0.52 | 3.4% | 5.4% | χ^2^_(1)_= 2.8, P = 0.09 |
| MDD – lifetime | 51.7% | 42.1% | χ^2^_(1)_= 2.4, P = 0.12 | 45.0% | 51.3% | χ^2^_(1)_= 1.04, P = 0.31 |
| PTSD – current | 2.8% | 2.6% | χ^2^_(1)_= 0.004, P = 0.95 | 2.6% | 2.7% | χ^2^_(1)_< 0.001, P = 0.99 |
| PTST – lifetime | 6.9% | 6.1% | χ^2^_(1)_= 0.06, P = 0.81 | 4.0% | 9.9% | χ^2^_(1)_= 3.6, P = 0.06 |
| Panic disorder – current | 9.0% | 6.9% | χ^2^_(1)_= 2.1, P = 0.15 | 4.0% | 6.9% | χ^2^_(1)_= 4.5, P = 0.03 |
| Panic disorder – lifetime | 14.5% | 7.9% | χ^2^_(1)_= 2.7, P = 0.10 | 7.4% | 11.5% | χ^2^_(1)_= 5.9, P = 0.02 |
| Agoraphobia – current | 4.1% | 5.0% | χ^2^_(1)_= 0.54, P = 0.46 | 6.0% | 3.6% | χ^2^_(1)_= 0.80, P = 0.37 |
| Agoraphobia – lifetime | 5.5% | 6.1% | χ^2^_(1)_= 0.05, P = 0.83 | 6.0% | 5.4% | χ^2^_(1)_= 0.05, P = 0.83 |
| Social Anxiety disorder – current | 20.0% | 17.5% | χ^2^_(1)_= 0.25, P = 0.61 | 16.8% | 22.5% | χ^2^_(1)_= 1.4, P = 0.25 |
| Social Anxiety disorder – lifetime | 25.5% | 20.2% | χ^2^_(1)_= 1.0, P = 0.31 | 23.5% | 23.5% | χ^2^_(1)_< 0.001, P = 0.99 |
| Specific phobia – current | 18.6% | 15.1% | χ^2^_(1)_= 3.3, P = 0.07 | 14.1% | 16.2% | χ^2^_(1)_= 0.23, P = 0.64 |
| Specific phobia – lifetime | 19.3% | 12.3% | χ^2^_(1)_= 2.3, P = 0.13 | 15.4% | 17.1% | χ^2^_(1)_= 0.13, P = 0.72 |
| Generalized Anxiety Disorder – current | 27.6% | 22.8% | χ^2^_(1)_= 0.77, P = 0.38 | 19.5% | 25.8% | χ^2^_(1)_= 7.3, P = 0.007 |
| Generalized Anxiety Disorder – lifetime | 28.3% | 21.9% | χ^2^_(1)_= 1.3, P = 0.25 | 18.8% | 35.1% | χ^2^_(1)_= 8.9, P = 0.003 |
| Hoarding – current | 6.2% | 5.4% | χ^2^_(1)_= 0.41, P = 0.52 | 2.0% | 9.9% | χ^2^_(1)_= 7.8, P = 0.005 |
| Hoarding – lifetime | 6.2% | 4.6% | χ^2^_(1)_= 1.8, P = 0.17 | 2.0% | 8.1% | χ^2^_(1)_= 5.4, P = 0.02 |
| Body Dysmorphic disorder – current | 6.2% | 4.4% | χ^2^_(1)_= 0.41, P = 0.52 | 2.0% | 9.9% | χ^2^_(1)_= 7.8, P = 0.005 |
| Body Dysmorphic disorder – lifetime | 8.3% | 7.7% | χ^2^_(1)_= 0.14, P = 0.71 | 5.4% | 10.8% | χ^2^_(1)_= 2.7, P = 0.10 |
| Substance use disorder - current | - | - |  | - | - |  |
| Substance use disorder – lifetime | 2.1% | 5.3% | χ^2^_(1)_= 1.9, P = 0.16 | 2.7% | 4.5% | χ^2^_(1)_= 0.63, P = 0.43 |
| Schizophrenia | - | - |  | - | - |  |
| Bipolar disorder I | 0.7% | - | χ^2^_(1)_= 0.79, P = 0.37 | 0.6% | - | χ^2^_(1)_= 0.75, P = 0.39 |
| Bipolar disorder II | - | - |  | - | - |  |
| **DYBOCS** |  |  |  |  |  |  |
| Harm & Aggression | 5.4 (4.7) | 5.3 (4.7) | U=7987.5, P=0.70 | 5.2 (4.5) | 5.6 (5.0) | U=7803, P=0.50 |
| Sexual & Religious | 4.6 (4.8) | 4.3 (5.0) | U=7979, P=0.76 | 4.3 (4.7) | 4.7 (5.2) | U=7849, P=0.62 |
| Symmetry & Ordering | 6.1 (4.4) | 5.2 (4.4) | U=7202, P=0.09 | 5.5 (4.4) | 6.1 (4.5) | U=7454, P=0.21 |
| Contamination | 6.3 (4.9) | 6.4 (5.2) | U=7975, P=0.76 | 6.0 (4.8) | 6.7 (5.2) | U=7396, P=0.21 |
| Collecting & Hoarding | 1.4 (2.7) | 1.2 (2.6) | U=7469.5, P=0.20 | 1.1 (2.5) | 1.6 (2.9) | U=7569, P=0.37 |
| Data are presented as mean (SD) unless otherwise indicated. Abbreviations: YBOCS = Yale-Brown Obsessive-compulsive Scale, SSRI = selective serotonin reuptake inhibitor, SNRI = selective noradrenaline reuptake inhibitor, CBT = Cognitive behavioral therapy. ADHD = attention deficit hyperactivity disorder, MDD = major depressive disorder, PTSD = post-traumatic stress disorder , DYBOCS = dimensional YBOCS. | | | | | | |

| **Supplementary Table 3 – Between-group differences in Image Quality Measures** | | | | | |
| --- | --- | --- | --- | --- | --- |
|  | **HC** | **OCD** | **Group difference** | | |
|  | M ± SD | M ± SD | B [SE] | 95% CI | P |
| **CNR** | | | | | |
| b0 | 36.49 ± 8.034 | 35.606 ± 7.799 | -88.400 [<0.001] | -225.8 \| 49.1 | 0.207 |
| b1000 | 2.837 ± 1.059 | 2.812 ± 1.102 | -2.500 [9.600] | -21.3 \| 16.3 | 0.795 |
| b2000 | 5.765 ± 3.11 | 5.872 ± 3.137 | 10.800 [27.600] | -43.5 \| 65 | 0.697 |
| b3000 | 4.221 ± 1.793 | 4.258 ± 1.851 | 3.600 [16.100] | -28 \| 35.3 | 0.821 |
| **Percentage outliers** | | | | | |
| b1000 | 1.399 ± 0.87 | 1.466 ± 0.933 | 6.700 [8.000] | -9 \| 22.4 | 0.400 |
| b2000 | 0.089 ± 0.176 | 0.12 ± 0.224 | 3.100 [1.800] | -0.4 \| 6.6 | 0.081 |
| b3000 | 0.099 ± 0.162 | 0.132 ± 0.222 | 3.200 [1.700] | -0.1 \| 6.6 | 0.060 |
| **Framewise displacement** | | | | | |
| relative | 0.432 ± 0.215 | 0.447 ± 0.227 | 1.500 [2.000] | -2.3 \| 5.4 | 0.432 |
|  | | | | | |
|  | **Late OCD onset** | **Early OCD onset** | **Group difference** | | |
|  | M ± SD | M ± SD | B [SE] | 95% CI | P |
| **CNR** | | | | | |
| b0 | 35.784 ± 7.999 | 35.445 ± 7.685 | -33.900 [97.900] | -226.8 \| 158.9 | 0.729 |
| b1000 | 2.931 ± 1.179 | 2.713 ± 1.033 | -21.800 [13.800] | -48.9 \| 5.3 | 0.114 |
| b2000 | 6.148 ± 3.271 | 5.652 ± 3.032 | -49.600 [39.300] | -127 \| 27.8 | 0.208 |
| b3000 | 4.339 ± 1.856 | 4.183 ± 1.852 | -15.600 [23.200] | -61.3 \| 30.1 | 0.503 |
| **Percentage outliers** | | | | | |
| b1000 | 1.509 ± 0.964 | 1.435 ± 0.912 | -7.500 [11.700] | -30.5 \| 15.6 | 0.523 |
| b2000 | 0.097 ± 0.191 | 0.137 ± 0.247 | 4.000 [2.800] | -1.5 \| 9.6 | 0.153 |
| b3000 | 0.126 ± 0.208 | 0.135 ± 0.232 | 0.900 [2.800] | -4.6 \| 6.4 | 0.747 |
| **Framewise displacement** | | | | | |
| relative | 0.441 ± 0.219 | 0.453 ± 0.234 | 1.200 [2.900] | -4.4 \| 6.9 | 0.664 |
|  | | | | | |
|  | **Naïve for SSRI/SNRI** | **Not Naïve for SSRI/SNRI** | **Group difference** | | |
|  | M ± SD | M ± SD | B [SE] | 95% CI | P |
| **CNR** | | | | | |
| b0 | 35.804 ± 7.893 | 35.34 ± 7.697 | 46.400 [97.900] | -146.4 \| 239.3 | 0.636 |
| b1000 | 2.821 ± 1.165 | 2.8 ± 1.017 | 2.100 [13.800] | -25.1 \| 29.4 | 0.879 |
| b2000 | 5.802 ± 3.281 | 5.966 ± 2.945 | -16.400 [39.400] | -94 \| 61.2 | 0.678 |
| b3000 | 4.229 ± 1.944 | 4.296 ± 1.725 | -6.800 [23.200] | -52.6 \| 39 | 0.771 |
| **Percentage outliers** | | | | | |
| b1000 | 1.422 ± 0.961 | 1.524 ± 0.895 | -10.200 [11.700] | -33.2 \| 12.8 | 0.384 |
| b2000 | 0.11 ± 0.202 | 0.133 ± 0.252 | -2.200 [2.800] | -7.8 \| 3.3 | 0.428 |
| b3000 | 0.118 ± 0.184 | 0.15 ± 0.264 | -3.200 [2.800] | -8.7 \| 2.3 | 0.253 |
| **Framewise displacement** | | | | | |
| relative | 0.45 ± 0.236 | 0.444 ± 0.216 | 0.600 [2.900] | -5.1 \| 6.2 | 0.847 |
| Abbreviations: HC = healthy control, OCD = obsessive-compulsive disorder, SSRI/SNRI = selective serotonin or serotonin noradrenaline reuptake inhibitors, CNR = contrast-to-noise ratio | | | | | |

| **Supplementary Table 4 – Case-control mixed model analyses of white matter microstructure – random effect for site** | | | | | | |
| --- | --- | --- | --- | --- | --- | --- |
|  | **Group difference (crude model)** | | | **Group difference (adjusted model)*** | | |
|  | B [SE] | 95% CI | P (unc) | B [SE] | 95% CI | P (unc) |
| **CC genu** | | | | | | |
| Overall diffusion | 0.038 [0.040] | -0.05 \| 0.13 | 0.876 (0.400) | 0.019 [0.040] | -0.07 \| 0.1 | 0.897 (0.659) |
| AD | 0.019 [0.090] | -0.15 \| 0.19 | 0.827 (0.827) | <0.001 [0.090] | -0.17 \| 0.17 | 1.000 (1.000) |
| FA | 0.067 [0.090] | -0.1 \| 0.23 | 0.820 (0.438) | 0.048 [0.090] | -0.12 \| 0.21 | 0.883 (0.573) |
| MD | 0.034 [0.090] | -0.13 \| 0.2 | 0.815 (0.689) | 0.016 [0.090] | -0.15 \| 0.18 | 0.961 (0.854) |
| RD | 0.052 [0.090] | -0.12 \| 0.22 | 0.705 (0.548) | 0.033 [0.090] | -0.13 \| 0.2 | 0.860 (0.700) |
| ND | 0.103 [0.090] | -0.07 \| 0.27 | 0.920 (0.232) | 0.084 [0.090] | -0.08 \| 0.25 | 0.962 (0.324) |
| OD | -0.046 [0.090] | -0.21 \| 0.12 | 0.996 (0.591) | -0.065 [0.090] | -0.23 \| 0.10 | 0.890 (0.446) |
| **CC body** | | | | | | |
| Overall diffusion | 0.073 [0.050] | -0.02 \| 0.17 | 0.876 (0.125) | 0.060 [0.050] | -0.03 \| 0.15 | 0.897 (0.207) |
| AD | 0.031 [0.090] | -0.14 \| 0.2 | 0.812 (0.722) | 0.018 [0.090] | -0.15 \| 0.19 | 0.941 (0.836) |
| FA | 0.165 [0.090] | <0.001 \| 0.34 | 0.522 (0.058) | 0.152 [0.090] | -0.02 \| 0.32 | 0.729 (0.081) |
| MD | 0.070 [0.090] | -0.1 \| 0.24 | 0.815 (0.421) | 0.057 [0.090] | -0.11 \| 0.23 | 0.782 (0.511) |
| RD | 0.136 [0.090] | -0.04 \| 0.31 | 0.705 (0.120) | 0.123 [0.090] | -0.05 \| 0.29 | 0.720 (0.159) |
| ND | 0.036 [0.090] | -0.13 \| 0.21 | 0.920 (0.676) | 0.024 [0.090] | -0.15 \| 0.19 | 0.962 (0.787) |
| OD | <0.001 [0.090] | -0.17 \| 0.17 | 0.996 (0.996) | -0.013 [0.090] | -0.18 \| 0.16 | 0.890 (0.879) |
| **CC splenium** | | | | | | |
| Overall diffusion |  |  |  |  |  |  |
| AD | 0.072 [0.090] | -0.1 \| 0.24 | 0.812 (0.411) | 0.060 [0.090] | -0.11 \| 0.23 | 0.930 (0.490) |
| FA | 0.041 [0.090] | -0.13 \| 0.21 | 0.820 (0.638) | 0.029 [0.090] | -0.14 \| 0.20 | 0.883 (0.735) |
| MD | 0.008 [0.090] | -0.16 \| 0.18 | 0.928 (0.928) | -0.004 [0.090] | -0.17 \| 0.17 | 0.965 (0.965) |
| RD | 0.027 [0.090] | -0.14 \| 0.2 | 0.808 (0.757) | 0.015 [0.090] | -0.16 \| 0.18 | 0.860 (0.860) |
| ND | 0.038 [0.090] | -0.13 \| 0.21 | 0.920 (0.667) | 0.026 [0.090] | -0.14 \| 0.2 | 0.962 (0.766) |
| OD | -0.038 [0.090] | -0.21 \| 0.13 | 0.996 (0.660) | -0.050 [0.090] | -0.22 \| 0.12 | 0.890 (0.566) |
| **Dorsal cingulum** | | | | | | |
| Overall diffusion | 0.017 [0.060] | -0.1 \| 0.13 | 0.876 (0.779) | 0.017 [0.060] | -0.1 \| 0.13 | 0.897 (0.765) |
| AD | 0.079 [0.080] | -0.08 \| 0.24 | 0.812 (0.344) | 0.079 [0.080] | -0.08 \| 0.24 | 0.930 (0.334) |
| FA | 0.043 [0.080] | -0.12 \| 0.21 | 0.820 (0.605) | 0.044 [0.080] | -0.12 \| 0.20 | 0.883 (0.595) |
| MD | -0.055 [0.080] | -0.22 \| 0.11 | 0.815 (0.511) | -0.054 [0.080] | -0.22 \| 0.11 | 0.782 (0.509) |
| RD | -0.020 [0.080] | -0.18 \| 0.14 | 0.808 (0.808) | -0.020 [0.080] | -0.18 \| 0.14 | 0.860 (0.810) |
| ND | 0.034 [0.080] | -0.13 \| 0.2 | 0.920 (0.682) | 0.035 [0.080] | -0.13 \| 0.20 | 0.962 (0.672) |
| OD | 0.020 [0.080] | -0.14 \| 0.18 | 0.996 (0.814) | 0.020 [0.080] | -0.14 \| 0.18 | 0.890 (0.806) |
| **Ventral cingulum** | | | | | | |
| Overall diffusion | <0.001 [0.050] | -0.1 \| 0.1 | 0.996 (0.996) | 0.002 [0.050] | -0.09 \| 0.10 | 0.969 (0.969) |
| AD | 0.094 [0.090] | -0.07 \| 0.26 | 0.812 (0.273) | 0.096 [0.090] | -0.07 \| 0.26 | 0.930 (0.257) |
| FA | -0.014 [0.090] | -0.18 \| 0.15 | 0.869 (0.869) | -0.012 [0.090] | -0.18 \| 0.15 | 0.888 (0.888) |
| MD | -0.078 [0.090] | -0.25 \| 0.09 | 0.815 (0.363) | -0.076 [0.090] | -0.24 \| 0.09 | 0.782 (0.371) |
| RD | -0.062 [0.090] | -0.23 \| 0.1 | 0.705 (0.469) | -0.060 [0.090] | -0.22 \| 0.11 | 0.720 (0.480) |
| ND | -0.009 [0.090] | -0.18 \| 0.16 | 0.920 (0.920) | -0.006 [0.090] | -0.17 \| 0.16 | 0.962 (0.939) |
| OD | 0.067 [0.090] | -0.1 \| 0.23 | 0.996 (0.432) | 0.069 [0.090] | -0.1 \| 0.23 | 0.890 (0.412) |
| **Posterior thal rad** | | | | | | |
| Overall diffusion | -0.046 [0.050] | -0.14 \| 0.05 | 0.876 (0.347) | -0.049 [0.050] | -0.14 \| 0.05 | 0.897 (0.312) |
| AD | 0.052 [0.080] | -0.11 \| 0.22 | 0.812 (0.536) | 0.049 [0.080] | -0.11 \| 0.21 | 0.930 (0.558) |
| FA | -0.084 [0.080] | -0.25 \| 0.08 | 0.820 (0.312) | -0.087 [0.080] | -0.25 \| 0.07 | 0.882 (0.294) |
| MD | -0.100 [0.080] | -0.26 \| 0.06 | 0.815 (0.229) | -0.103 [0.080] | -0.27 \| 0.06 | 0.782 (0.214) |
| RD | -0.106 [0.080] | -0.27 \| 0.06 | 0.705 (0.206) | -0.109 [0.080] | -0.27 \| 0.05 | 0.720 (0.192) |
| ND | -0.018 [0.080] | -0.18 \| 0.14 | 0.920 (0.831) | -0.021 [0.080] | -0.18 \| 0.14 | 0.962 (0.803) |
| OD | -0.021 [0.080] | -0.18 \| 0.14 | 0.996 (0.805) | -0.023 [0.080] | -0.19 \| 0.14 | 0.890 (0.777) |
| **Sag Stratum** | | | | | | |
| Overall diffusion | -0.021 [0.050] | -0.13 \| 0.08 | 0.876 (0.700) | -0.016 [0.050] | -0.12 \| 0.09 | 0.897 (0.761) |
| AD | 0.082 [0.080] | -0.08 \| 0.25 | 0.812 (0.331) | 0.086 [0.080] | -0.08 \| 0.25 | 0.930 (0.309) |
| FA | -0.027 [0.080] | -0.19 \| 0.14 | 0.839 (0.746) | -0.023 [0.080] | -0.19 \| 0.14 | 0.883 (0.785) |
| MD | -0.093 [0.080] | -0.26 \| 0.07 | 0.815 (0.268) | -0.089 [0.080] | -0.25 \| 0.08 | 0.782 (0.292) |
| RD | -0.065 [0.080] | -0.23 \| 0.1 | 0.705 (0.443) | -0.060 [0.080] | -0.23 \| 0.10 | 0.720 (0.475) |
| ND | -0.028 [0.080] | -0.19 \| 0.14 | 0.920 (0.736) | -0.024 [0.080] | -0.19 \| 0.14 | 0.962 (0.775) |
| OD | 0.008 [0.080] | -0.16 \| 0.17 | 0.996 (0.928) | 0.012 [0.080] | -0.15 \| 0.18 | 0.890 (0.890) |
| **SLF** | | | | | | |
| Overall diffusion | -0.015 [0.050] | -0.12 \| 0.09 | 0.876 (0.778) | -0.026 [0.050] | -0.13 \| 0.08 | 0.897 (0.628) |
| AD | 0.040 [0.080] | -0.12 \| 0.21 | 0.812 (0.632) | 0.029 [0.080] | -0.14 \| 0.19 | 0.935 (0.727) |
| FA | -0.043 [0.080] | -0.21 \| 0.12 | 0.820 (0.614) | -0.054 [0.080] | -0.22 \| 0.11 | 0.883 (0.526) |
| MD | -0.043 [0.080] | -0.21 \| 0.12 | 0.815 (0.609) | -0.054 [0.080] | -0.22 \| 0.11 | 0.782 (0.521) |
| RD | -0.058 [0.080] | -0.22 \| 0.11 | 0.705 (0.494) | -0.069 [0.080] | -0.23 \| 0.10 | 0.720 (0.416) |
| ND | 0.015 [0.080] | -0.15 \| 0.18 | 0.920 (0.859) | 0.004 [0.080] | -0.16 \| 0.17 | 0.962 (0.962) |
| OD | -0.003 [0.080] | -0.17 \| 0.16 | 0.996 (0.976) | -0.013 [0.080] | -0.18 \| 0.15 | 0.890 (0.873) |
| **uncF** | | | | | | |
| Overall diffusion | 0.071 [0.060] | -0.05 \| 0.19 | 0.876 (0.249) | 0.063 [0.060] | -0.06 \| 0.18 | 0.897 (0.305) |
| AD | 0.051 [0.090] | -0.12 \| 0.22 | 0.812 (0.561) | 0.044 [0.090] | -0.13 \| 0.22 | 0.930 (0.620) |
| FA | 0.100 [0.090] | -0.07 \| 0.27 | 0.820 (0.253) | 0.093 [0.090] | -0.08 \| 0.26 | 0.882 (0.292) |
| MD | 0.031 [0.090] | -0.14 \| 0.2 | 0.815 (0.724) | 0.024 [0.090] | -0.15 \| 0.2 | 0.961 (0.789) |
| RD | 0.083 [0.090] | -0.09 \| 0.25 | 0.705 (0.343) | 0.076 [0.090] | -0.1 \| 0.25 | 0.720 (0.389) |
| ND | 0.082 [0.090] | -0.09 \| 0.25 | 0.920 (0.352) | 0.074 [0.090] | -0.1 \| 0.25 | 0.962 (0.399) |
| OD | 0.077 [0.090] | -0.1 \| 0.25 | 0.996 (0.381) | 0.069 [0.090] | -0.1 \| 0.24 | 0.890 (0.430) |
| False Discovery rate (FDR) significant differences are marked in bold. P<0.05, uncorrected significant results are underlined. *Corrected for age, sex and education in years; Abbreviations: FA = fractional anisotropy, MD = mean diffusivity, RD = radial diffusivity, AD = axial diffusivity, ND = neurite density, OD = orientation dispersion, SLF = superior longitudinal fascicle, CC genu = genu of the corpus callosum, CC body = body of the corpus callosum, CC splenium = splenium of the corpus callosum, uncF = uncinate fascicle, Sag Stratum = sagittal stratum, Posterior thal rad = posterior thalamic radiation. | | | | | | |

| **Supplementary Table 5 – Age of OCD onset mixed model analyses of white matter microstructure – random effect for site** | | | | | | | |
| --- | --- | --- | --- | --- | --- | --- | --- |
|  | **Group difference (crude model)** | | | **Group difference (adjusted model)*** | | | |
|  | B [SE] | 95% CI | P (unc) | B [SE] | 95% CI | P (unc) | |
| **CC genu** | | | | | | | |
| Overall diffusion | 0.139 [0.060] | 0.01 \| 0.27 | 0.088 (0.031) | 0.057 [0.060] | -0.06 \| 0.18 | 0.462 (0.359) | |
| AD | 0.036 [0.120] | -0.2 \| 0.27 | 0.864 (0.766) | -0.045 [0.120] | -0.28 \| 0.19 | 0.905 (0.704) | |
| FA | 0.247 [0.120] | 0.01 \| 0.48 | 0.090 (0.040) | 0.166 [0.120] | -0.07 \| 0.40 | 0.299 (0.166) | |
| MD | 0.146 [0.120] | -0.09 \| 0.38 | 0.396 (0.226) | 0.065 [0.120] | -0.17 \| 0.30 | 0.664 (0.590) | |
| RD | 0.222 [0.120] | -0.01 \| 0.46 | 0.153 (0.066) | 0.140 [0.120] | -0.09 \| 0.38 | 0.272 (0.242) | |
| ND | 0.042 [0.120] | -0.19 \| 0.28 | 0.907 (0.726) | -0.039 [0.120] | -0.27 \| 0.20 | 0.820 (0.744) | |
| OD | 0.139 [0.120] | -0.1 \| 0.38 | 0.747 (0.249) | 0.058 [0.120] | -0.17 \| 0.29 | 0.920 (0.630) | |
| **CC body** | | | | | | | |
| Overall diffusion | 0.139 [0.060] | 0.01 \| 0.27 | 0.088 (0.033) | 0.112 [0.070] | -0.02 \| 0.24 | 0.309 (0.094) | |
| AD | 0.020 [0.120] | -0.21 \| 0.25 | 0.864 (0.864) | -0.007 [0.120] | -0.24 \| 0.23 | 0.973 (0.956) | |
| FA | 0.223 [0.120] | -0.01 \| 0.46 | 0.108 (0.060) | 0.196 [0.120] | -0.04 \| 0.43 | 0.227 (0.101) | |
| MD | 0.133 [0.120] | -0.1 \| 0.37 | 0.396 (0.264) | 0.106 [0.120] | -0.13 \| 0.34 | 0.606 (0.378) | |
| RD | 0.185 [0.120] | -0.04 \| 0.42 | 0.153 (0.119) | 0.158 [0.120] | -0.07 \| 0.39 | 0.272 (0.186) | |
| ND | 0.127 [0.120] | -0.1 \| 0.36 | 0.641 (0.285) | 0.100 [0.120] | -0.13 \| 0.34 | 0.820 (0.404) | |
| OD | 0.146 [0.120] | -0.09 \| 0.38 | 0.747 (0.220) | 0.119 [0.120] | -0.11 \| 0.35 | 0.920 (0.322) | |
| **CC splenium** | | | | | | | |
| Overall diffusion | 0.137 [0.070] | 0.01 \| 0.27 | 0.088 (0.039) | 0.086 [0.070] | -0.04 \| 0.22 | 0.322 (0.200) | |
| AD | 0.208 [0.120] | -0.03 \| 0.44 | 0.576 (0.084) | 0.156 [0.120] | -0.08 \| 0.39 | 0.583 (0.195) | |
| FA | 0.185 [0.120] | -0.05 \| 0.42 | 0.188 (0.125) | 0.133 [0.120] | -0.1 \| 0.37 | 0.375 (0.269) | |
| MD | 0.040 [0.120] | -0.19 \| 0.28 | 0.736 (0.736) | -0.011 [0.120] | -0.25 \| 0.22 | 0.929 (0.929) | |
| RD | 0.195 [0.120] | -0.04 \| 0.43 | 0.153 (0.105) | 0.144 [0.120] | -0.09 \| 0.38 | 0.272 (0.234) | |
| ND | -0.005 [0.120] | -0.24 \| 0.23 | 0.966 (0.966) | -0.056 [0.120] | -0.29 \| 0.18 | 0.820 (0.640) | |
| OD | 0.202 [0.120] | -0.03 \| 0.44 | 0.747 (0.093) | 0.151 [0.120] | -0.08 \| 0.39 | 0.920 (0.211) | |
| **Dorsal cingulum** | | | | | | | |
| Overall diffusion | 0.076 [0.090] | -0.09 \| 0.25 | 0.428 (0.380) | 0.036 [0.090] | -0.13 \| 0.21 | | 0.762 (0.677) |
| AD | -0.181 [0.120] | -0.41 \| 0.05 | 0.576 (0.128) | -0.221 [0.120] | -0.45 \| 0.01 | | 0.567 (0.063) |
| FA | 0.114 [0.120] | -0.12 \| 0.35 | 0.378 (0.336) | 0.074 [0.120] | -0.16 \| 0.31 | | 0.578 (0.530) |
| MD | 0.228 [0.120] | <0.001 \| 0.46 | 0.168 (0.056) | 0.188 [0.120] | -0.04 \| 0.42 | | 0.342 (0.114) |
| RD | 0.187 [0.120] | -0.04 \| 0.42 | 0.153 (0.116) | 0.147 [0.120] | -0.08 \| 0.38 | | 0.272 (0.215) |
| ND | 0.130 [0.120] | -0.1 \| 0.36 | 0.641 (0.273) | 0.090 [0.120] | -0.14 \| 0.32 | | 0.820 (0.446) |
| OD | -0.024 [0.120] | -0.25 \| 0.21 | 0.883 (0.839) | -0.064 [0.120] | -0.29 \| 0.17 | | 0.920 (0.589) |
| **Ventral cingulum** | | | | | | | |
| Overall diffusion | -0.132 [0.070] | -0.27 \| 0.01 | 0.108 (0.060) | -0.114 [0.070] | -0.25 \| 0.03 | | 0.309 (0.103) |
| AD | -0.064 [0.120] | -0.29 \| 0.17 | 0.864 (0.588) | -0.046 [0.120] | -0.27 \| 0.18 | | 0.905 (0.695) |
| FA | -0.141 [0.120] | -0.37 \| 0.09 | 0.294 (0.229) | -0.124 [0.120] | -0.35 \| 0.11 | | 0.375 (0.292) |
| MD | -0.147 [0.120] | -0.38 \| 0.08 | 0.396 (0.209) | -0.130 [0.120] | -0.36 \| 0.10 | | 0.605 (0.269) |
| RD | -0.208 [0.120] | -0.44 \| 0.02 | 0.153 (0.076) | -0.191 [0.120] | -0.42 \| 0.04 | | 0.272 (0.104) |
| ND | -0.150 [0.120] | -0.38 \| 0.08 | 0.641 (0.201) | -0.133 [0.120] | -0.36 \| 0.10 | | 0.820 (0.259) |
| OD | -0.082 [0.120] | -0.31 \| 0.15 | 0.877 (0.487) | -0.064 [0.120] | -0.29 \| 0.17 | | 0.920 (0.585) |
| **Posterior thal rad** | | | | | | | |
| Overall diffusion | 0.215 [0.070] | 0.08 \| 0.35 | **0.018** (0.002) | 0.163 [0.070] | 0.03 \| 0.3 | | 0.198 (0.022) |
| AD | -0.075 [0.110] | -0.3 \| 0.15 | 0.864 (0.506) | -0.128 [0.110] | -0.35 \| 0.1 | | 0.583 (0.259) |
| FA | 0.360 [0.110] | 0.14 \| 0.58 | **0.009** (0.001) | 0.307 [0.110] | 0.09 \| 0.53 | | 0.063 (0.007) |
| MD | 0.318 [0.110] | 0.1 \| 0.54 | **0.045** (0.005) | 0.265 [0.110] | 0.04 \| 0.49 | | 0.180 (0.020) |
| RD | 0.380 [0.110] | 0.16 \| 0.60 | **0.009** (0.001) | 0.327 [0.110] | 0.11 \| 0.55 | | **0.036** (0.004) |
| ND | 0.269 [0.110] | 0.05 \| 0.49 | 0.153 (0.017) | 0.217 [0.110] | <0.001 \| 0.44 | | 0.513 (0.057) |
| OD | 0.041 [0.110] | -0.18 \| 0.26 | 0.883 (0.714) | -0.011 [0.110] | -0.23 \| 0.21 | | 0.920 (0.920) |
| **Sag Stratum** | | | | | | | |
| Overall diffusion | 0.120 [0.080] | -0.03 \| 0.27 | 0.157 (0.122) | 0.101 [0.080] | -0.05 \| 0.26 | 0.322 (0.208) | |
| AD | 0.023 [0.120] | -0.2 \| 0.26 | 0.864 (0.843) | 0.004 [0.120] | -0.23 \| 0.24 | 0.973 (0.973) | |
| FA | 0.248 [0.120] | 0.02 \| 0.48 | 0.090 (0.035) | 0.229 [0.120] | <0.001 \| 0.46 | 0.165 (0.055) | |
| MD | 0.089 [0.120] | -0.14 \| 0.32 | 0.503 (0.447) | 0.070 [0.120] | -0.16 \| 0.30 | 0.664 (0.556) | |
| RD | 0.169 [0.120] | -0.06 \| 0.4 | 0.169 (0.150) | 0.150 [0.120] | -0.08 \| 0.38 | 0.272 (0.208) | |
| ND | 0.099 [0.120] | -0.13 \| 0.33 | 0.718 (0.399) | 0.080 [0.120] | -0.15 \| 0.32 | 0.820 (0.503) | |
| OD | 0.091 [0.120] | -0.14 \| 0.32 | 0.877 (0.441) | 0.071 [0.120] | -0.16 \| 0.31 | 0.920 (0.549) | |
| **SLF** | | | | | | | |
| Overall diffusion | 0.130 [0.070] | -0.01 \| 0.28 | 0.122 (0.081) | 0.095 [0.080] | -0.05 \| 0.25 | 0.322 (0.215) | |
| AD | -0.122 [0.120] | -0.35 \| 0.11 | 0.864 (0.296) | -0.158 [0.120] | -0.39 \| 0.07 | 0.583 (0.180) | |
| FA | 0.283 [0.120] | 0.06 \| 0.51 | 0.072 (0.016) | 0.247 [0.120] | 0.02 \| 0.48 | 0.165 (0.037) | |
| MD | 0.242 [0.120] | 0.01 \| 0.47 | 0.168 (0.039) | 0.206 [0.120] | -0.02 \| 0.44 | 0.342 (0.081) | |
| RD | 0.295 [0.120] | 0.07 \| 0.53 | 0.054 (0.012) | 0.259 [0.120] | 0.03 \| 0.49 | 0.130 (0.029) | |
| ND | 0.069 [0.120] | -0.16 \| 0.30 | 0.837 (0.558) | 0.033 [0.120] | -0.2 \| 0.27 | 0.820 (0.782) | |
| OD | 0.017 [0.120] | -0.21 \| 0.25 | 0.883 (0.883) | -0.019 [0.120] | -0.25 \| 0.21 | 0.920 (0.874) | |
| **uncF** | | | | | | | |
| Overall diffusion | -0.017 [0.080] | -0.18 \| 0.15 | 0.843 (0.843) | -0.015 [0.090] | -0.18 \| 0.16 | | 0.864 (0.864) |
| AD | -0.060 [0.120] | -0.29 \| 0.18 | 0.864 (0.620) | -0.058 [0.120] | -0.29 \| 0.19 | | 0.905 (0.635) |
| FA | -0.070 [0.120] | -0.3 \| 0.17 | 0.563 (0.563) | -0.068 [0.120] | -0.3 \| 0.18 | | 0.578 (0.578) |
| MD | 0.100 [0.120] | -0.13 \| 0.34 | 0.503 (0.406) | 0.102 [0.120] | -0.13 \| 0.35 | | 0.606 (0.404) |
| RD | -0.012 [0.120] | -0.24 \| 0.23 | 0.920 (0.920) | -0.010 [0.120] | -0.24 \| 0.23 | | 0.933 (0.933) |
| ND | -0.030 [0.120] | -0.26 \| 0.21 | 0.907 (0.806) | -0.028 [0.120] | -0.26 \| 0.22 | | 0.820 (0.820) |
| OD | -0.028 [0.120] | -0.26 \| 0.21 | 0.883 (0.814) | -0.027 [0.120] | -0.26 \| 0.22 | | 0.920 (0.828) |
| False Discovery rate (FDR) significant differences are marked in bold. P<0.05, uncorrected significant results are underlined. *Corrected for age, sex and education in years; Abbreviations: FA = fractional anisotropy, MD = mean diffusivity, RD = radial diffusivity, AD = axial diffusivity, ND = neurite density, OD = orientation dispersion, SLF = superior longitudinal fascicle, CC genu = genu of the corpus callosum, CC body = body of the corpus callosum, CC splenium = splenium of the corpus callosum. | | | | | | | |

| **Supplementary Table 6 – SSRI/SNRI history mixed model analyses of white matter microstructure – ComBat corrected** | | | | | | | | |
| --- | --- | --- | --- | --- | --- | --- | --- | --- |
|  | **Naive** | **Not naive** | **Group difference (crude model)** | | | **Group difference (adjusted model)*** | | |
|  | M ± SD | M ± SD | B [SE] | 95% CI | P (unc) | B [SE] | 95% CI | P (unc) |
| **CC genu** | | | | | | | | |
| Overall diffusion |  |  | 0.050 [0.070] | -0.1 \| 0.2 | 0.870 (0.504) | 0.089 [0.070] | -0.05 \| 0.22 | 0.527 (0.209) |
| AD | 1.524 ± 0.05 | 1.521 ± 0.05 | -0.054 [0.120] | -0.3 \| 0.19 | 0.940 (0.663) | -0.015 [0.120] | -0.25 \| 0.22 | 0.900 (0.900) |
| FA | 0.751 ± 0.034 | 0.755 ± 0.034 | 0.129 [0.120] | -0.11 \| 0.37 | 0.689 (0.299) | 0.167 [0.120] | -0.07 \| 0.4 | 0.540 (0.168) |
| MD | 0.732 ± 0.036 | 0.728 ± 0.035 | 0.100 [0.120] | -0.14 \| 0.34 | 0.982 (0.420) | 0.139 [0.120] | -0.1 \| 0.38 | 0.928 (0.254) |
| RD | 0.339 ± 0.044 | 0.333 ± 0.043 | 0.134 [0.120] | -0.11 \| 0.38 | 0.826 (0.280) | 0.172 [0.120] | -0.06 \| 0.41 | 0.673 (0.156) |
| ND | 0.65 ± 0.038 | 0.648 ± 0.038 | -0.050 [0.120] | -0.29 \| 0.19 | 0.908 (0.685) | -0.012 [0.120] | -0.25 \| 0.22 | 0.949 (0.924) |
| OD | 0.087 ± 0.01 | 0.086 ± 0.01 | 0.041 [0.120] | -0.2 \| 0.28 | 0.970 (0.740) | 0.080 [0.120] | -0.16 \| 0.32 | 0.958 (0.511) |
| **CC body** | | | | | | | | |
| Overall diffusion |  |  | 0.071 [0.070] | -0.07 \| 0.22 | 0.740 (0.329) | 0.087 [0.070] | -0.06 \| 0.23 | 0.527 (0.234) |
| AD | 1.507 ± 0.043 | 1.518 ± 0.05 | 0.231 [0.120] | -0.01 \| 0.47 | 0.513 (0.057) | 0.247 [0.120] | 0.01 \| 0.48 | 0.378 (0.042) |
| FA | 0.74 ± 0.039 | 0.744 ± 0.036 | 0.093 [0.120] | -0.14 \| 0.33 | 0.689 (0.444) | 0.109 [0.120] | -0.13 \| 0.35 | 0.690 (0.371) |
| MD | 0.725 ± 0.033 | 0.725 ± 0.034 | -0.011 [0.120] | -0.25 \| 0.23 | 0.982 (0.929) | 0.005 [0.120] | -0.23 \| 0.24 | 0.969 (0.969) |
| RD | 0.348 ± 0.048 | 0.345 ± 0.046 | 0.049 [0.120] | -0.19 \| 0.29 | 0.884 (0.690) | 0.064 [0.120] | -0.17 \| 0.30 | 0.673 (0.598) |
| ND | 0.658 ± 0.033 | 0.655 ± 0.033 | -0.092 [0.120] | -0.33 \| 0.14 | 0.908 (0.449) | -0.077 [0.120] | -0.31 \| 0.16 | 0.949 (0.529) |
| OD | 0.083 ± 0.008 | 0.082 ± 0.009 | 0.159 [0.120] | -0.08 \| 0.4 | 0.864 (0.192) | 0.174 [0.120] | -0.06 \| 0.41 | 0.684 (0.152) |
| **CC splenium** | | | | | | | | |
| Overall diffusion |  |  | -0.036 [0.080] | -0.18 \| 0.11 | 0.870 (0.630) | -0.017 [0.070] | -0.16 \| 0.13 | 0.920 (0.818) |
| AD | 1.591 ± 0.05 | 1.591 ± 0.052 | 0.010 [0.120] | -0.23 \| 0.25 | 0.940 (0.939) | 0.029 [0.120] | -0.21 \| 0.27 | 0.900 (0.816) |
| FA | 0.836 ± 0.021 | 0.834 ± 0.021 | -0.096 [0.120] | -0.34 \| 0.15 | 0.689 (0.442) | -0.077 [0.120] | -0.32 \| 0.16 | 0.690 (0.537) |
| MD | 0.699 ± 0.025 | 0.699 ± 0.027 | -0.003 [0.120] | -0.25 \| 0.24 | 0.982 (0.982) | 0.017 [0.120] | -0.22 \| 0.26 | 0.969 (0.894) |
| RD | 0.24 ± 0.027 | 0.243 ± 0.027 | -0.092 [0.120] | -0.34 \| 0.15 | 0.826 (0.459) | -0.073 [0.120] | -0.32 \| 0.17 | 0.673 (0.556) |
| ND | 0.724 ± 0.029 | 0.723 ± 0.032 | -0.011 [0.120] | -0.26 \| 0.23 | 1.000 (0.927) | 0.008 [0.120] | -0.23 \| 0.25 | 0.949 (0.949) |
| OD | 0.07 ± 0.01 | 0.07 ± 0.01 | -0.026 [0.120] | -0.27 \| 0.22 | 0.970 (0.836) | -0.006 [0.120] | -0.25 \| 0.23 | 0.958 (0.958) |
| **Dorsal cingulum** | | | | | | | | |
| Overall diffusion |  |  | 0.015 [0.090] | -0.17 \| 0.20 | 0.870 (0.870) | 0.024 [0.090] | -0.15 \| 0.20 | 0.920 (0.794) |
| AD | 1.274 ± 0.054 | 1.275 ± 0.056 | 0.009 [0.120] | -0.23 \| 0.25 | 0.940 (0.940) | 0.018 [0.120] | -0.22 \| 0.26 | 0.900 (0.885) |
| FA | 0.591 ± 0.044 | 0.592 ± 0.044 | 0.023 [0.120] | -0.22 \| 0.27 | 0.851 (0.851) | 0.032 [0.120] | -0.21 \| 0.27 | 0.894 (0.795) |
| MD | 0.722 ± 0.025 | 0.721 ± 0.026 | 0.031 [0.120] | -0.21 \| 0.28 | 0.982 (0.801) | 0.040 [0.120] | -0.2 \| 0.28 | 0.959 (0.746) |
| RD | 0.446 ± 0.037 | 0.444 ± 0.038 | 0.058 [0.120] | -0.18 \| 0.30 | 0.884 (0.639) | 0.067 [0.120] | -0.17 \| 0.30 | 0.673 (0.585) |
| ND | 0.599 ± 0.033 | 0.597 ± 0.032 | -0.047 [0.120] | -0.29 \| 0.20 | 0.908 (0.706) | -0.039 [0.120] | -0.28 \| 0.20 | 0.949 (0.752) |
| OD | 0.138 ± 0.017 | 0.137 ± 0.017 | 0.017 [0.120] | -0.23 \| 0.26 | 0.970 (0.891) | 0.025 [0.120] | -0.21 \| 0.26 | 0.958 (0.835) |
| **Cing2** | | | | | | | | |
| Overall diffusion |  |  | 0.122 [0.080] | -0.03 \| 0.27 | 0.740 (0.110) | 0.099 [0.070] | -0.05 \| 0.24 | 0.527 (0.184) |
| AD | 1.25 ± 0.071 | 1.262 ± 0.089 | 0.150 [0.120] | -0.09 \| 0.38 | 0.670 (0.211) | 0.127 [0.120] | -0.1 \| 0.36 | 0.824 (0.285) |
| FA | 0.487 ± 0.042 | 0.495 ± 0.047 | 0.183 [0.120] | -0.05 \| 0.42 | 0.689 (0.129) | 0.160 [0.120] | -0.07 \| 0.39 | 0.540 (0.180) |
| MD | 0.748 ± 0.038 | 0.75 ± 0.051 | -0.036 [0.120] | -0.27 \| 0.20 | 0.982 (0.764) | -0.059 [0.120] | -0.29 \| 0.17 | 0.928 (0.619) |
| RD | 0.495 ± 0.044 | 0.49 ± 0.050 | 0.095 [0.120] | -0.14 \| 0.33 | 0.826 (0.429) | 0.072 [0.120] | -0.16 \| 0.30 | 0.673 (0.545) |
| ND | 0.505 ± 0.034 | 0.508 ± 0.037 | 0.097 [0.120] | -0.14 \| 0.33 | 0.908 (0.419) | 0.074 [0.120] | -0.16 \| 0.31 | 0.949 (0.533) |
| OD | 0.166 ± 0.022 | 0.161 ± 0.022 | 0.241 [0.120] | 0.01 \| 0.48 | 0.405 (0.045) | 0.218 [0.120] | -0.01 \| 0.45 | 0.603 (0.067) |
| **Posterior thal rad** | | | | | | | | |
| Overall diffusion |  |  | 0.022 [0.080] | -0.14 \| 0.18 | 0.870 (0.790) | 0.064 [0.080] | -0.09 \| 0.22 | 0.625 (0.417) |
| AD | 1.375 ± 0.045 | 1.374 ± 0.045 | -0.019 [0.120] | -0.26 \| 0.22 | 0.940 (0.876) | 0.023 [0.120] | -0.22 \| 0.26 | 0.900 (0.855) |
| FA | 0.604 ± 0.032 | 0.606 ± 0.031 | 0.050 [0.120] | -0.19 \| 0.29 | 0.851 (0.687) | 0.092 [0.120] | -0.15 \| 0.33 | 0.690 (0.454) |
| MD | 0.776 ± 0.029 | 0.774 ± 0.030 | 0.045 [0.120] | -0.2 \| 0.29 | 0.982 (0.716) | 0.087 [0.120] | -0.15 \| 0.33 | 0.928 (0.478) |
| RD | 0.469 ± 0.036 | 0.468 ± 0.035 | 0.034 [0.120] | -0.21 \| 0.28 | 0.884 (0.786) | 0.076 [0.120] | -0.16 \| 0.32 | 0.673 (0.538) |
| ND | 0.567 ± 0.035 | 0.567 ± 0.037 | <0.001 [0.120] | -0.24 \| 0.24 | 1.000 (1.000) | 0.042 [0.120] | -0.2 \| 0.28 | 0.949 (0.733) |
| OD | 0.119 ± 0.012 | 0.119 ± 0.011 | 0.019 [0.120] | -0.22 \| 0.26 | 0.970 (0.879) | 0.061 [0.120] | -0.18 \| 0.30 | 0.958 (0.620) |
| **Sag Stratum** | | | | | | | | |
| Overall diffusion |  |  | -0.019 [0.090] | -0.18 \| 0.15 | 0.870 (0.827) | 0.002 [0.090] | -0.16 \| 0.17 | 0.984 (0.984) |
| AD | 1.331 ± 0.044 | 1.325 ± 0.043 | -0.134 [0.120] | -0.38 \| 0.11 | 0.670 (0.286) | -0.113 [0.120] | -0.36 \| 0.13 | 0.824 (0.366) |
| FA | 0.571 ± 0.031 | 0.57 ± 0.032 | -0.034 [0.120] | -0.28 \| 0.21 | 0.851 (0.785) | -0.014 [0.120] | -0.26 \| 0.23 | 0.913 (0.913) |
| MD | 0.782 ± 0.026 | 0.78 ± 0.029 | 0.046 [0.120] | -0.2 \| 0.29 | 0.982 (0.716) | 0.066 [0.120] | -0.18 \| 0.31 | 0.928 (0.599) |
| RD | 0.513 ± 0.033 | 0.512 ± 0.037 | 0.011 [0.120] | -0.23 \| 0.26 | 0.929 (0.929) | 0.031 [0.120] | -0.21 \| 0.28 | 0.802 (0.802) |
| ND | 0.542 ± 0.035 | 0.543 ± 0.036 | 0.048 [0.120] | -0.2 \| 0.29 | 0.908 (0.701) | 0.069 [0.120] | -0.18 \| 0.31 | 0.949 (0.585) |
| OD | 0.141 ± 0.012 | 0.142 ± 0.012 | -0.049 [0.120] | -0.29 \| 0.20 | 0.970 (0.698) | -0.028 [0.120] | -0.27 \| 0.22 | 0.958 (0.822) |
| **SLF** | | | | | | | | |
| Overall diffusion |  |  | 0.109 [0.080] | -0.06 \| 0.27 | 0.740 (0.194) | 0.124 [0.080] | -0.04 \| 0.29 | 0.527 (0.134) |
| AD | 1.119 ± 0.031 | 1.12 ± 0.038 | 0.047 [0.120] | -0.2 \| 0.29 | 0.940 (0.707) | 0.062 [0.120] | -0.18 \| 0.3 | 0.900 (0.615) |
| FA | 0.509 ± 0.025 | 0.514 ± 0.027 | 0.175 [0.120] | -0.07 \| 0.42 | 0.689 (0.162) | 0.190 [0.120] | -0.05 \| 0.43 | 0.540 (0.126) |
| MD | 0.695 ± 0.020 | 0.693 ± 0.024 | 0.086 [0.120] | -0.16 \| 0.33 | 0.982 (0.491) | 0.102 [0.120] | -0.14 \| 0.34 | 0.928 (0.414) |
| RD | 0.483 ± 0.024 | 0.48 ± 0.0280 | 0.139 [0.120] | -0.1 \| 0.38 | 0.826 (0.265) | 0.155 [0.120] | -0.09 \| 0.40 | 0.673 (0.213) |
| ND | 0.671 ± 0.031 | 0.673 ± 0.031 | 0.084 [0.120] | -0.16 \| 0.33 | 0.908 (0.500) | 0.100 [0.120] | -0.14 \| 0.34 | 0.949 (0.422) |
| OD | 0.196 ± 0.012 | 0.194 ± 0.014 | 0.122 [0.120] | -0.12 \| 0.37 | 0.970 (0.328) | 0.138 [0.120] | -0.1 \| 0.38 | 0.804 (0.268) |
| **uncF** | | | | | | | | |
| Overall diffusion |  |  | 0.092 [0.090] | -0.08 \| 0.26 | 0.740 (0.300) | 0.085 [0.090] | -0.09 \| 0.26 | 0.605 (0.336) |
| AD | 1.251 ± 0.052 | 1.244 ± 0.052 | -0.126 [0.120] | -0.36 \| 0.11 | 0.670 (0.298) | -0.132 [0.120] | -0.37 \| 0.1 | 0.824 (0.275) |
| FA | 0.506 ± 0.045 | 0.51 ± 0.047 | 0.089 [0.120] | -0.15 \| 0.32 | 0.689 (0.459) | 0.083 [0.120] | -0.15 \| 0.32 | 0.690 (0.492) |
| MD | 0.768 ± 0.031 | 0.76 ± 0.032 | 0.242 [0.120] | 0.01 \| 0.48 | 0.414 (0.046) | 0.235 [0.120] | <0.001 \| 0.47 | 0.468 (0.052) |
| RD | 0.53 ± 0.0430 | 0.523 ± 0.045 | 0.149 [0.120] | -0.09 \| 0.38 | 0.826 (0.219) | 0.142 [0.120] | -0.09 \| 0.38 | 0.673 (0.240) |
| ND | 0.503 ± 0.031 | 0.509 ± 0.033 | 0.200 [0.120] | -0.04 \| 0.44 | 0.891 (0.099) | 0.193 [0.120] | -0.04 \| 0.43 | 0.949 (0.110) |
| OD | 0.152 ± 0.017 | 0.153 ± 0.016 | -0.004 [0.120] | -0.24 \| 0.23 | 0.970 (0.970) | -0.011 [0.120] | -0.25 \| 0.22 | 0.958 (0.929) |
| False Discovery rate (FDR) significant differences are marked in bold. P<0.05, uncorrected significant results are underlined. *Corrected for age, sex and education in years; Abbreviations: FA = fractional anisotropy, MD = mean diffusivity, RD = radial diffusivity, AD = axial diffusivity, ND = neurite density, OD = orientation dispersion SLF = superior longitudinal fascicle, CC genu = genu of the corpus callosum, CC body = body of the corpus callosum, CC splenium = splenium of the corpus callosum, uncF = uncinate fascicle, Sag Stratum = sagittal stratum, Posterior thal rad = posterior thalamic radiation. | | | | | | | | |

| **Supplementary Table 7 – SSRI/SNRI history mixed model analyses of white matter microstructure – random effects for site** | | | | | | |
| --- | --- | --- | --- | --- | --- | --- |
|  | **Group difference (crude model)** | | | **Group difference (adjusted model)*** | | |
|  | B [SE] | 95% CI | P (unc) | B [SE] | 95% CI | P (unc) |
| **CC genu** | | | | | | |
| Overall diffusion | 0.062 [0.060] | -0.06 \| 0.19 | 0.671 (0.337) | 0.096 [0.060] | -0.02 \| 0.21 | 0.417 (0.111) |
| AD | -0.098 [0.120] | -0.33 \| 0.14 | 0.879 (0.418) | -0.063 [0.120] | -0.3 \| 0.17 | 0.974 (0.597) |
| FA | 0.089 [0.120] | -0.15 \| 0.32 | 0.736 (0.462) | 0.123 [0.120] | -0.11 \| 0.35 | 0.774 (0.298) |
| MD | 0.141 [0.120] | -0.10 \| 0.38 | 0.657 (0.241) | 0.176 [0.120] | -0.06 \| 0.41 | 0.481 (0.137) |
| RD | 0.103 [0.120] | -0.13 \| 0.34 | 0.704 (0.391) | 0.138 [0.120] | -0.09 \| 0.37 | 0.713 (0.244) |
| ND | 0.280 [0.120] | 0.04 \| 0.52 | 0.060 (0.020) | 0.315 [0.120] | 0.08 \| 0.55 | **0.024** (0.008) |
| OD | -0.146 [0.120] | -0.38 \| 0.09 | 0.678 (0.226) | -0.111 [0.120] | -0.34 \| 0.12 | 0.904 (0.349) |
| **CC body** | | | | | | |
| Overall diffusion | 0.083 [0.060] | -0.04 \| 0.21 | 0.609 (0.203) | 0.097 [0.060] | -0.03 \| 0.23 | 0.417 (0.139) |
| AD | -0.033 [0.120] | -0.26 \| 0.2 | 0.879 (0.781) | -0.020 [0.120] | -0.25 \| 0.22 | 0.974 (0.870) |
| FA | 0.151 [0.120] | -0.08 \| 0.39 | 0.736 (0.203) | 0.165 [0.120] | -0.06 \| 0.40 | 0.774 (0.166) |
| MD | 0.121 [0.120] | -0.11 \| 0.36 | 0.657 (0.310) | 0.134 [0.120] | -0.10 \| 0.37 | 0.481 (0.258) |
| RD | 0.133 [0.120] | -0.10 \| 0.37 | 0.704 (0.265) | 0.146 [0.120] | -0.08 \| 0.38 | 0.713 (0.219) |
| ND | 0.167 [0.120] | -0.06 \| 0.40 | 0.182 (0.162) | 0.180 [0.120] | -0.05 \| 0.42 | 0.130 (0.130) |
| OD | -0.041 [0.120] | -0.27 \| 0.20 | 0.899 (0.733) | -0.027 [0.120] | -0.26 \| 0.21 | 0.904 (0.820) |
| **CC splenium** | | | | | | |
| Overall diffusion | -0.028 [0.070] | -0.16 \| 0.1 | 0.764 (0.679) | -0.007 [0.060] | -0.14 \| 0.12 | 0.917 (0.917) |
| AD | -0.055 [0.120] | -0.29 \| 0.18 | 0.879 (0.646) | -0.034 [0.120] | -0.27 \| 0.2 | 0.974 (0.774) |
| FA | -0.138 [0.120] | -0.37 \| 0.1 | 0.736 (0.251) | -0.117 [0.120] | -0.35 \| 0.12 | 0.774 (0.327) |
| MD | 0.011 [0.120] | -0.22 \| 0.25 | 0.924 (0.924) | 0.032 [0.120] | -0.2 \| 0.26 | 0.887 (0.788) |
| RD | -0.137 [0.120] | -0.37 \| 0.1 | 0.704 (0.255) | -0.116 [0.120] | -0.35 \| 0.12 | 0.713 (0.332) |
| ND | 0.312 [0.120] | 0.08 \| 0.55 | **0.045** (0.010) | 0.332 [0.120] | 0.1 \| 0.56 | **0.024** (0.005) |
| OD | -0.159 [0.120] | -0.39 \| 0.07 | 0.678 (0.186) | -0.138 [0.120] | -0.37 \| 0.09 | 0.904 (0.248) |
| **Dorsal cingulum** | | | | | | |
| Overall diffusion | 0.037 [0.090] | -0.13 \| 0.2 | 0.764 (0.664) | 0.048 [0.080] | -0.12 \| 0.21 | 0.725 (0.564) |
| AD | -0.045 [0.120] | -0.28 \| 0.19 | 0.879 (0.706) | -0.034 [0.120] | -0.26 \| 0.19 | 0.974 (0.770) |
| FA | -0.004 [0.120] | -0.24 \| 0.23 | 0.975 (0.975) | 0.007 [0.120] | -0.22 \| 0.23 | 0.952 (0.952) |
| MD | 0.092 [0.120] | -0.14 \| 0.32 | 0.657 (0.438) | 0.103 [0.120] | -0.13 \| 0.33 | 0.569 (0.379) |
| RD | 0.050 [0.120] | -0.18 \| 0.28 | 0.792 (0.672) | 0.061 [0.120] | -0.17 \| 0.29 | 0.713 (0.602) |
| ND | 0.209 [0.120] | -0.02 \| 0.44 | 0.102 (0.079) | 0.219 [0.120] | -0.01 \| 0.45 | 0.078 (0.061) |
| OD | -0.079 [0.120] | -0.31 \| 0.15 | 0.899 (0.506) | -0.068 [0.120] | -0.3 \| 0.16 | 0.904 (0.559) |
| **Ventral cingulum** | | | | | | |
| Overall diffusion | 0.102 [0.070] | -0.04 \| 0.24 | 0.609 (0.145) | 0.087 [0.070] | -0.05 \| 0.22 | 0.459 (0.204) |
| AD | 0.096 [0.120] | -0.13 \| 0.32 | 0.879 (0.414) | 0.080 [0.120] | -0.15 \| 0.31 | 0.974 (0.490) |
| FA | 0.089 [0.120] | -0.14 \| 0.32 | 0.736 (0.447) | 0.074 [0.120] | -0.15 \| 0.30 | 0.789 (0.526) |
| MD | 0.015 [0.120] | -0.22 \| 0.24 | 0.924 (0.900) | -0.001 [0.120] | -0.23 \| 0.22 | 0.995 (0.995) |
| RD | 0.107 [0.120] | -0.12 \| 0.34 | 0.704 (0.361) | 0.092 [0.120] | -0.14 \| 0.32 | 0.713 (0.430) |
| ND | 0.255 [0.120] | 0.03 \| 0.48 | 0.068 (0.030) | 0.239 [0.120] | 0.01 \| 0.47 | 0.072 (0.040) |
| OD | 0.050 [0.120] | -0.18 \| 0.28 | 0.899 (0.668) | 0.035 [0.120] | -0.19 \| 0.26 | 0.904 (0.764) |
| **Posterior thal rad** | | | | | | |
| Overall diffusion | 0.031 [0.070] | -0.11 \| 0.17 | 0.764 (0.665) | 0.059 [0.070] | -0.08 \| 0.19 | 0.612 (0.393) |
| AD | -0.075 [0.110] | -0.3 \| 0.15 | 0.879 (0.511) | -0.047 [0.110] | -0.27 \| 0.17 | 0.974 (0.681) |
| FA | 0.024 [0.110] | -0.2 \| 0.25 | 0.937 (0.833) | 0.052 [0.110] | -0.17 \| 0.27 | 0.827 (0.643) |
| MD | 0.097 [0.110] | -0.13 \| 0.32 | 0.657 (0.394) | 0.126 [0.110] | -0.10 \| 0.35 | 0.481 (0.267) |
| RD | 0.037 [0.110] | -0.19 \| 0.26 | 0.792 (0.744) | 0.066 [0.110] | -0.16 \| 0.29 | 0.713 (0.561) |
| ND | 0.143 [0.110] | -0.08 \| 0.37 | 0.209 (0.209) | 0.172 [0.110] | -0.05 \| 0.39 | 0.130 (0.129) |
| OD | -0.043 [0.110] | -0.27 \| 0.18 | 0.899 (0.706) | -0.015 [0.110] | -0.23 \| 0.21 | 0.904 (0.896) |
| **Sag Stratum** | | | | | | |
| Overall diffusion | -0.003 [0.080] | -0.16 \| 0.15 | 0.968 (0.968) | 0.009 [0.080] | -0.14 \| 0.16 | 0.917 (0.906) |
| AD | -0.218 [0.120] | -0.45 \| 0.01 | 0.567 (0.063) | -0.206 [0.120] | -0.44 \| 0.02 | 0.720 (0.080) |
| FA | -0.039 [0.120] | -0.27 \| 0.19 | 0.937 (0.736) | -0.027 [0.120] | -0.26 \| 0.20 | 0.919 (0.817) |
| MD | 0.136 [0.120] | -0.09 \| 0.36 | 0.657 (0.247) | 0.148 [0.120] | -0.08 \| 0.38 | 0.481 (0.208) |
| RD | 0.031 [0.120] | -0.20 \| 0.26 | 0.792 (0.792) | 0.043 [0.120] | -0.19 \| 0.27 | 0.713 (0.713) |
| ND | 0.232 [0.120] | <0.001 \| 0.46 | 0.081 (0.048) | 0.244 [0.120] | 0.01 \| 0.47 | 0.072 (0.038) |
| OD | -0.159 [0.120] | -0.39 \| 0.07 | 0.678 (0.174) | -0.147 [0.120] | -0.38 \| 0.08 | 0.904 (0.211) |
| **SLF** | | | | | | |
| Overall diffusion | 0.096 [0.070] | -0.05 \| 0.24 | 0.609 (0.196) | 0.111 [0.070] | -0.03 \| 0.25 | 0.417 (0.134) |
| AD | -0.121 [0.120] | -0.35 \| 0.11 | 0.879 (0.301) | -0.107 [0.120] | -0.33 \| 0.12 | 0.974 (0.361) |
| FA | 0.096 [0.120] | -0.13 \| 0.32 | 0.736 (0.413) | 0.111 [0.120] | -0.12 \| 0.34 | 0.774 (0.344) |
| MD | 0.182 [0.120] | -0.05 \| 0.41 | 0.657 (0.120) | 0.197 [0.120] | -0.03 \| 0.42 | 0.481 (0.092) |
| RD | 0.146 [0.120] | -0.08 \| 0.38 | 0.704 (0.213) | 0.160 [0.120] | -0.07 \| 0.39 | 0.713 (0.170) |
| ND | 0.305 [0.120] | 0.08 \| 0.53 | **0.045** (0.009) | 0.319 [0.120] | 0.09 \| 0.55 | **0.024** (0.006) |
| OD | -0.030 [0.120] | -0.26 \| 0.2 | 0.899 (0.799) | -0.015 [0.120] | -0.24 \| 0.21 | 0.904 (0.896) |
| **uncF** | | | | | | |
| Overall diffusion | 0.075 [0.080] | -0.09 \| 0.24 | 0.671 (0.373) | 0.070 [0.080] | -0.10 \| 0.23 | 0.612 (0.408) |
| AD | 0.009 [0.120] | -0.23 \| 0.25 | 0.939 (0.939) | 0.004 [0.120] | -0.23 \| 0.24 | 0.974 (0.974) |
| FA | 0.083 [0.120] | -0.15 \| 0.32 | 0.736 (0.491) | 0.078 [0.120] | -0.16 \| 0.31 | 0.789 (0.519) |
| MD | 0.072 [0.120] | -0.16 \| 0.31 | 0.708 (0.551) | 0.067 [0.120] | -0.17 \| 0.30 | 0.747 (0.581) |
| RD | 0.062 [0.120] | -0.17 \| 0.30 | 0.792 (0.610) | 0.057 [0.120] | -0.18 \| 0.29 | 0.713 (0.641) |
| ND | 0.233 [0.120] | <0.001 \| 0.47 | 0.081 (0.054) | 0.228 [0.120] | -0.01 \| 0.46 | 0.078 (0.060) |
| OD | -0.009 [0.120] | -0.25 \| 0.23 | 0.938 (0.938) | -0.015 [0.120] | -0.25 \| 0.22 | 0.904 (0.904) |
| False Discovery rate (FDR) significant differences are marked in bold. P<0.05, uncorrected significant results are underlined. *Corrected for age, sex and education in years; Abbreviations: FA = fractional anisotropy, MD = mean diffusivity, RD = radial diffusivity, AD = axial diffusivity, ND = neurite density, OD = orientation dispersion, SLF = superior longitudinal fascicle, CC genu = genu of the corpus callosum, CC body = body of the corpus callosum, CC splenium = splenium of the corpus callosum. | | | | | | |

| **Supplementary Table 8 – mixed model analyses of white matter microstructure with YBOCS – ComBat corrected** | | | | | | |
| --- | --- | --- | --- | --- | --- | --- |
|  | **Group difference (crude model)** | | | **Group difference (adjusted model)*** | | |
|  | B [SE] | 95% CI | P (unc) | B [SE] | 95% CI | P (unc) |
| **CC genu** | | | | | | |
| Overall diffusion | <0.001 [0.010] | -0.01 \| 0.01 | 0.955 (0.955) | 0.002 [0.010] | -0.01 \| 0.02 | 0.863 (0.767) |
| AD | 0.004 [0.010] | -0.02 \| 0.03 | 0.982 (0.764) | 0.005 [0.010] | -0.02 \| 0.03 | 0.774 (0.657) |
| FA | 0.004 [0.010] | -0.02 \| 0.03 | 0.892 (0.763) | 0.005 [0.010] | -0.02 \| 0.03 | 0.948 (0.656) |
| MD | <0.001 [0.010] | -0.03 \| 0.02 | 0.989 (0.989) | 0.002 [0.010] | -0.02 \| 0.03 | 0.942 (0.901) |
| RD | 0.002 [0.010] | -0.02 \| 0.03 | 0.991 (0.845) | 0.004 [0.010] | -0.02 \| 0.03 | 0.963 (0.736) |
| ND | -0.005 [0.010] | -0.03 \| 0.02 | 0.872 (0.678) | -0.003 [0.010] | -0.03 \| 0.02 | 0.777 (0.777) |
| OD | -0.002 [0.010] | -0.03 \| 0.02 | 0.973 (0.869) | <0.001 [0.010] | -0.02 \| 0.02 | 0.977 (0.977) |
| **CC body** | | | | | | |
| Overall diffusion | -0.004 [0.010] | -0.02 \| 0.01 | 0.955 (0.587) | -0.003 [0.010] | -0.02 \| 0.01 | 0.863 (0.700) |
| AD | 0.017 [0.010] | -0.01 \| 0.04 | 0.869 (0.164) | 0.018 [0.010] | -0.01 \| 0.04 | 0.626 (0.139) |
| FA | -0.008 [0.010] | -0.03 \| 0.02 | 0.892 (0.537) | -0.006 [0.010] | -0.03 \| 0.02 | 0.948 (0.602) |
| MD | -0.015 [0.010] | -0.04 \| 0.01 | 0.643 (0.220) | -0.014 [0.010] | -0.04 \| 0.01 | 0.777 (0.259) |
| RD | -0.013 [0.010] | -0.04 \| 0.01 | 0.873 (0.291) | -0.012 [0.010] | -0.04 \| 0.01 | 0.758 (0.337) |
| ND | -0.020 [0.010] | -0.04 \| <0.001 | 0.472 (0.105) | -0.019 [0.010] | -0.04 \| <0.001 | 0.743 (0.128) |
| OD | 0.014 [0.010] | -0.01 \| 0.04 | 0.934 (0.245) | 0.015 [0.010] | -0.01 \| 0.04 | 0.837 (0.212) |
| **CC splenium** | | | | | | |
| Overall diffusion | -0.001 [0.010] | -0.02 \| 0.01 | 0.955 (0.922) | 0.002 [0.010] | -0.01 \| 0.02 | 0.863 (0.767) |
| AD | 0.016 [0.010] | -0.01 \| 0.04 | 0.869 (0.193) | 0.019 [0.010] | <0.001 \| 0.04 | 0.626 (0.123) |
| FA | -0.002 [0.010] | -0.03 \| 0.02 | 0.892 (0.892) | 0.001 [0.010] | -0.02 \| 0.03 | 0.964 (0.918) |
| MD | -0.013 [0.010] | -0.04 \| 0.01 | 0.643 (0.286) | -0.010 [0.010] | -0.04 \| 0.01 | 0.914 (0.406) |
| RD | -0.007 [0.010] | -0.03 \| 0.02 | 0.991 (0.578) | -0.004 [0.010] | -0.03 \| 0.02 | 0.963 (0.749) |
| ND | -0.009 [0.010] | -0.03 \| 0.02 | 0.825 (0.475) | -0.006 [0.010] | -0.03 \| 0.02 | 0.777 (0.633) |
| OD | 0.010 [0.010] | -0.01 \| 0.04 | 0.934 (0.415) | 0.013 [0.010] | -0.01 \| 0.04 | 0.837 (0.291) |
| **Dorsal cingulum** | | | | | | |
| Overall diffusion | -0.014 [0.010] | -0.03 \| <0.001 | 0.689 (0.141) | -0.006 [0.010] | -0.02 \| 0.01 | 0.863 (0.531) |
| AD | 0.003 [0.010] | -0.02 \| 0.03 | 0.982 (0.792) | 0.011 [0.010] | -0.01 \| 0.04 | 0.754 (0.355) |
| FA | -0.013 [0.010] | -0.04 \| 0.01 | 0.888 (0.296) | -0.005 [0.010] | -0.03 \| 0.02 | 0.948 (0.691) |
| MD | -0.024 [0.010] | -0.05 \| <0.001 | 0.486 (0.054) | -0.016 [0.010] | -0.04 \| 0.01 | 0.777 (0.197) |
| RD | -0.023 [0.010] | -0.05 \| <0.001 | 0.513 (0.067) | -0.015 [0.010] | -0.04 \| 0.01 | 0.758 (0.233) |
| ND | -0.025 [0.010] | -0.05 \| <-0.001 | 0.387 (0.043) | -0.017 [0.010] | -0.04 \| 0.01 | 0.743 (0.165) |
| OD | -0.002 [0.010] | -0.03 \| 0.02 | 0.973 (0.895) | 0.006 [0.010] | -0.02 \| 0.03 | 0.837 (0.600) |
| **Ventral cingulum** | | | | | | |
| Overall diffusion | -0.002 [0.010] | -0.02 \| 0.01 | 0.955 (0.799) | 0.003 [0.010] | -0.01 \| 0.02 | 0.863 (0.726) |
| AD | <0.001 [0.010] | -0.02 \| 0.02 | 0.984 (0.984) | 0.005 [0.010] | -0.02 \| 0.03 | 0.774 (0.688) |
| FA | -0.009 [0.010] | -0.03 \| 0.01 | 0.892 (0.476) | -0.004 [0.010] | -0.03 \| 0.02 | 0.948 (0.737) |
| MD | -0.003 [0.010] | -0.03 \| 0.02 | 0.989 (0.810) | 0.002 [0.010] | -0.02 \| 0.03 | 0.942 (0.888) |
| RD | <0.001 [0.010] | -0.02 \| 0.02 | 0.991 (0.991) | 0.005 [0.010] | -0.02 \| 0.03 | 0.963 (0.694) |
| ND | <0.001 [0.010] | -0.02 \| 0.02 | 0.993 (0.993) | 0.004 [0.010] | -0.02 \| 0.03 | 0.777 (0.709) |
| OD | <0.001 [0.010] | -0.02 \| 0.02 | 0.973 (0.973) | 0.004 [0.010] | -0.02 \| 0.03 | 0.837 (0.728) |
| **Posterior thal rad** | | | | | | |
| Overall diffusion | -0.002 [0.010] | -0.02 \| 0.01 | 0.955 (0.810) | <0.001 [0.010] | -0.02 \| 0.01 | 0.997 (0.997) |
| AD | -0.002 [0.010] | -0.03 \| 0.02 | 0.982 (0.873) | <0.001 [0.010] | -0.02 \| 0.02 | 0.995 (0.995) |
| FA | -0.002 [0.010] | -0.03 \| 0.02 | 0.892 (0.843) | -0.001 [0.010] | -0.03 \| 0.02 | 0.964 (0.964) |
| MD | <0.001 [0.010] | -0.03 \| 0.02 | 0.989 (0.981) | 0.002 [0.010] | -0.02 \| 0.03 | 0.942 (0.896) |
| RD | -0.002 [0.010] | -0.03 \| 0.02 | 0.991 (0.901) | <0.001 [0.010] | -0.02 \| 0.03 | 0.977 (0.977) |
| ND | -0.007 [0.010] | -0.03 \| 0.02 | 0.825 (0.550) | -0.006 [0.010] | -0.03 \| 0.02 | 0.777 (0.654) |
| OD | 0.002 [0.010] | -0.02 \| 0.03 | 0.973 (0.864) | 0.004 [0.010] | -0.02 \| 0.03 | 0.837 (0.744) |
| **Sag Stratum** | | | | | | |
| Overall diffusion | 0.003 [0.010] | -0.01 \| 0.02 | 0.955 (0.749) | 0.003 [0.010] | -0.01 \| 0.02 | 0.863 (0.689) |
| AD | 0.011 [0.010] | -0.01 \| 0.04 | 0.982 (0.401) | 0.011 [0.010] | -0.01 \| 0.04 | 0.754 (0.372) |
| FA | 0.004 [0.010] | -0.02 \| 0.03 | 0.892 (0.766) | 0.004 [0.010] | -0.02 \| 0.03 | 0.948 (0.724) |
| MD | -0.002 [0.010] | -0.03 \| 0.02 | 0.989 (0.896) | -0.001 [0.010] | -0.03 \| 0.02 | 0.942 (0.942) |
| RD | <0.001 [0.010] | -0.03 \| 0.02 | 0.991 (0.987) | 0.001 [0.010] | -0.02 \| 0.03 | 0.977 (0.967) |
| ND | -0.011 [0.010] | -0.04 \| 0.01 | 0.825 (0.398) | -0.010 [0.010] | -0.04 \| 0.01 | 0.777 (0.435) |
| OD | 0.015 [0.010] | -0.01 \| 0.04 | 0.934 (0.245) | 0.015 [0.010] | -0.01 \| 0.04 | 0.837 (0.226) |
| **SLF** | | | | | | |
| Overall diffusion | -0.012 [0.010] | -0.03 \| <0.001 | 0.689 (0.153) | -0.008 [0.010] | -0.02 \| 0.01 | 0.863 (0.353) |
| AD | 0.006 [0.010] | -0.02 \| 0.03 | 0.982 (0.633) | 0.010 [0.010] | -0.01 \| 0.04 | 0.754 (0.419) |
| FA | -0.016 [0.010] | -0.04 \| 0.01 | 0.888 (0.201) | -0.012 [0.010] | -0.04 \| 0.01 | 0.948 (0.345) |
| MD | -0.019 [0.010] | -0.04 \| 0.01 | 0.608 (0.135) | -0.015 [0.010] | -0.04 \| 0.01 | 0.777 (0.246) |
| RD | -0.020 [0.010] | -0.04 \| <0.001 | 0.513 (0.114) | -0.016 [0.010] | -0.04 \| 0.01 | 0.758 (0.212) |
| ND | -0.013 [0.010] | -0.04 \| 0.01 | 0.825 (0.309) | -0.009 [0.010] | -0.03 \| 0.02 | 0.777 (0.494) |
| OD | -0.011 [0.010] | -0.04 \| 0.01 | 0.934 (0.394) | -0.007 [0.010] | -0.03 \| 0.02 | 0.837 (0.604) |
| **uncF** | | | | | | |
| Overall diffusion | 0.005 [0.010] | -0.01 \| 0.02 | 0.955 (0.556) | 0.009 [0.010] | -0.01 \| 0.03 | 0.863 (0.341) |
| AD | 0.003 [0.010] | -0.02 \| 0.03 | 0.982 (0.797) | 0.006 [0.010] | -0.02 \| 0.03 | 0.774 (0.598) |
| FA | 0.015 [0.010] | -0.01 \| 0.04 | 0.888 (0.203) | 0.019 [0.010] | <0.001 \| 0.04 | 0.948 (0.125) |
| MD | -0.002 [0.010] | -0.03 \| 0.02 | 0.989 (0.900) | 0.002 [0.010] | -0.02 \| 0.03 | 0.942 (0.883) |
| RD | 0.010 [0.010] | -0.01 \| 0.03 | 0.977 (0.434) | 0.013 [0.010] | -0.01 \| 0.04 | 0.758 (0.294) |
| ND | 0.002 [0.010] | -0.02 \| 0.03 | 0.988 (0.878) | 0.005 [0.010] | -0.02 \| 0.03 | 0.777 (0.671) |
| OD | 0.003 [0.010] | -0.02 \| 0.03 | 0.973 (0.809) | 0.006 [0.010] | -0.02 \| 0.03 | 0.837 (0.608) |
| False Discovery rate (FDR) significant differences are marked in bold. P<0.05, uncorrected significant results are underlined. *Corrected for age, sex and education in years; Abbreviations: FA = fractional anisotropy, MD = mean diffusivity, RD = radial diffusivity, AD = axial diffusivity, ND = neurite density, OD = orientation dispersion, SLF = superior longitudinal fascicle, CC genu = genu of the corpus callosum, CC body = body of the corpus callosum, CC splenium = splenium of the corpus callosum. | | | | | | |

| **Supplementary Table 9 – Mixed model analyses of white matter microstructure and illness duration – ComBat corrected** | | | | | | | | |
| --- | --- | --- | --- | --- | --- | --- | --- | --- |
|  | **Group difference (crude model)** | | | **Group difference (adjusted model)*** | | | | |
|  | B [SE] | 95% CI | P (unc) | B [SE] | | | 95% CI | P (unc) |
| **CC genu** | | | | | | | | |
| Overall diffusion | -0.008 [<0.001] | -0.01 \| <0.001 | 0.162 (0.023) | 0.001 [<0.001] | | | -0.01 \| 0.01 | 0.816 (0.816) |
| AD | -0.002 [0.010] | -0.01 \| 0.01 | 0.813 (0.668) | 0.006 [0.010] | | | <0.001 \| 0.02 | 0.894 (0.293) |
| FA | -0.015 [0.010] | -0.03 \| <0.001 | 0.063 (0.007) | -0.007 [0.010] | | | -0.02 \| <0.001 | 0.700 (0.257) |
| MD | -0.005 [0.010] | -0.02 \| 0.01 | 0.720 (0.394) | 0.004 [0.010] | | | -0.01 \| 0.01 | 0.858 (0.521) |
| RD | -0.011 [0.010] | -0.02 \| <0.001 | 0.264 (0.041) | -0.003 [0.010] | | | -0.01 \| 0.01 | 0.858 (0.607) |
| ND | -0.004 [0.010] | -0.01 \| 0.01 | 0.607 (0.452) | 0.004 [0.010] | | | -0.01 \| 0.01 | 0.518 (0.460) |
| OD | -0.008 [0.010] | -0.02 \| <0.001 | 0.562 (0.158) | 0.001 [0.010] | | | -0.01 \| 0.01 | 0.919 (0.919) |
| **CC body** | | | | | | | | |
| Overall diffusion | -0.001 [<0.001] | -0.01 \| 0.01 | 0.924 (0.821) | 0.003 [<0.001] | | <0.001 \| 0.01 | | 0.816 (0.411) |
| AD | -0.003 [<0.001] | -0.01 \| 0.01 | 0.813 (0.552) | 0.001 [0.010] | | -0.01 \| 0.01 | | 0.920 (0.920) |
| FA | <0.001 [<0.001] | -0.01 \| 0.01 | 0.978 (0.978) | 0.004 [0.010] | | -0.01 \| 0.01 | | 0.734 (0.489) |
| MD | 0.001 [<0.001] | -0.01 \| 0.01 | 0.887 (0.887) | 0.005 [0.010] | | -0.01 \| 0.02 | | 0.858 (0.424) |
| RD | <0.001 [<0.001] | -0.01 \| 0.01 | 0.975 (0.969) | 0.004 [0.010] | | -0.01 \| 0.01 | | 0.858 (0.482) |
| ND | 0.004 [<0.001] | -0.01 \| 0.01 | 0.607 (0.472) | 0.008 [0.010] | | <0.001 \| 0.02 | | 0.369 (0.178) |
| OD | -0.006 [<0.001] | -0.02 \| <0.001 | 0.562 (0.250) | -0.002 [0.010] | | -0.01 \| 0.01 | | 0.860 (0.669) |
| **CC splenium** | | | | | | | | |
| Overall diffusion | -0.002 [<0.001] | -0.01 \| <0.001 | 0.649 (0.505) | 0.002 [<0.001] | | <0.001 \| 0.01 | | 0.816 (0.552) |
| AD | 0.002 [0.010] | -0.01 \| 0.01 | 0.813 (0.779) | 0.006 [0.010] | | <0.001 \| 0.02 | | 0.894 (0.298) |
| FA | -0.005 [0.010] | -0.02 \| 0.01 | 0.435 (0.338) | -0.001 [0.010] | | -0.01 \| 0.01 | | 0.928 (0.887) |
| MD | -0.006 [0.010] | -0.02 \| <0.001 | 0.720 (0.319) | -0.001 [0.010] | | -0.01 \| 0.01 | | 0.858 (0.858) |
| RD | -0.005 [0.010] | -0.02 \| 0.01 | 0.517 (0.345) | -0.001 [0.010] | | -0.01 \| 0.01 | | 0.897 (0.897) |
| ND | <0.001 [0.010] | -0.01 \| 0.01 | 1.000 (1.000) | 0.005 [0.010] | | -0.01 \| 0.02 | | 0.518 (0.440) |
| OD | 0.001 [0.010] | -0.01 \| 0.01 | 0.848 (0.848) | | 0.006 [0.010] | -0.01 \| 0.02 | | 0.860 (0.339) |
| **Dorsal cingulum** | | | | | | | | |
| Overall diffusion | 0.005 [<0.001] | <0.001 \| 0.01 | 0.350 (0.233) | | 0.005 [<0.001] | <0.001 \| 0.01 | | 0.816 (0.268) |
| AD | 0.003 [0.010] | -0.01 \| 0.01 | 0.813 (0.640) | | 0.003 [0.010] | -0.01 \| 0.01 | | 0.920 (0.643) |
| FA | 0.006 [0.010] | <0.001 \| 0.02 | 0.420 (0.280) | | 0.006 [0.010] | <0.001 \| 0.02 | | 0.700 (0.298) |
| MD | 0.003 [0.010] | -0.01 \| 0.01 | 0.720 (0.640) | | 0.003 [0.010] | -0.01 \| 0.01 | | 0.858 (0.643) |
| RD | 0.006 [0.010] | <0.001 \| 0.02 | 0.517 (0.311) | | 0.006 [0.010] | -0.01 \| 0.02 | | 0.858 (0.328) |
| ND | 0.010 [0.010] | <0.001 \| 0.02 | 0.173 (0.077) | | 0.010 [0.010] | <0.001 \| 0.02 | | 0.273 (0.091) |
| OD | 0.003 [0.010] | -0.01 \| 0.01 | 0.804 (0.536) | | 0.004 [0.010] | -0.01 \| 0.01 | | 0.860 (0.544) |
| **Ventral cingulum** | | | | | | | | |
| Overall diffusion | 0.005 [<0.001] | <0.001 \| 0.01 | 0.350 (0.159) | | -0.001 [<0.001] | | -0.01 \| 0.01 | 0.816 (0.732) |
| AD | -0.001 [<0.001] | -0.01 \| 0.01 | 0.813 (0.813) | | -0.007 [0.010] | | -0.02 \| <0.001 | 0.894 (0.191) |
| FA | 0.007 [<0.001] | <0.001 \| 0.02 | 0.394 (0.219) | | 0.001 [0.010] | | -0.01 \| 0.01 | 0.928 (0.928) |
| MD | 0.003 [<0.001] | -0.01 \| 0.01 | 0.720 (0.524) | | -0.003 [0.010] | | -0.01 \| 0.01 | 0.858 (0.635) |
| RD | 0.004 [<0.001] | -0.01 \| 0.01 | 0.531 (0.413) | | -0.002 [0.010] | | -0.01 \| 0.01 | 0.858 (0.763) |
| ND | 0.013 [<0.001] | <0.001 \| 0.02 | 0.063 (0.014) | | 0.007 [0.010] | | <0.001 \| 0.02 | 0.369 (0.205) |
| OD | 0.002 [<0.001] | -0.01 \| 0.01 | 0.846 (0.658) | | -0.004 [0.010] | | -0.01 \| 0.01 | 0.860 (0.509) |
| **Posterior thal rad** | | | | | | | | |
| Overall diffusion | -0.008 [<0.001] | -0.01 \| <0.001 | 0.162 (0.036) | | -0.001 [<0.001] | | -0.01 \| 0.01 | 0.816 (0.789) |
| AD | -0.004 [0.010] | -0.01 \| 0.01 | 0.813 (0.466) | | 0.002 [0.010] | | -0.01 \| 0.01 | 0.920 (0.679) |
| FA | -0.011 [0.010] | -0.02 \| <0.001 | 0.193 (0.043) | | -0.005 [0.010] | | -0.02 \| 0.01 | 0.734 (0.415) |
| MD | -0.005 [0.010] | -0.02 \| 0.01 | 0.720 (0.345) | | 0.001 [0.010] | | -0.01 \| 0.01 | 0.858 (0.834) |
| RD | -0.010 [0.010] | -0.02 \| <0.001 | 0.264 (0.067) | | -0.004 [0.010] | | -0.01 \| 0.01 | 0.858 (0.525) |
| ND | -0.005 [0.010] | -0.02 \| 0.01 | 0.607 (0.379) | | 0.002 [0.010] | | -0.01 \| 0.01 | 0.786 (0.786) |
| OD | -0.010 [0.010] | -0.02 \| <0.001 | 0.562 (0.082) | | -0.003 [0.010] | | -0.01 \| 0.01 | 0.860 (0.586) |
| **Sag Stratum** | | | | | | | | |
| Overall diffusion | -0.005 [<0.001] | -0.01 \| <0.001 | 0.350 (0.204) | -0.002 [<0.001] | | -0.01 \| 0.01 | | 0.816 (0.612) |
| AD | -0.004 [0.010] | -0.01 \| 0.01 | 0.813 (0.435) | -0.002 [0.010] | | -0.01 \| 0.01 | | 0.920 (0.770) |
| FA | -0.009 [0.010] | -0.02 \| <0.001 | 0.360 (0.120) | -0.006 [0.010] | | -0.02 \| 0.01 | | 0.700 (0.311) |
| MD | -0.004 [0.010] | -0.01 \| 0.01 | 0.720 (0.498) | -0.001 [0.010] | | -0.01 \| 0.01 | | 0.858 (0.844) |
| RD | -0.007 [0.010] | -0.02 \| <0.001 | 0.448 (0.199) | -0.005 [0.010] | | -0.02 \| 0.01 | | 0.858 (0.445) |
| ND | 0.002 [0.010] | -0.01 \| 0.01 | 0.774 (0.688) | 0.005 [0.010] | | -0.01 \| 0.02 | | 0.518 (0.418) |
| OD | -0.007 [0.010] | -0.02 \| <0.001 | 0.562 (0.199) | -0.005 [0.010] | | -0.02 \| 0.01 | | 0.860 (0.446) |
| **SLF** | | | | | | | | |
| Overall diffusion | <0.001 [<0.001] | -0.01 \| 0.01 | 0.940 (0.940) | 0.003 [<0.001] | | | -0.01 \| 0.01 | 0.816 (0.556) |
| AD | -0.003 [0.010] | -0.01 \| 0.01 | 0.813 (0.548) | -0.001 [0.010] | | | -0.01 \| 0.01 | 0.920 (0.848) |
| FA | -0.004 [0.010] | -0.01 \| 0.01 | 0.584 (0.519) | -0.001 [0.010] | | | -0.01 \| 0.01 | 0.928 (0.816) |
| MD | 0.003 [0.010] | -0.01 \| 0.01 | 0.720 (0.595) | 0.005 [0.010] | | | -0.01 \| 0.02 | 0.858 (0.381) |
| RD | <0.001 [0.010] | -0.01 \| 0.01 | 0.975 (0.975) | 0.002 [0.010] | | | -0.01 \| 0.01 | 0.858 (0.730) |
| ND | 0.011 [0.010] | <0.001 \| 0.02 | 0.132 (0.044) | 0.014 [0.010] | | | <0.001 \| 0.03 | 0.104 (0.023) |
| OD | -0.005 [0.010] | -0.02 \| 0.01 | 0.599 (0.333) | -0.003 [0.010] | | | -0.01 \| 0.01 | 0.860 (0.592) |
| **uncF** | | | | | | | | |
| Overall diffusion | 0.007 [<0.001] | <0.001 \| 0.01 | 0.294 (0.098) | 0.006 [<0.001] | | | <0.001 \| 0.01 | 0.816 (0.162) |
| AD | -0.003 [<0.001] | -0.01 \| 0.01 | 0.813 (0.565) | -0.003 [0.010] | | | -0.01 \| 0.01 | 0.920 (0.572) |
| FA | 0.008 [<0.001] | <0.001 \| 0.02 | 0.364 (0.162) | 0.007 [0.010] | | | <0.001 \| 0.02 | 0.700 (0.208) |
| MD | 0.009 [<0.001] | <0.001 \| 0.02 | 0.720 (0.098) | 0.009 [0.010] | | | <0.001 \| 0.02 | 0.858 (0.134) |
| RD | 0.009 [<0.001] | <0.001 \| 0.02 | 0.264 (0.088) | 0.009 [0.010] | | | <0.001 \| 0.02 | 0.858 (0.122) |
| ND | 0.018 [<0.001] | 0.01 \| 0.03 | **0.009** (0.001) | 0.018 [0.010] | | | 0.01 \| 0.03 | **0.027** (0.003) |
| OD | -0.001 [<0.001] | -0.01 \| 0.01 | 0.848 (0.834) | -0.001 [0.010] | | | -0.01 \| 0.01 | 0.919 (0.821) |
| False Discovery rate (FDR) significant differences are marked in bold. P<0.05, uncorrected significant results are underlined. *Corrected for age, sex and education in years; Abbreviations: FA = fractional anisotropy, MD = mean diffusivity, RD = radial diffusivity, AD = axial diffusivity, ND = neurite density, OD = orientation dispersion, SLF = superior longitudinal fascicle, CC genu = genu of the corpus callosum, CC body = body of the corpus callosum, CC splenium = splenium of the corpus callosum. | | | | | | | | |

| **Supplementary Table 10 – Mixed model analyses of white matter microstructure and age of onset as continuous measure – ComBat corrected** | | | | | | |
| --- | --- | --- | --- | --- | --- | --- |
|  | **Group difference (crude model)** | | | **Group difference (adjusted model)*** | | |
|  | B [SE] | 95% CI | P (unc) | B [SE] | 95% CI | P (unc) |
| **CC genu** | | | | | | |
| Overall diffusion | -0.004 [<0.001] | -0.01 \| <0.001 | 0.477 (0.265) | <0.001 [<0.001] | -0.01 \| 0.01 | 0.996 (0.996) |
| AD | 0.003 [0.010] | -0.01 \| 0.01 | 0.761 (0.676) | 0.007 [0.010] | <0.001 \| 0.02 | 0.588 (0.271) |
| FA | -0.005 [0.010] | -0.02 \| 0.01 | 0.624 (0.372) | -0.001 [0.010] | -0.01 \| 0.01 | 0.966 (0.826) |
| MD | -0.004 [0.010] | -0.02 \| 0.01 | 0.696 (0.464) | <0.001 [0.010] | -0.01 \| 0.01 | 0.953 (0.953) |
| RD | -0.005 [0.010] | -0.02 \| 0.01 | 0.585 (0.390) | -0.001 [0.010] | -0.01 \| 0.01 | 0.851 (0.851) |
| ND | -0.007 [0.010] | -0.02 \| <0.001 | 0.506 (0.253) | -0.003 [0.010] | -0.01 \| 0.01 | 0.775 (0.636) |
| OD | -0.005 [0.010] | -0.02 \| 0.01 | 0.832 (0.415) | -0.001 [0.010] | -0.01 \| 0.01 | 0.950 (0.887) |
| **CC body** | | | | | | |
| Overall diffusion | -0.002 [<0.001] | -0.01 \| <0.001 | 0.588 (0.523) | -0.001 [<0.001] | -0.01 \| 0.01 | 0.948 (0.826) |
| AD | 0.001 [0.010] | -0.01 \| 0.01 | 0.908 (0.908) | 0.002 [0.010] | -0.01 \| 0.01 | 0.810 (0.720) |
| FA | <0.001 [0.010] | -0.01 \| 0.01 | 0.984 (0.945) | 0.001 [0.010] | -0.01 \| 0.01 | 0.966 (0.859) |
| MD | -0.003 [0.010] | -0.01 \| 0.01 | 0.856 (0.666) | -0.001 [0.010] | -0.01 \| 0.01 | 0.953 (0.856) |
| RD | <0.001 [0.010] | -0.01 \| 0.01 | 0.985 (0.985) | 0.001 [0.010] | -0.01 \| 0.01 | 0.851 (0.821) |
| ND | -0.004 [0.010] | -0.02 \| 0.01 | 0.643 (0.505) | -0.003 [0.010] | -0.01 \| 0.01 | 0.775 (0.679) |
| OD | -0.007 [0.010] | -0.02 \| <0.001 | 0.651 (0.217) | -0.006 [0.010] | -0.02 \| 0.01 | 0.950 (0.328) |
| **CC splenium** | | | | | | |
| Overall diffusion | -0.004 [<0.001] | -0.01 \| <0.001 | 0.477 (0.241) | -0.003 [<0.001] | -0.01 \| <0.001 | 0.948 (0.497) |
| AD | 0.005 [0.010] | -0.01 \| 0.02 | 0.761 (0.411) | 0.007 [0.010] | <0.001 \| 0.02 | 0.588 (0.266) |
| FA | -0.007 [0.010] | -0.02 \| <0.001 | 0.601 (0.267) | -0.005 [0.010] | -0.02 \| 0.01 | 0.938 (0.417) |
| MD | -0.011 [0.010] | -0.02 \| <0.001 | 0.351 (0.085) | -0.009 [0.010] | -0.02 \| <0.001 | 0.698 (0.155) |
| RD | -0.009 [0.010] | -0.02 \| <0.001 | 0.272 (0.121) | -0.008 [0.010] | -0.02 \| <0.001 | 0.475 (0.211) |
| ND | -0.004 [0.010] | -0.02 \| 0.01 | 0.643 (0.486) | -0.002 [0.010] | -0.01 \| 0.01 | 0.775 (0.689) |
| OD | <0.001 [0.010] | -0.01 \| 0.01 | 0.987 (0.987) | 0.002 [0.010] | -0.01 \| 0.01 | 0.950 (0.782) |
| **Dorsal cingulum** | | | | | | |
| Overall diffusion | -0.001 [<0.001] | -0.01 \| 0.01 | 0.777 (0.777) | -0.001 [<0.001] | -0.01 \| 0.01 | 0.948 (0.800) |
| AD | 0.005 [0.010] | -0.01 \| 0.02 | 0.761 (0.406) | 0.005 [0.010] | -0.01 \| 0.02 | 0.588 (0.392) |
| FA | <0.001 [0.010] | -0.01 \| 0.01 | 0.984 (0.984) | <0.001 [0.010] | -0.01 \| 0.01 | 0.997 (0.997) |
| MD | -0.007 [0.010] | -0.02 \| <0.001 | 0.482 (0.247) | -0.007 [0.010] | -0.02 \| <0.001 | 0.765 (0.255) |
| RD | -0.003 [0.010] | -0.01 \| 0.01 | 0.842 (0.655) | -0.003 [0.010] | -0.01 \| 0.01 | 0.851 (0.670) |
| ND | -0.003 [0.010] | -0.01 \| 0.01 | 0.643 (0.593) | -0.003 [0.010] | -0.01 \| 0.01 | 0.775 (0.608) |
| OD | <0.001 [0.010] | -0.01 \| 0.01 | 0.987 (0.968) | <0.001 [0.010] | -0.01 \| 0.01 | 0.950 (0.950) |
| **Ventral cingulum** | | | | | | |
| Overall diffusion | 0.005 [<0.001] | <0.001 \| 0.01 | 0.477 (0.163) | 0.002 [<0.001] | <0.001 \| 0.01 | 0.948 (0.543) |
| AD | -0.002 [0.010] | -0.01 \| 0.01 | 0.761 (0.674) | -0.005 [0.010] | -0.02 \| 0.01 | 0.588 (0.360) |
| FA | 0.008 [0.010] | <0.001 \| 0.02 | 0.528 (0.176) | 0.005 [0.010] | -0.01 \| 0.02 | 0.938 (0.392) |
| MD | 0.007 [0.010] | <0.001 \| 0.02 | 0.482 (0.268) | 0.004 [0.010] | -0.01 \| 0.01 | 0.953 (0.542) |
| RD | 0.010 [0.010] | <0.001 \| 0.02 | 0.234 (0.078) | 0.007 [0.010] | <0.001 \| 0.02 | 0.475 (0.207) |
| ND | 0.007 [0.010] | <0.001 \| 0.02 | 0.506 (0.267) | 0.004 [0.010] | -0.01 \| 0.01 | 0.775 (0.541) |
| OD | 0.002 [0.010] | -0.01 \| 0.01 | 0.987 (0.696) | -0.001 [0.010] | -0.01 \| 0.01 | 0.950 (0.916) |
| **Post Thal Rad** | | | | | | |
| Overall diffusion | -0.009 [<0.001] | -0.02 \| <0.001 | 0.122 (0.016) | -0.005 [<0.001] | -0.01 \| <0.001 | 0.774 (0.172) |
| AD | -0.003 [0.010] | -0.01 \| 0.01 | 0.761 (0.585) | 0.001 [0.010] | -0.01 \| 0.01 | 0.902 (0.902) |
| FA | -0.013 [0.010] | -0.03 \| <0.001 | 0.144 (0.032) | -0.009 [0.010] | -0.02 \| <0.001 | 0.634 (0.141) |
| MD | -0.010 [0.010] | -0.02 \| <0.001 | 0.351 (0.117) | -0.005 [0.010] | -0.02 \| 0.01 | 0.833 (0.370) |
| RD | -0.013 [0.010] | -0.03 \| <0.001 | 0.234 (0.034) | -0.009 [0.010] | -0.02 \| <0.001 | 0.475 (0.149) |
| ND | -0.009 [0.010] | -0.02 \| <0.001 | 0.506 (0.149) | -0.005 [0.010] | -0.02 \| 0.01 | 0.775 (0.441) |
| OD | -0.009 [0.010] | -0.02 \| <0.001 | 0.585 (0.130) | -0.005 [0.010] | -0.02 \| 0.01 | 0.950 (0.400) |
| **Sag Stratum** | | | | | | |
| Overall diffusion | -0.003 [<0.001] | -0.01 \| <0.001 | 0.588 (0.470) | -0.001 [<0.001] | -0.01 \| 0.01 | 0.948 (0.843) |
| AD | -0.005 [0.010] | -0.02 \| 0.01 | 0.761 (0.448) | -0.002 [0.010] | -0.01 \| 0.01 | 0.810 (0.689) |
| FA | -0.005 [0.010] | -0.02 \| 0.01 | 0.624 (0.436) | -0.003 [0.010] | -0.01 \| 0.01 | 0.966 (0.674) |
| MD | -0.001 [0.010] | -0.01 \| 0.01 | 0.912 (0.912) | 0.001 [0.010] | -0.01 \| 0.01 | 0.953 (0.812) |
| RD | -0.001 [0.010] | -0.01 \| 0.01 | 0.985 (0.921) | 0.002 [0.010] | -0.01 \| 0.01 | 0.851 (0.804) |
| ND | -0.003 [0.010] | -0.01 \| 0.01 | 0.643 (0.643) | -0.001 [0.010] | -0.01 \| 0.01 | 0.912 (0.912) |
| OD | -0.005 [0.010] | -0.02 \| 0.01 | 0.832 (0.462) | -0.002 [0.010] | -0.01 \| 0.01 | 0.950 (0.706) |
| **SLF** | | | | | | |
| Overall diffusion | -0.003 [<0.001] | -0.01 \| <0.001 | 0.588 (0.443) | -0.002 [<0.001] | -0.01 \| 0.01 | 0.948 (0.619) |
| AD | 0.009 [0.010] | <0.001 \| 0.02 | 0.621 (0.138) | 0.010 [0.010] | <0.001 \| 0.02 | 0.588 (0.101) |
| FA | -0.004 [0.010] | -0.02 \| 0.01 | 0.624 (0.485) | -0.003 [0.010] | -0.01 \| 0.01 | 0.966 (0.604) |
| MD | -0.011 [0.010] | -0.02 \| <0.001 | 0.351 (0.067) | -0.010 [0.010] | -0.02 \| <0.001 | 0.698 (0.101) |
| RD | -0.008 [0.010] | -0.02 \| <0.001 | 0.391 (0.217) | -0.006 [0.010] | -0.02 \| 0.01 | 0.529 (0.294) |
| ND | -0.007 [0.010] | -0.02 \| <0.001 | 0.506 (0.281) | -0.006 [0.010] | -0.02 \| 0.01 | 0.775 (0.371) |
| OD | 0.002 [0.010] | -0.01 \| 0.01 | 0.987 (0.795) | 0.003 [0.010] | -0.01 \| 0.01 | 0.950 (0.667) |
| **uncF** | | | | | | |
| Overall diffusion | 0.010 [<0.001] | <0.001 \| 0.02 | 0.122 (0.027) | 0.009 [<0.001] | <0.001 \| 0.02 | 0.378 (0.042) |
| AD | 0.009 [0.010] | <0.001 \| 0.02 | 0.621 (0.130) | 0.009 [0.010] | <0.001 \| 0.02 | 0.588 (0.158) |
| FA | 0.014 [0.010] | <0.001 \| 0.03 | 0.135 (0.015) | 0.014 [0.010] | <0.001 \| 0.03 | 0.189 (0.021) |
| MD | 0.001 [0.010] | -0.01 \| 0.01 | 0.912 (0.847) | 0.001 [0.010] | -0.01 \| 0.01 | 0.953 (0.908) |
| RD | 0.011 [0.010] | <0.001 \| 0.02 | 0.234 (0.067) | 0.010 [0.010] | <0.001 \| 0.02 | 0.475 (0.084) |
| ND | 0.009 [0.010] | <0.001 \| 0.02 | 0.506 (0.139) | 0.008 [0.010] | <0.001 \| 0.02 | 0.775 (0.168) |
| OD | 0.013 [0.010] | <0.001 \| 0.03 | 0.234 (0.026) | 0.013 [0.010] | <0.001 \| 0.02 | 0.306 (0.034) |
| False Discovery rate (FDR) significant differences are marked in bold. P<0.05, uncorrected significant results are underlined. *Corrected for age, sex and education in years; Abbreviations: FA = fractional anisotropy, MD = mean diffusivity, RD = radial diffusivity, AD = axial diffusivity, ND = neurite density, OD = orientation dispersion, SLF = superior longitudinal fascicle, CC genu = genu of the corpus callosum, CC body = body of the corpus callosum, CC splenium = splenium of the corpus callosum. | | | | | | |

| **Supplementary Table 11 – Associations between tensor/noddi measures and D-YBOCS subscales** | | | | | | | |
| --- | --- | --- | --- | --- | --- | --- | --- |
|  |  | **crude model** | | | **adjusted model*** | | |
|  | subscale | B [SE]  (x10^-2^) | 95% CI  (x10^-2^) | P (unc) | B [SE]  (x10^-2^) | 95% CI  (x10^-2^) | P (unc) |
| **CC genu** | | | | | | | |
| AD | aggressive | 0.100 [0.100] | -0.2 \| 0.3 | 0.684 | 0.100 [0.100] | -0.2 \| 0.3 | 0.640 |
|  | sexual | 0.100 [0.100] | -0.2 \| 0.3 | 0.619 | <0.001 [0.100] | -0.2 \| 0.3 | 0.744 |
|  | symmetry | -0.200 [0.100] | -0.4 \| <0.001 | 0.110 | -0.200 [0.100] | -0.4 \| <0.001 | 0.102 |
|  | contamination | <0.001 [0.100] | -0.2 \| 0.2 | 0.994 | <0.001 [0.100] | -0.2 \| 0.3 | 0.843 |
|  | hoarding | 0.200 [0.200] | -0.2 \| 0.7 | 0.368 | 0.300 [0.200] | -0.1 \| 0.8 | 0.160 |
| FA | aggressive | 0.100 [0.100] | -0.1 \| 0.2 | 0.543 | <0.001 [0.100] | -0.1 \| 0.2 | 0.618 |
|  | sexual | 0.100 [0.100] | -0.1 \| 0.3 | 0.156 | 0.100 [0.100] | -0.1 \| 0.3 | 0.313 |
|  | symmetry | -0.100 [0.100] | -0.2 \| 0.1 | 0.403 | -0.100 [0.100] | -0.2 \| 0.1 | 0.361 |
|  | contamination | <0.001 [0.100] | -0.2 \| 0.1 | 0.856 | <0.001 [0.100] | -0.2 \| 0.2 | 0.957 |
|  | hoarding | <0.001 [0.200] | -0.3 \| 0.3 | 0.977 | 0.200 [0.200] | -0.1 \| 0.5 | 0.115 |
| MD | aggressive | <0.001 [0.100] | -0.2 \| 0.1 | 0.649 | <0.001 [0.100] | -0.2 \| 0.1 | 0.729 |
|  | sexual | -0.100 [0.100] | -0.3 \| 0.1 | 0.518 | <0.001 [0.100] | -0.2 \| 0.2 | 0.635 |
|  | symmetry | <0.001 [0.100] | -0.2 \| 0.1 | 0.705 | <0.001 [0.100] | -0.2 \| 0.1 | 0.699 |
|  | contamination | 0.100 [0.100] | -0.1 \| 0.2 | 0.562 | 0.100 [0.100] | -0.1 \| 0.2 | 0.544 |
|  | hoarding | 0.100 [0.200] | -0.2 \| 0.4 | 0.438 | <0.001 [0.200] | -0.3 \| 0.4 | 0.891 |
| RD | aggressive | -0.100 [0.100] | -0.3 \| 0.2 | 0.568 | -0.100 [0.100] | -0.3 \| 0.2 | 0.644 |
|  | sexual | -0.200 [0.100] | -0.4 \| 0.1 | 0.201 | -0.100 [0.100] | -0.3 \| 0.1 | 0.344 |
|  | symmetry | <0.001 [0.100] | -0.2 \| 0.3 | 0.706 | <0.001 [0.100] | -0.2 \| 0.2 | 0.691 |
|  | contamination | 0.100 [0.100] | -0.2 \| 0.3 | 0.631 | <0.001 [0.100] | -0.2 \| 0.3 | 0.676 |
|  | hoarding | <0.001 [0.200] | -0.4 \| 0.4 | 0.857 | -0.200 [0.200] | -0.6 \| 0.2 | 0.276 |
| ND | aggressive | 0.100 [0.100] | -0.1 \| 0.3 | 0.272 | 0.100 [0.100] | -0.1 \| 0.3 | 0.287 |
|  | sexual | 0.200 [0.100] | <0.001 \| 0.4 | 0.131 | 0.100 [0.100] | -0.1 \| 0.4 | 0.188 |
|  | symmetry | 0.100 [0.100] | -0.1 \| 0.2 | 0.531 | 0.100 [0.100] | -0.1 \| 0.2 | 0.534 |
|  | contamination | -0.100 [0.100] | -0.3 \| 0.1 | 0.294 | -0.100 [0.100] | -0.3 \| 0.1 | 0.339 |
|  | hoarding | -0.200 [0.200] | -0.5 \| 0.2 | 0.315 | -0.100 [0.200] | -0.4 \| 0.3 | 0.760 |
| OD | aggressive | <0.001 [<0.001] | -0.1 \| <0.001 | 0.651 | <0.001 [<0.001] | -0.1 \| <0.001 | 0.649 |
|  | sexual | <0.001 [<0.001] | -0.1 \| <0.001 | 0.302 | <0.001 [<0.001] | -0.1 \| <0.001 | 0.483 |
|  | symmetry | 0.100 [<0.001] | <0.001 \| 0.1 | 0.022 | 0.100 [<0.001] | <0.001 \| 0.1 | 0.017 |
|  | contamination | <0.001 [<0.001] | -0.1 \| <0.001 | 0.791 | <0.001 [<0.001] | -0.1 \| <0.001 | 0.626 |
|  | hoarding | <0.001 [<0.001] | -0.1 \| 0.1 | 0.533 | -0.100 [<0.001] | -0.2 \| <0.001 | 0.075 |
| **CC body** | | | | | | | |
| AD | aggressive | 0.100 [0.100] | -0.1 \| 0.3 | 0.348 | 0.100 [0.100] | -0.1 \| 0.4 | 0.298 |
|  | sexual | <0.001 [0.100] | -0.3 \| 0.2 | 0.745 | -0.100 [0.100] | -0.3 \| 0.2 | 0.679 |
|  | symmetry | -0.100 [0.100] | -0.3 \| 0.1 | 0.411 | -0.100 [0.100] | -0.3 \| 0.1 | 0.438 |
|  | contamination | 0.100 [0.100] | -0.1 \| 0.3 | 0.392 | 0.100 [0.100] | -0.1 \| 0.3 | 0.309 |
|  | hoarding | 0.100 [0.200] | -0.4 \| 0.5 | 0.783 | 0.100 [0.200] | -0.3 \| 0.6 | 0.502 |
| FA | aggressive | 0.200 [0.100] | <0.001 \| 0.3 | 0.122 | 0.200 [0.100] | <0.001 \| 0.3 | 0.124 |
|  | sexual | 0.100 [0.100] | -0.1 \| 0.3 | 0.518 | 0.100 [0.100] | -0.2 \| 0.3 | 0.579 |
|  | symmetry | 0.100 [0.100] | -0.1 \| 0.2 | 0.472 | 0.100 [0.100] | -0.1 \| 0.3 | 0.469 |
|  | contamination | -0.100 [0.100] | -0.3 \| 0.1 | 0.326 | -0.100 [0.100] | -0.3 \| 0.1 | 0.366 |
|  | hoarding | <0.001 [0.200] | -0.4 \| 0.3 | 0.830 | <0.001 [0.200] | -0.3 \| 0.4 | 0.911 |
| MD | aggressive | <0.001 [0.100] | -0.2 \| 0.1 | 0.629 | <0.001 [0.100] | -0.2 \| 0.1 | 0.667 |
|  | sexual | -0.100 [0.100] | -0.2 \| 0.1 | 0.507 | -0.100 [0.100] | -0.2 \| 0.1 | 0.537 |
|  | symmetry | -0.100 [0.100] | -0.3 \| <0.001 | 0.149 | -0.100 [0.100] | -0.3 \| <0.001 | 0.159 |
|  | contamination | 0.100 [0.100] | -0.1 \| 0.3 | 0.261 | 0.100 [0.100] | -0.1 \| 0.3 | 0.262 |
|  | hoarding | 0.100 [0.200] | -0.2 \| 0.4 | 0.404 | 0.100 [0.200] | -0.2 \| 0.4 | 0.479 |
| RD | aggressive | -0.100 [0.100] | -0.4 \| 0.1 | 0.257 | -0.100 [0.100] | -0.4 \| 0.1 | 0.270 |
|  | sexual | -0.100 [0.100] | -0.4 \| 0.2 | 0.469 | -0.100 [0.100] | -0.4 \| 0.2 | 0.517 |
|  | symmetry | -0.100 [0.100] | -0.3 \| 0.1 | 0.402 | -0.100 [0.100] | -0.3 \| 0.1 | 0.406 |
|  | contamination | 0.100 [0.100] | -0.1 \| 0.3 | 0.323 | 0.100 [0.100] | -0.1 \| 0.3 | 0.345 |
|  | hoarding | 0.100 [0.200] | -0.3 \| 0.5 | 0.696 | <0.001 [0.200] | -0.4 \| 0.5 | 0.895 |
| ND | aggressive | 0.100 [0.100] | -0.1 \| 0.3 | 0.273 | 0.100 [0.100] | -0.1 \| 0.3 | 0.245 |
|  | sexual | 0.100 [0.100] | -0.1 \| 0.3 | 0.161 | 0.100 [0.100] | -0.1 \| 0.3 | 0.179 |
|  | symmetry | <0.001 [0.100] | -0.1 \| 0.2 | 0.568 | <0.001 [0.100] | -0.1 \| 0.2 | 0.561 |
|  | contamination | -0.100 [0.100] | -0.3 \| <0.001 | 0.092 | -0.100 [0.100] | -0.3 \| <0.001 | 0.125 |
|  | hoarding | -0.100 [0.100] | -0.4 \| 0.2 | 0.580 | -0.100 [0.200] | -0.4 \| 0.3 | 0.733 |
| OD | aggressive | <0.001 [<0.001] | -0.1 \| <0.001 | 0.071 | <0.001 [<0.001] | -0.1 \| <0.001 | 0.064 |
|  | sexual | <0.001 [<0.001] | -0.1 \| <0.001 | 0.755 | <0.001 [<0.001] | <0.001 \| <0.001 | 0.936 |
|  | symmetry | <0.001 [<0.001] | <0.001 \| <0.001 | 0.736 | <0.001 [<0.001] | <0.001 \| <0.001 | 0.750 |
|  | contamination | <0.001 [<0.001] | -0.1 \| <0.001 | 0.490 | <0.001 [<0.001] | -0.1 \| <0.001 | 0.383 |
|  | hoarding | <0.001 [<0.001] | -0.1 \| 0.1 | 0.925 | <0.001 [<0.001] | -0.1 \| <0.001 | 0.344 |
| **CC splenium** | | | | | | | |
| AD | aggressive | <0.001 [0.100] | -0.2 \| 0.3 | 0.714 | 0.100 [0.100] | -0.1 \| 0.4 | 0.371 |
|  | sexual | 0.100 [0.100] | -0.2 \| 0.4 | 0.400 | 0.100 [0.100] | -0.2 \| 0.4 | 0.428 |
|  | symmetry | -0.100 [0.100] | -0.4 \| 0.1 | 0.337 | -0.100 [0.100] | -0.3 \| 0.1 | 0.428 |
|  | contamination | 0.100 [0.100] | -0.2 \| 0.3 | 0.445 | 0.200 [0.100] | -0.1 \| 0.4 | 0.171 |
|  | hoarding | -0.100 [0.200] | -0.6 \| 0.3 | 0.577 | <0.001 [0.200] | -0.4 \| 0.5 | 0.881 |
| FA | aggressive | 0.100 [0.100] | <0.001 \| 0.2 | 0.118 | 0.100 [0.100] | <0.001 \| 0.2 | 0.098 |
|  | sexual | <0.001 [0.100] | -0.1 \| 0.2 | 0.463 | <0.001 [0.100] | -0.1 \| 0.1 | 0.633 |
|  | symmetry | <0.001 [0.100] | -0.1 \| 0.1 | 0.837 | <0.001 [0.100] | -0.1 \| 0.1 | 0.811 |
|  | contamination | -0.100 [0.100] | -0.2 \| <0.001 | 0.111 | -0.100 [0.100] | -0.2 \| <0.001 | 0.174 |
|  | hoarding | -0.100 [0.100] | -0.2 \| 0.1 | 0.526 | <0.001 [0.100] | -0.2 \| 0.2 | 0.715 |
| MD | aggressive | <0.001 [0.100] | -0.2 \| 0.1 | 0.540 | <0.001 [0.100] | -0.1 \| 0.1 | 0.831 |
|  | sexual | <0.001 [0.100] | -0.2 \| 0.1 | 0.866 | <0.001 [0.100] | -0.1 \| 0.1 | 0.976 |
|  | symmetry | -0.100 [0.100] | -0.2 \| <0.001 | 0.191 | -0.100 [0.100] | -0.2 \| <0.001 | 0.243 |
|  | contamination | 0.100 [0.100] | <0.001 \| 0.2 | 0.087 | 0.100 [0.100] | <0.001 \| 0.3 | 0.040 |
|  | hoarding | <0.001 [0.100] | -0.2 \| 0.3 | 0.715 | <0.001 [0.100] | -0.2 \| 0.3 | 0.840 |
| RD | aggressive | -0.100 [0.100] | -0.2 \| <0.001 | 0.132 | -0.100 [0.100] | -0.2 \| <0.001 | 0.149 |
|  | sexual | -0.100 [0.100] | -0.2 \| 0.1 | 0.500 | <0.001 [0.100] | -0.2 \| 0.1 | 0.684 |
|  | symmetry | <0.001 [0.100] | -0.2 \| 0.1 | 0.567 | <0.001 [0.100] | -0.2 \| 0.1 | 0.576 |
|  | contamination | 0.100 [0.100] | <0.001 \| 0.3 | 0.072 | 0.100 [0.100] | <0.001 \| 0.2 | 0.087 |
|  | hoarding | 0.100 [0.100] | -0.2 \| 0.3 | 0.555 | <0.001 [0.100] | -0.3 \| 0.2 | 0.738 |
| ND | aggressive | 0.100 [0.100] | <0.001 \| 0.3 | 0.174 | 0.100 [0.100] | -0.1 \| 0.3 | 0.204 |
|  | sexual | 0.100 [0.100] | -0.1 \| 0.2 | 0.439 | 0.100 [0.100] | -0.1 \| 0.2 | 0.483 |
|  | symmetry | 0.100 [0.100] | -0.1 \| 0.2 | 0.305 | 0.100 [0.100] | -0.1 \| 0.2 | 0.316 |
|  | contamination | -0.100 [0.100] | -0.2 \| <0.001 | 0.180 | -0.100 [0.100] | -0.3 \| <0.001 | 0.175 |
|  | hoarding | -0.200 [0.100] | -0.4 \| 0.1 | 0.217 | -0.100 [0.100] | -0.4 \| 0.1 | 0.327 |
| OD | aggressive | <0.001 [<0.001] | -0.1 \| <0.001 | 0.317 | <0.001 [<0.001] | -0.1 \| <0.001 | 0.108 |
|  | sexual | <0.001 [<0.001] | -0.1 \| <0.001 | 0.784 | <0.001 [<0.001] | -0.1 \| <0.001 | 0.857 |
|  | symmetry | <0.001 [<0.001] | <0.001 \| 0.1 | 0.381 | <0.001 [<0.001] | <0.001 \| 0.1 | 0.488 |
|  | contamination | <0.001 [<0.001] | <0.001 \| 0.1 | 0.688 | <0.001 [<0.001] | -0.1 \| <0.001 | 0.814 |
|  | hoarding | <0.001 [<0.001] | -0.1 \| 0.1 | 0.633 | <0.001 [<0.001] | -0.1 \| 0.1 | 0.709 |
| **Dorsal cingulum** | | | | | | | |
| AD | aggressive | 0.100 [0.100] | -0.2 \| 0.4 | 0.579 | 0.100 [0.100] | -0.2 \| 0.4 | 0.384 |
|  | sexual | <0.001 [0.200] | -0.3 \| 0.3 | 0.935 | <0.001 [0.200] | -0.3 \| 0.3 | 0.945 |
|  | symmetry | 0.100 [0.100] | -0.2 \| 0.3 | 0.630 | 0.100 [0.100] | -0.2 \| 0.3 | 0.591 |
|  | contamination | -0.200 [0.100] | -0.5 \| 0.1 | 0.141 | -0.200 [0.100] | -0.4 \| 0.1 | 0.269 |
|  | hoarding | 0.100 [0.200] | -0.4 \| 0.6 | 0.650 | 0.100 [0.300] | -0.4 \| 0.6 | 0.606 |
| FA | aggressive | <0.001 [0.100] | -0.2 \| 0.2 | 0.837 | <0.001 [0.100] | -0.2 \| 0.3 | 0.686 |
|  | sexual | 0.100 [0.100] | -0.1 \| 0.3 | 0.394 | 0.100 [0.100] | -0.1 \| 0.3 | 0.400 |
|  | symmetry | <0.001 [0.100] | -0.2 \| 0.3 | 0.654 | 0.100 [0.100] | -0.1 \| 0.3 | 0.531 |
|  | contamination | -0.200 [0.100] | -0.4 \| <0.001 | 0.051 | -0.100 [0.100] | -0.3 \| 0.1 | 0.194 |
|  | hoarding | 0.100 [0.200] | -0.3 \| 0.5 | 0.648 | 0.200 [0.200] | -0.2 \| 0.6 | 0.351 |
| MD | aggressive | <0.001 [0.100] | -0.1 \| 0.1 | 0.784 | <0.001 [0.100] | -0.1 \| 0.1 | 0.986 |
|  | sexual | -0.100 [0.100] | -0.2 \| 0.1 | 0.435 | -0.100 [0.100] | -0.2 \| 0.1 | 0.478 |
|  | symmetry | <0.001 [0.100] | -0.1 \| 0.1 | 0.893 | <0.001 [0.100] | -0.1 \| 0.1 | 0.967 |
|  | contamination | 0.100 [0.100] | <0.001 \| 0.2 | 0.236 | 0.100 [0.100] | -0.1 \| 0.2 | 0.387 |
|  | hoarding | 0.100 [0.100] | -0.2 \| 0.3 | 0.542 | <0.001 [0.100] | -0.2 \| 0.3 | 0.894 |
| RD | aggressive | <0.001 [0.100] | -0.2 \| 0.2 | 0.825 | <0.001 [0.100] | -0.2 \| 0.2 | 0.775 |
|  | sexual | -0.100 [0.100] | -0.3 \| 0.1 | 0.342 | -0.100 [0.100] | -0.3 \| 0.1 | 0.360 |
|  | symmetry | <0.001 [0.100] | -0.2 \| 0.1 | 0.714 | <0.001 [0.100] | -0.2 \| 0.1 | 0.603 |
|  | contamination | 0.200 [0.100] | <0.001 \| 0.4 | 0.038 | 0.100 [0.100] | <0.001 \| 0.3 | 0.129 |
|  | hoarding | <0.001 [0.200] | -0.3 \| 0.3 | 0.971 | -0.100 [0.200] | -0.4 \| 0.3 | 0.647 |
| ND | aggressive | <0.001 [0.100] | -0.2 \| 0.2 | 0.926 | 0.100 [0.100] | -0.1 \| 0.2 | 0.506 |
|  | sexual | <0.001 [0.100] | -0.1 \| 0.2 | 0.668 | <0.001 [0.100] | -0.1 \| 0.2 | 0.594 |
|  | symmetry | <0.001 [0.100] | -0.1 \| 0.2 | 0.761 | <0.001 [0.100] | -0.1 \| 0.2 | 0.638 |
|  | contamination | -0.200 [0.100] | -0.3 \| <0.001 | 0.043 | -0.100 [0.100] | -0.3 \| <0.001 | 0.138 |
|  | hoarding | <0.001 [0.100] | -0.3 \| 0.3 | 0.845 | <0.001 [0.200] | -0.3 \| 0.3 | 0.841 |
| OD | aggressive | <0.001 [<0.001] | -0.1 \| 0.1 | 0.969 | <0.001 [<0.001] | -0.1 \| 0.1 | 0.706 |
|  | sexual | <0.001 [<0.001] | -0.1 \| 0.1 | 0.824 | <0.001 [<0.001] | -0.1 \| 0.1 | 0.849 |
|  | symmetry | <0.001 [<0.001] | -0.1 \| 0.1 | 0.513 | <0.001 [<0.001] | -0.1 \| 0.1 | 0.456 |
|  | contamination | 0.100 [<0.001] | <0.001 \| 0.2 | 0.055 | 0.100 [<0.001] | <0.001 \| 0.1 | 0.151 |
|  | hoarding | <0.001 [0.100] | -0.2 \| 0.1 | 0.525 | -0.100 [0.100] | -0.2 \| 0.1 | 0.357 |
| **Ventral cingulum** | | | | | | | |
| AD | aggressive | -0.400 [0.200] | -0.8 \| <0.001 | 0.046 | -0.400 [0.200] | -0.8 \| <0.001 | 0.072 |
|  | sexual | <0.001 [0.200] | -0.4 \| 0.5 | 0.877 | <0.001 [0.200] | -0.4 \| 0.4 | 0.961 |
|  | symmetry | <0.001 [0.200] | -0.3 \| 0.4 | 0.809 | 0.100 [0.200] | -0.3 \| 0.4 | 0.760 |
|  | contamination | -0.200 [0.200] | -0.5 \| 0.2 | 0.402 | -0.100 [0.200] | -0.5 \| 0.3 | 0.564 |
|  | hoarding | -0.500 [0.400] | -1.2 \| 0.2 | 0.185 | -0.300 [0.400] | -1 \| 0.4 | 0.442 |
| FA | aggressive | -0.300 [0.100] | -0.5 \| -0.1 | 0.005 | -0.300 [0.100] | -0.5 \| <0.001 | 0.018 |
|  | sexual | <0.001 [0.100] | -0.2 \| 0.3 | 0.767 | 0.100 [0.100] | -0.2 \| 0.3 | 0.580 |
|  | symmetry | -0.100 [0.100] | -0.3 \| 0.1 | 0.242 | -0.100 [0.100] | -0.3 \| 0.1 | 0.309 |
|  | contamination | -0.200 [0.100] | -0.4 \| <0.001 | 0.064 | -0.200 [0.100] | -0.4 \| <0.001 | 0.131 |
|  | hoarding | <0.001 [0.200] | -0.4 \| 0.4 | 0.956 | -0.100 [0.200] | -0.5 \| 0.3 | 0.593 |
| MD | aggressive | -0.100 [0.100] | -0.3 \| 0.1 | 0.427 | -0.100 [0.100] | -0.3 \| 0.1 | 0.346 |
|  | sexual | <0.001 [0.100] | -0.2 \| 0.2 | 0.983 | <0.001 [0.100] | -0.3 \| 0.2 | 0.821 |
|  | symmetry | 0.100 [0.100] | -0.1 \| 0.3 | 0.293 | 0.100 [0.100] | -0.1 \| 0.3 | 0.320 |
|  | contamination | <0.001 [0.100] | -0.2 \| 0.2 | 0.739 | <0.001 [0.100] | -0.2 \| 0.2 | 0.772 |
|  | hoarding | -0.200 [0.200] | -0.6 \| 0.2 | 0.230 | -0.100 [0.200] | -0.5 \| 0.3 | 0.569 |
| RD | aggressive | 0.100 [0.100] | -0.1 \| 0.4 | 0.308 | 0.100 [0.100] | -0.2 \| 0.3 | 0.513 |
|  | sexual | -0.100 [0.100] | -0.3 \| 0.2 | 0.554 | -0.100 [0.100] | -0.4 \| 0.1 | 0.377 |
|  | symmetry | 0.200 [0.100] | -0.1 \| 0.4 | 0.190 | 0.100 [0.100] | -0.1 \| 0.4 | 0.232 |
|  | contamination | 0.200 [0.100] | -0.1 \| 0.4 | 0.196 | 0.100 [0.100] | -0.1 \| 0.4 | 0.282 |
|  | hoarding | -0.100 [0.200] | -0.5 \| 0.3 | 0.572 | <0.001 [0.200] | -0.4 \| 0.5 | 0.903 |
| ND | aggressive | <0.001 [0.100] | -0.2 \| 0.1 | 0.721 | <0.001 [0.100] | -0.2 \| 0.2 | 0.948 |
|  | sexual | 0.100 [0.100] | -0.1 \| 0.3 | 0.232 | 0.200 [0.100] | <0.001 \| 0.3 | 0.080 |
|  | symmetry | -0.100 [0.100] | -0.3 \| <0.001 | 0.145 | -0.100 [0.100] | -0.3 \| <0.001 | 0.166 |
|  | contamination | -0.100 [0.100] | -0.3 \| 0.1 | 0.350 | -0.100 [0.100] | -0.2 \| 0.1 | 0.420 |
|  | hoarding | 0.200 [0.200] | -0.1 \| 0.5 | 0.204 | <0.001 [0.200] | -0.3 \| 0.3 | 0.912 |
| OD | aggressive | 0.200 [0.100] | 0.1 \| 0.3 | 0.004 | 0.100 [0.100] | <0.001 \| 0.3 | 0.009 |
|  | sexual | <0.001 [0.100] | -0.1 \| 0.1 | 0.858 | <0.001 [0.100] | -0.1 \| 0.1 | 0.812 |
|  | symmetry | <0.001 [0.100] | -0.1 \| 0.1 | 0.784 | <0.001 [0.100] | -0.1 \| 0.1 | 0.712 |
|  | contamination | 0.100 [0.100] | <0.001 \| 0.2 | 0.178 | 0.100 [0.100] | <0.001 \| 0.2 | 0.289 |
|  | hoarding | <0.001 [0.100] | -0.2 \| 0.2 | 0.899 | <0.001 [0.100] | -0.2 \| 0.2 | 0.781 |
| **Postior thal Rad** | | | | | | | |
| AD | aggressive | -0.100 [0.100] | -0.3 \| 0.1 | 0.375 | -0.100 [0.100] | -0.3 \| 0.1 | 0.460 |
|  | sexual | -0.100 [0.100] | -0.4 \| 0.1 | 0.421 | -0.100 [0.100] | -0.4 \| 0.1 | 0.386 |
|  | symmetry | -0.200 [0.100] | -0.4 \| 0.1 | 0.137 | -0.200 [0.100] | -0.4 \| 0.1 | 0.140 |
|  | contamination | -0.100 [0.100] | -0.3 \| 0.1 | 0.244 | -0.100 [0.100] | -0.3 \| 0.1 | 0.344 |
|  | hoarding | 0.200 [0.200] | -0.2 \| 0.6 | 0.422 | 0.200 [0.200] | -0.2 \| 0.6 | 0.308 |
| FA | aggressive | 0.100 [0.100] | -0.1 \| 0.2 | 0.289 | 0.100 [0.100] | -0.1 \| 0.2 | 0.260 |
|  | sexual | <0.001 [0.100] | -0.1 \| 0.2 | 0.651 | <0.001 [0.100] | -0.2 \| 0.2 | 0.828 |
|  | symmetry | 0.100 [0.100] | -0.1 \| 0.2 | 0.264 | 0.100 [0.100] | -0.1 \| 0.2 | 0.277 |
|  | contamination | -0.200 [0.100] | -0.3 \| <0.001 | 0.032 | -0.100 [0.100] | -0.3 \| <0.001 | 0.055 |
|  | hoarding | -0.200 [0.100] | -0.5 \| 0.1 | 0.140 | -0.100 [0.100] | -0.4 \| 0.2 | 0.491 |
| MD | aggressive | -0.100 [0.100] | -0.2 \| <0.001 | 0.193 | -0.100 [0.100] | -0.2 \| 0.1 | 0.222 |
|  | sexual | -0.100 [0.100] | -0.2 \| 0.1 | 0.467 | <0.001 [0.100] | -0.2 \| 0.1 | 0.573 |
|  | symmetry | -0.100 [0.100] | -0.3 \| <0.001 | 0.064 | -0.100 [0.100] | -0.3 \| <0.001 | 0.071 |
|  | contamination | 0.100 [0.100] | -0.1 \| 0.2 | 0.295 | 0.100 [0.100] | -0.1 \| 0.2 | 0.317 |
|  | hoarding | 0.200 [0.100] | -0.1 \| 0.5 | 0.117 | 0.100 [0.100] | -0.1 \| 0.4 | 0.297 |
| RD | aggressive | -0.100 [0.100] | -0.3 \| 0.1 | 0.221 | -0.100 [0.100] | -0.3 \| 0.1 | 0.213 |
|  | sexual | -0.100 [0.100] | -0.3 \| 0.1 | 0.483 | <0.001 [0.100] | -0.2 \| 0.1 | 0.623 |
|  | symmetry | -0.100 [0.100] | -0.3 \| <0.001 | 0.140 | -0.100 [0.100] | -0.3 \| <0.001 | 0.150 |
|  | contamination | 0.200 [0.100] | <0.001 \| 0.3 | 0.062 | 0.100 [0.100] | <0.001 \| 0.3 | 0.091 |
|  | hoarding | 0.300 [0.200] | -0.1 \| 0.6 | 0.117 | 0.100 [0.200] | -0.2 \| 0.5 | 0.384 |
| ND | aggressive | 0.100 [0.100] | -0.1 \| 0.2 | 0.533 | <0.001 [0.100] | -0.1 \| 0.2 | 0.625 |
|  | sexual | <0.001 [0.100] | -0.2 \| 0.2 | 0.788 | <0.001 [0.100] | -0.2 \| 0.2 | 0.932 |
|  | symmetry | 0.100 [0.100] | -0.1 \| 0.3 | 0.285 | 0.100 [0.100] | -0.1 \| 0.3 | 0.320 |
|  | contamination | -0.100 [0.100] | -0.3 \| 0.1 | 0.396 | -0.100 [0.100] | -0.3 \| 0.1 | 0.391 |
|  | hoarding | -0.200 [0.200] | -0.5 \| 0.2 | 0.322 | -0.100 [0.200] | -0.4 \| 0.3 | 0.634 |
| OD | aggressive | <0.001 [<0.001] | -0.1 \| 0.1 | 0.975 | <0.001 [<0.001] | -0.1 \| 0.1 | 0.936 |
|  | sexual | <0.001 [<0.001] | <0.001 \| 0.1 | 0.487 | <0.001 [<0.001] | <0.001 \| 0.1 | 0.379 |
|  | symmetry | <0.001 [<0.001] | <0.001 \| 0.1 | 0.763 | <0.001 [<0.001] | <0.001 \| 0.1 | 0.737 |
|  | contamination | 0.100 [<0.001] | <0.001 \| 0.1 | 0.062 | <0.001 [<0.001] | <0.001 \| 0.1 | 0.107 |
|  | hoarding | <0.001 [0.100] | -0.1 \| 0.1 | 0.954 | <0.001 [0.100] | -0.1 \| 0.1 | 0.531 |
| **Sag Stratum** | | | | | | | |
| AD | aggressive | -0.100 [0.100] | -0.4 \| 0.1 | 0.198 | -0.100 [0.100] | -0.3 \| 0.1 | 0.238 |
|  | sexual | -0.100 [0.100] | -0.3 \| 0.2 | 0.584 | -0.100 [0.100] | -0.3 \| 0.2 | 0.514 |
|  | symmetry | -0.100 [0.100] | -0.3 \| 0.1 | 0.342 | -0.100 [0.100] | -0.3 \| 0.1 | 0.357 |
|  | contamination | -0.200 [0.100] | -0.4 \| <0.001 | 0.118 | -0.100 [0.100] | -0.4 \| 0.1 | 0.168 |
|  | hoarding | <0.001 [0.200] | -0.4 \| 0.4 | 0.907 | 0.100 [0.200] | -0.3 \| 0.5 | 0.587 |
| FA | aggressive | 0.100 [0.100] | -0.1 \| 0.3 | 0.177 | 0.100 [0.100] | -0.1 \| 0.3 | 0.260 |
|  | sexual | <0.001 [0.100] | -0.2 \| 0.2 | 0.948 | <0.001 [0.100] | -0.2 \| 0.2 | 0.962 |
|  | symmetry | 0.100 [0.100] | -0.1 \| 0.3 | 0.176 | 0.100 [0.100] | -0.1 \| 0.3 | 0.222 |
|  | contamination | <0.001 [0.100] | -0.1 \| 0.2 | 0.709 | <0.001 [0.100] | -0.1 \| 0.2 | 0.749 |
|  | hoarding | -0.200 [0.100] | -0.3 \| <0.001 | 0.037 | -0.200 [0.100] | -0.3 \| <0.001 | 0.048 |
| MD | aggressive | <0.001 [0.100] | -0.3 \| 0.3 | 0.928 | 0.100 [0.200] | -0.2 \| 0.4 | 0.666 |
|  | sexual | 0.100 [0.100] | <0.001 \| 0.3 | 0.071 | 0.100 [0.100] | <0.001 \| 0.3 | 0.111 |
|  | symmetry | -0.100 [0.100] | -0.2 \| 0.1 | 0.439 | <0.001 [0.100] | -0.2 \| 0.1 | 0.521 |
|  | contamination | -0.100 [0.100] | -0.3 \| <0.001 | 0.155 | -0.100 [0.100] | -0.3 \| <0.001 | 0.162 |
|  | hoarding | -0.100 [0.100] | -0.2 \| <0.001 | 0.182 | -0.100 [0.100] | -0.2 \| <0.001 | 0.207 |
| RD | aggressive | <0.001 [0.100] | -0.1 \| 0.2 | 0.586 | <0.001 [0.100] | -0.1 \| 0.2 | 0.518 |
|  | sexual | 0.100 [0.100] | -0.2 \| 0.3 | 0.581 | 0.100 [0.100] | -0.2 \| 0.3 | 0.538 |
|  | symmetry | <0.001 [0.100] | -0.1 \| 0.1 | 0.705 | <0.001 [0.100] | -0.1 \| 0.1 | 0.698 |
|  | contamination | <0.001 [0.100] | -0.2 \| 0.2 | 0.826 | <0.001 [0.100] | -0.2 \| 0.2 | 0.841 |
|  | hoarding | -0.200 [0.100] | -0.4 \| <0.001 | 0.100 | -0.200 [0.100] | -0.4 \| <0.001 | 0.112 |
| ND | aggressive | <0.001 [0.100] | -0.2 \| 0.1 | 0.614 | <0.001 [0.100] | -0.2 \| 0.1 | 0.650 |
|  | sexual | 0.100 [0.100] | -0.1 \| 0.3 | 0.180 | 0.100 [0.100] | -0.1 \| 0.3 | 0.196 |
|  | symmetry | <0.001 [0.200] | -0.3 \| 0.3 | 0.875 | <0.001 [0.200] | -0.3 \| 0.3 | 0.930 |
|  | contamination | -0.100 [0.100] | -0.3 \| <0.001 | 0.143 | -0.100 [0.100] | -0.3 \| <0.001 | 0.164 |
|  | hoarding | 0.100 [0.100] | -0.1 \| 0.3 | 0.322 | 0.100 [0.100] | -0.1 \| 0.3 | 0.378 |
| OD | aggressive | <0.001 [0.100] | -0.2 \| 0.2 | 0.889 | <0.001 [0.100] | -0.2 \| 0.2 | 0.871 |
|  | sexual | 0.100 [0.100] | -0.1 \| 0.3 | 0.349 | 0.100 [0.100] | -0.1 \| 0.2 | 0.379 |
|  | symmetry | -0.100 [0.100] | -0.3 \| 0.1 | 0.254 | -0.100 [0.100] | -0.3 \| 0.1 | 0.219 |
|  | contamination | 0.100 [0.200] | -0.3 \| 0.4 | 0.723 | <0.001 [0.200] | -0.3 \| 0.4 | 0.883 |
|  | hoarding | <0.001 [0.100] | -0.2 \| 0.1 | 0.839 | <0.001 [0.100] | -0.2 \| 0.1 | 0.899 |
| **SLF** | | | | | | | |
| AD | aggressive | <0.001 [0.100] | -0.1 \| 0.2 | 0.702 | 0.100 [0.100] | -0.1 \| 0.2 | 0.478 |
|  | sexual | <0.001 [0.100] | -0.2 \| 0.2 | 0.817 | <0.001 [0.100] | -0.2 \| 0.2 | 0.771 |
|  | symmetry | -0.100 [0.100] | -0.3 \| <0.001 | 0.136 | -0.100 [0.100] | -0.3 \| <0.001 | 0.169 |
|  | contamination | <0.001 [0.100] | -0.1 \| 0.2 | 0.608 | 0.100 [0.100] | -0.1 \| 0.2 | 0.375 |
|  | hoarding | 0.100 [0.200] | -0.2 \| 0.4 | 0.522 | 0.200 [0.200] | -0.1 \| 0.5 | 0.274 |
| FA | aggressive | 0.100 [0.100] | -0.1 \| 0.2 | 0.442 | 0.100 [0.100] | -0.1 \| 0.2 | 0.290 |
|  | sexual | <0.001 [0.100] | -0.1 \| 0.2 | 0.909 | <0.001 [0.100] | -0.2 \| 0.1 | 0.904 |
|  | symmetry | <0.001 [0.100] | -0.1 \| 0.1 | 0.789 | <0.001 [0.100] | -0.1 \| 0.1 | 0.833 |
|  | contamination | -0.100 [0.100] | -0.2 \| 0.1 | 0.279 | <0.001 [0.100] | -0.2 \| 0.1 | 0.521 |
|  | hoarding | <0.001 [0.100] | -0.2 \| 0.3 | 0.894 | 0.100 [0.100] | -0.1 \| 0.4 | 0.275 |
| MD | aggressive | <0.001 [0.100] | -0.1 \| 0.1 | 0.917 | <0.001 [0.100] | -0.1 \| 0.1 | 0.571 |
|  | sexual | <0.001 [0.100] | -0.1 \| 0.1 | 0.918 | <0.001 [0.100] | -0.1 \| 0.1 | 0.919 |
|  | symmetry | <0.001 [0.100] | -0.1 \| 0.1 | 0.727 | <0.001 [0.100] | -0.1 \| 0.1 | 0.796 |
|  | contamination | -0.100 [0.100] | -0.2 \| <0.001 | 0.225 | -0.100 [0.100] | -0.2 \| <0.001 | 0.240 |
|  | hoarding | 0.100 [0.100] | <0.001 \| 0.2 | 0.188 | 0.100 [0.100] | <0.001 \| 0.2 | 0.219 |
| RD | aggressive | 0.100 [0.100] | -0.1 \| 0.3 | 0.582 | <0.001 [0.100] | -0.2 \| 0.2 | 0.776 |
|  | sexual | <0.001 [0.100] | -0.2 \| 0.1 | 0.704 | <0.001 [0.100] | -0.2 \| 0.1 | 0.581 |
|  | symmetry | <0.001 [0.100] | -0.2 \| 0.1 | 0.744 | <0.001 [0.100] | -0.2 \| 0.1 | 0.870 |
|  | contamination | <0.001 [0.100] | -0.1 \| 0.1 | 0.826 | <0.001 [0.100] | -0.1 \| 0.1 | 0.807 |
|  | hoarding | 0.100 [0.100] | <0.001 \| 0.2 | 0.214 | 0.100 [0.100] | -0.1 \| 0.2 | 0.349 |
| ND | aggressive | <0.001 [0.100] | -0.2 \| 0.3 | 0.712 | <0.001 [0.100] | -0.3 \| 0.2 | 0.787 |
|  | sexual | 0.100 [0.100] | -0.1 \| 0.2 | 0.418 | 0.100 [0.100] | -0.1 \| 0.2 | 0.348 |
|  | symmetry | <0.001 [0.100] | -0.2 \| 0.1 | 0.799 | <0.001 [0.100] | -0.2 \| 0.2 | 0.884 |
|  | contamination | <0.001 [0.100] | -0.1 \| 0.2 | 0.928 | <0.001 [0.100] | -0.1 \| 0.2 | 0.907 |
|  | hoarding | -0.100 [0.100] | -0.3 \| <0.001 | 0.157 | -0.100 [0.100] | -0.3 \| 0.1 | 0.194 |
| OD | aggressive | 0.200 [0.100] | -0.1 \| 0.4 | 0.282 | 0.100 [0.100] | -0.2 \| 0.4 | 0.478 |
|  | sexual | <0.001 [<0.001] | -0.1 \| <0.001 | 0.566 | <0.001 [<0.001] | -0.1 \| <0.001 | 0.347 |
|  | symmetry | <0.001 [<0.001] | -0.1 \| 0.1 | 0.786 | <0.001 [<0.001] | -0.1 \| 0.1 | 0.619 |
|  | contamination | <0.001 [<0.001] | <0.001 \| 0.1 | 0.310 | <0.001 [<0.001] | <0.001 \| 0.1 | 0.352 |
|  | hoarding | <0.001 [<0.001] | <0.001 \| 0.1 | 0.560 | <0.001 [<0.001] | -0.1 \| 0.1 | 0.937 |
| **uncF** | | | | | | | |
| AD | aggressive | <0.001 [0.100] | -0.2 \| 0.3 | 0.903 | <0.001 [0.100] | -0.3 \| 0.3 | 0.995 |
|  | sexual | -0.300 [0.100] | -0.6 \| <0.001 | 0.064 | -0.300 [0.100] | -0.6 \| <0.001 | 0.029 |
|  | symmetry | <0.001 [0.100] | -0.2 \| 0.3 | 0.797 | <0.001 [0.100] | -0.2 \| 0.3 | 0.827 |
|  | contamination | 0.100 [0.100] | -0.2 \| 0.3 | 0.512 | 0.100 [0.100] | -0.2 \| 0.3 | 0.474 |
|  | hoarding | 0.100 [0.200] | -0.4 \| 0.5 | 0.794 | 0.300 [0.200] | -0.2 \| 0.8 | 0.218 |
| FA | aggressive | <0.001 [0.100] | -0.2 \| 0.3 | 0.754 | 0.100 [0.100] | -0.2 \| 0.3 | 0.570 |
|  | sexual | -0.200 [0.100] | -0.4 \| 0.1 | 0.143 | -0.200 [0.100] | -0.4 \| 0.1 | 0.157 |
|  | symmetry | <0.001 [0.100] | -0.2 \| 0.2 | 0.823 | <0.001 [0.100] | -0.2 \| 0.3 | 0.732 |
|  | contamination | -0.100 [0.100] | -0.3 \| 0.1 | 0.511 | <0.001 [0.100] | -0.3 \| 0.2 | 0.674 |
|  | hoarding | -0.300 [0.200] | -0.7 \| 0.2 | 0.223 | -0.200 [0.200] | -0.7 \| 0.2 | 0.273 |
| MD | aggressive | -0.100 [0.100] | -0.2 \| 0.1 | 0.309 | -0.100 [0.100] | -0.3 \| 0.1 | 0.193 |
|  | sexual | <0.001 [0.100] | -0.1 \| 0.2 | 0.770 | <0.001 [0.100] | -0.2 \| 0.2 | 0.903 |
|  | symmetry | 0.100 [0.100] | -0.1 \| 0.2 | 0.500 | <0.001 [0.100] | -0.1 \| 0.2 | 0.569 |
|  | contamination | 0.100 [0.100] | -0.1 \| 0.2 | 0.287 | 0.100 [0.100] | -0.1 \| 0.2 | 0.392 |
|  | hoarding | 0.300 [0.100] | <0.001 \| 0.6 | 0.045 | 0.300 [0.200] | 0.1 \| 0.6 | 0.022 |
| RD | aggressive | -0.100 [0.100] | -0.3 \| 0.1 | 0.382 | -0.100 [0.100] | -0.4 \| 0.1 | 0.229 |
|  | sexual | 0.200 [0.100] | -0.1 \| 0.4 | 0.148 | 0.200 [0.100] | -0.1 \| 0.4 | 0.184 |
|  | symmetry | <0.001 [0.100] | -0.2 \| 0.2 | 0.945 | <0.001 [0.100] | -0.2 \| 0.2 | 0.957 |
|  | contamination | 0.100 [0.100] | -0.1 \| 0.3 | 0.337 | 0.100 [0.100] | -0.1 \| 0.3 | 0.498 |
|  | hoarding | 0.300 [0.200] | -0.1 \| 0.7 | 0.101 | 0.400 [0.200] | <0.001 \| 0.8 | 0.080 |
| ND | aggressive | 0.100 [0.100] | -0.1 \| 0.3 | 0.239 | 0.100 [0.100] | <0.001 \| 0.3 | 0.112 |
|  | sexual | -0.100 [0.100] | -0.3 \| 0.1 | 0.167 | -0.100 [0.100] | -0.3 \| 0.1 | 0.301 |
|  | symmetry | <0.001 [0.100] | -0.1 \| 0.2 | 0.732 | <0.001 [0.100] | -0.1 \| 0.2 | 0.607 |
|  | contamination | -0.100 [0.100] | -0.3 \| <0.001 | 0.089 | -0.100 [0.100] | -0.3 \| <0.001 | 0.114 |
|  | hoarding | -0.100 [0.100] | -0.4 \| 0.2 | 0.443 | -0.300 [0.100] | -0.5 \| <0.001 | 0.073 |
| OD | aggressive | <0.001 [<0.001] | -0.1 \| 0.1 | 0.874 | <0.001 [<0.001] | -0.1 \| 0.1 | 0.842 |
|  | sexual | 0.100 [<0.001] | <0.001 \| 0.1 | 0.228 | 0.100 [<0.001] | <0.001 \| 0.2 | 0.139 |
|  | symmetry | <0.001 [<0.001] | -0.1 \| 0.1 | 0.644 | <0.001 [<0.001] | -0.1 \| 0.1 | 0.661 |
|  | contamination | <0.001 [<0.001] | -0.1 \| 0.1 | 0.502 | <0.001 [<0.001] | -0.1 \| <0.001 | 0.429 |
|  | hoarding | <0.001 [0.100] | -0.1 \| 0.2 | 0.683 | <0.001 [0.100] | -0.2 \| 0.1 | 0.639 |
| False Discovery rate (FDR) significant differences are marked in bold. P<0.05, uncorrected significant results are underlined. *Corrected for age, sex and education in years; Abbreviations: FA = fractional anisotropy, MD = mean diffusivity, RD = radial diffusivity, AD = axial diffusivity, ND = neurite density, OD = orientation dispersion, SLF = superior longitudinal fascicle, CC genu = genu of the corpus callosum, CC body = body of the corpus callosum, CC splenium = splenium of the corpus callosum, uncF = uncinate fascicle, Sag Stratum = sagittal stratum, Posterior thal rad = posterior thalamic radiation | | | | | | | |

**Fixel analyses**

| **Supplementary Table 12 – Case control mixed model analyses of fixel based measures - ComBat** | | | | | | | | |
| --- | --- | --- | --- | --- | --- | --- | --- | --- |
|  | **HC** | **OCD** | **Group difference (crude model)#** | | | **Group difference (adjusted model)*** | | |
|  | M ± SD  (* 10^-1^) | M ± SD  (* 10^-1^) | B [SE] | 95% CI | P (unc) | B [SE] | 95% CI | P (unc) |
| **CC genu** | | | | | | | | |
| FDC | 6.416 ± 0.99 | 6.543 ± 0.944 | 0.080 [0.080] | -0.07 \| 0.23 | 0.442 (0.295) | 0.070 [0.080] | -0.08 \| 0.21 | 0.559 (0.373) |
| FD | 6.336 ± 0.85 | 6.445 ± 0.813 | 0.110 [0.070] | -0.04 \| 0.25 | 0.308 (0.140) | 0.100 [0.070] | -0.04 \| 0.25 | 0.301 (0.167) |
| FC | 0.098 ± 0.74 | 0.124 ± 0.685 | -0.040 [0.030] | -0.1 \| 0.02 | 0.565 (0.157) | -0.040 [0.030] | -0.1 \| 0.01 | 0.486 (0.108) |
| **CC body** | | | | | | | | |
| FDC | 5.889 ± 1.241 | 6.047 ± 1.257 | 0.120 [0.110] | -0.09 \| 0.33 | 0.442 (0.250) | 0.110 [0.110] | -0.1 \| 0.32 | 0.559 (0.312) |
| FD | 5.83 ± 1.205 | 5.958 ± 1.2 | 0.130 [0.110] | -0.08 \| 0.34 | 0.344 (0.229) | 0.110 [0.110] | -0.1 \| 0.32 | 0.429 (0.286) |
| FC | 0.098 ± 0.742 | 0.124 ± 0.685 | -0.040 [0.030] | -0.1 \| 0.02 | 0.565 (0.159) | -0.050 [0.030] | -0.1 \| 0.01 | 0.486 (0.107) |
| **CC splenium** | | | | | | | | |
| FDC | 7.919 ± 1.107 | 8.085 ± 1.106 | 0.120 [0.090] | -0.06 \| 0.29 | 0.414 (0.184) | 0.120 [0.090] | -0.06 \| 0.29 | 0.454 (0.202) |
| FD | 7.837 ± 0.956 | 7.977 ± 1.005 | 0.140 [0.090] | -0.03 \| 0.31 | 0.308 (0.107) | 0.140 [0.090] | -0.03 \| 0.31 | 0.301 (0.110) |
| FC | 0.081 ± 0.726 | 0.118 ± 0.668 | -0.030 [0.030] | -0.08 \| 0.02 | 0.565 (0.251) | -0.030 [0.030] | -0.08 \| 0.02 | 0.558 (0.186) |
| **Dorsal cingulum bundle** | | | | | | | | |
| FDC | 4.127 ± 0.609 | 4.225 ± 0.598 | 0.070 [0.050] | -0.02 \| 0.17 | 0.414 (0.132) | 0.080 [0.050] | -0.01 \| 0.18 | 0.400 (0.089) |
| FD | 4.088 ± 0.521 | 4.162 ± 0.535 | 0.070 [0.050] | -0.02 \| 0.17 | 0.308 (0.114) | 0.090 [0.050] | 0 \| 0.18 | 0.265 (0.059) |
| FC | 0.068 ± 0.718 | 0.131 ± 0.654 | -0.000 [0.020] | -0.05 \| 0.04 | 0.892 (0.892) | -0.000 [0.020] | -0.05 \| 0.04 | 0.839 (0.827) |
| **Ventral cingulum bundle** | | | | | | | | |
| FDC | 3.917 ± 0.654 | 3.929 ± 0.74 | -0.010 [0.060] | -0.12 \| 0.11 | 0.891 (0.891) | -0.010 [0.060] | -0.13 \| 0.11 | 0.897 (0.868) |
| FD | 3.892 ± 0.599 | 3.873 ± 0.721 | -0.020 [0.060] | -0.13 \| 0.1 | 0.748 (0.748) | -0.010 [0.060] | -0.13 \| 0.1 | 0.807 (0.807) |
| FC | 0.024 ± 0.747 | 0.121 ± 0.669 | 0.030 [0.030] | -0.02 \| 0.08 | 0.565 (0.247) | 0.030 [0.030] | -0.02 \| 0.08 | 0.623 (0.277) |
| **Posterior thal rad** | | | | | | | | |
| FDC | 4.739 ± 0.532 | 4.753 ± 0.496 | -0.020 [0.040] | -0.09 \| 0.05 | 0.744 (0.579) | -0.020 [0.040] | -0.09 \| 0.05 | 0.721 (0.561) |
| FD | 4.696 ± 0.369 | 4.682 ± 0.389 | -0.010 [0.030] | -0.08 \| 0.05 | 0.748 (0.687) | -0.010 [0.030] | -0.08 \| 0.06 | 0.807 (0.736) |
| FC | 0.05 ± 0.718 | 0.128 ± 0.648 | 0.010 [0.020] | -0.03 \| 0.05 | 0.848 (0.579) | 0.010 [0.020] | -0.03 \| 0.05 | 0.839 (0.683) |
| **Sag Stratum** | | | | | | | | |
| FDC | 5.387 ± 0.709 | 5.527 ± 0.709 | 0.100 [0.050] | 0 \| 0.21 | 0.414 (0.056) | 0.100 [0.050] | 0 \| 0.21 | 0.400 (0.049) |
| FD | 5.347 ± 0.565 | 5.45 ± 0.586 | 0.100 [0.050] | 0 \| 0.2 | 0.308 (0.044) | 0.120 [0.050] | 0.02 \| 0.21 | 0.216 (0.024) |
| FC | 0.036 ± 0.742 | 0.117 ± 0.663 | 0.010 [0.030] | -0.04 \| 0.07 | 0.848 (0.580) | 0.010 [0.030] | -0.04 \| 0.06 | 0.839 (0.653) |
| **SLF** | | | | | | | | |
| FDC | 4.063 ± 0.439 | 4.102 ± 0.444 | 0.010 [0.030] | -0.05 \| 0.07 | 0.824 (0.732) | 0.000 [0.030] | -0.06 \| 0.07 | 0.897 (0.897) |
| FD | 4.01 ± 0.298 | 4.035 ± 0.346 | 0.020 [0.030] | -0.03 \| 0.08 | 0.492 (0.383) | 0.020 [0.030] | -0.04 \| 0.08 | 0.612 (0.476) |
| FC | 0.07 ± 0.71 | 0.129 ± 0.651 | -0.010 [0.020] | -0.05 \| 0.04 | 0.848 (0.754) | -0.010 [0.020] | -0.05 \| 0.03 | 0.839 (0.672) |
| **uncF** | | | | | | | | |
| FDC | 6.123 ± 1.138 | 6.296 ± 1.075 | 0.130 [0.090] | -0.05 \| 0.31 | 0.414 (0.150) | 0.140 [0.090] | -0.05 \| 0.32 | 0.438 (0.146) |
| FD | 6.065 ± 1.058 | 6.187 ± 0.952 | 0.120 [0.090] | -0.05 \| 0.3 | 0.308 (0.171) | 0.130 [0.090] | -0.05 \| 0.3 | 0.301 (0.166) |
| FC | 0.071 ± 0.718 | 0.145 ± 0.659 | 0.010 [0.020] | -0.04 \| 0.05 | 0.848 (0.738) | 0.000 [0.020] | -0.04 \| 0.05 | 0.839 (0.839) |
| Reported fixel measures are adjusted for site using Combat. False Discovery rate (FDR) significant differences are marked in bold. P<0.05, uncorrected significant results are underlined. *Corrected for age, sex and education in years; Abbreviations: FDC = fiber density and cross-section, FD = fiber density, FC = fiber cross-section. SLF = superior longitudinal fascicle, CC genu = genu of the corpus callosum, CC body = body of the corpus callosum, CC splenium = splenium of the corpus callosum, uncF = uncinate fascicle, Sag Stratum = sagittal stratum, Posterior thal rad = posterior thalamic radiation. | | | | | | | | |

| **Supplementary Table 13 – mixed model analyses of fixel measures and case-control differences – random effects for site** | | | | | | | | | | |
| --- | --- | --- | --- | --- | --- | --- | --- | --- | --- | --- |
|  | **Group difference (crude model)** | | | **Group difference (adjusted model)*** | | | | | | |
|  | B [SE]  (* 10^-1^) | 95% CI  (* 10^-1^) | P (unc) | B [SE]  (* 10^-1^) | | | 95% CI  (* 10^-1^) | | P (unc) | |
| **CC genu** | | | | | | | | | | |
| FDC | 0.090 [0.080] | -0.06 \| 0.24 | 0.368 (0.232) | 0.080 [0.080] | | | -0.07 \| 0.23 | | 0.462 (0.297) | |
| FD | 0.120 [0.070] | -0.03 \| 0.26 | 0.254 (0.119) | 0.110 [0.070] | | | -0.04 \| 0.26 | | 0.256 (0.142) | |
| FC | -0.040 [0.030] | -0.09 \| 0.02 | 0.704 (0.170) | -0.040 [0.030] | | | -0.1 \| 0.01 | | 0.657 (0.138) | |
| **CC body** | | | | | | | | | | |
| FDC | 0.130 [0.110] | -0.09 \| 0.34 | 0.368 (0.245) | 0.110 [0.110] | | -0.1 \| 0.32 | | 0.462 (0.308) | | |
| FD | 0.130 [0.110] | -0.08 \| 0.34 | 0.350 (0.233) | 0.110 [0.110] | | -0.1 \| 0.32 | | 0.434 (0.289) | | |
| FC | -0.040 [0.030] | -0.09 \| 0.02 | 0.704 (0.179) | -0.040 [0.030] | | -0.1 \| 0.01 | | 0.657 (0.146) | | |
| **CC splenium** | | | | | | | | | | |
| FDC | 0.130 [0.090] | -0.05 \| 0.31 | 0.324 (0.144) | 0.130 [0.090] | | -0.05 \| 0.31 | | | 0.360 (0.160) | |
| FD | 0.150 [0.090] | -0.02 \| 0.32 | 0.254 (0.093) | 0.150 [0.090] | | -0.03 \| 0.33 | | | 0.256 (0.094) | |
| FC | -0.030 [0.030] | -0.08 \| 0.02 | 0.704 (0.260) | -0.030 [0.030] | | -0.08 \| 0.02 | | | 0.678 (0.226) | |
| **Dorsal cingulum** | | | | | | | | | | |
| FDC | 0.080 [0.050] | -0.02 \| 0.17 | 0.324 (0.129) | | 0.090 [0.050] | | -0.01 \| 0.18 | | | 0.300 (0.088) |
| FD | 0.070 [0.050] | -0.02 \| 0.17 | 0.254 (0.116) | | 0.090 [0.050] | | <0.001 \| 0.18 | | | 0.256 (0.062) |
| FC | -0.010 [0.020] | -0.05 \| 0.04 | 0.815 (0.793) | | -0.010 [0.020] | | -0.05 \| 0.04 | | | 0.890 (0.756) |
| **Ventral cingulum** | | | | | | | | | | |
| FDC | <0.001 [0.060] | -0.12 \| 0.11 | 0.960 (0.960) | | -0.010 [0.060] | | -0.13 \| 0.11 | | | 0.888 (0.888) |
| FD | -0.010 [0.060] | -0.13 \| 0.1 | 0.807 (0.807) | | -0.010 [0.060] | | -0.13 \| 0.1 | | | 0.828 (0.828) |
| FC | 0.030 [0.030] | -0.03 \| 0.08 | 0.704 (0.313) | | 0.030 [0.030] | | -0.03 \| 0.08 | | | 0.749 (0.333) |
| **Posterior thal rad** | | | | | | | | | | |
| FDC | -0.020 [0.040] | -0.09 \| 0.05 | 0.693 (0.616) | | -0.020 [0.040] | | -0.09 \| 0.05 | | | 0.796 (0.619) |
| FD | -0.010 [0.030] | -0.08 \| 0.05 | 0.773 (0.687) | | -0.010 [0.030] | | -0.08 \| 0.06 | | | 0.828 (0.766) |
| FC | 0.010 [0.020] | -0.03 \| 0.05 | 0.815 (0.685) | | 0.010 [0.020] | | -0.04 \| 0.05 | | | 0.890 (0.791) |
| **Sag Stratum** | | | | | | | | | | |
| FDC | 0.110 [0.050] | 0.01 \| 0.22 | 0.306 (0.034) | 0.120 [0.050] | | 0.01 \| 0.22 | | | 0.270 (0.030) | |
| FD | 0.110 [0.050] | 0.01 \| 0.22 | 0.252 (0.028) | 0.130 [0.050] | | 0.02 \| 0.23 | | | 0.144 (0.016) | |
| FC | 0.010 [0.030] | -0.04 \| 0.06 | 0.815 (0.694) | 0.010 [0.030] | | -0.04 \| 0.06 | | | 0.890 (0.756) | |
| **SLF** | | | | | | | | | | |
| FDC | 0.020 [0.030] | -0.05 \| 0.08 | 0.693 (0.599) | 0.010 [0.030] | | | -0.05 \| 0.07 | | | 0.870 (0.773) |
| FD | 0.030 [0.030] | -0.03 \| 0.09 | 0.423 (0.329) | 0.020 [0.030] | | | -0.03 \| 0.08 | | | 0.552 (0.429) |
| FC | -0.010 [0.020] | -0.05 \| 0.03 | 0.815 (0.689) | -0.010 [0.020] | | | -0.05 \| 0.03 | | | 0.890 (0.641) |
| **uncF** | | | | | | | | | | |
| FDC | 0.150 [0.090] | -0.03 \| 0.33 | 0.324 (0.102) | 0.150 [0.090] | | | -0.03 \| 0.34 | | | 0.300 (0.100) |
| FD | 0.130 [0.090] | -0.04 \| 0.31 | 0.254 (0.141) | 0.140 [0.090] | | | -0.04 \| 0.31 | | | 0.256 (0.137) |
| FC | 0.010 [0.020] | -0.04 \| 0.05 | 0.815 (0.815) | <0.001 [0.020] | | | -0.04 \| 0.05 | | | 0.926 (0.926) |
| False Discovery rate (FDR) significant differences are marked in bold. P<0.05, uncorrected significant results are underlined. *Corrected for age, sex and education in years; Abbreviations: SLF = superior longitudinal fascicle, CC genu = genu of the corpus callosum, CC body = body of the corpus callosum, CC splenium = splenium of the corpus callosum. | | | | | | | | | | |

| **Supplementary Table 14 – OCD onset mixed model analyses of fixel based measures – ComBat corrected** | | | | | | | | |
| --- | --- | --- | --- | --- | --- | --- | --- | --- |
|  | **ADULT** | **CHILD** | **Group difference (crude model)#** | | | **Group difference (adjusted model)*** | | |
|  | M ± SD  (* 10^-1^) | M ± SD  (* 10^-1^) | B [SE] | 95% CI | P (unc) | B [SE] | 95% CI | P (unc) |
| **CC genu** | | | | | | | | |
| FDC | 6.579 ± 0.98 | 6.513 ± 0.92 | -0.080 [0.110] | -0.29 \| 0.13 | 0.815 (0.468) | -0.100 [0.110] | -0.31 \| 0.12 | 0.867 (0.377) |
| FD | 6.469 ± 0.848 | 6.423 ± 0.788 | -0.050 [0.100] | -0.25 \| 0.15 | 0.928 (0.650) | -0.090 [0.100] | -0.29 \| 0.11 | 0.876 (0.389) |
| FC | 0.144 ± 0.688 | 0.11 ± 0.686 | -0.050 [0.040] | -0.13 \| 0.02 | 0.492 (0.164) | -0.030 [0.040] | -0.1 \| 0.05 | 0.918 (0.497) |
| **CC body** | | | | | | | | |
| FDC | 5.991 ± 1.229 | 6.097 ± 1.283 | 0.100 [0.150] | -0.21 \| 0.4 | 0.815 (0.529) | 0.050 [0.160] | -0.26 \| 0.35 | 0.867 (0.771) |
| FD | 5.896 ± 1.169 | 6.011 ± 1.228 | 0.120 [0.150] | -0.18 \| 0.41 | 0.928 (0.445) | 0.060 [0.150] | -0.24 \| 0.36 | 0.876 (0.689) |
| FC | 0.147 ± 0.696 | 0.107 ± 0.681 | -0.060 [0.040] | -0.13 \| 0.02 | 0.492 (0.133) | -0.030 [0.040] | -0.11 \| 0.05 | 0.918 (0.455) |
| **CC splenium** | | | | | | | | |
| FDC | 8.121 ± 1.087 | 8.059 ± 1.127 | -0.070 [0.130] | -0.33 \| 0.18 | 0.815 (0.574) | -0.050 [0.130] | -0.32 \| 0.21 | 0.867 (0.702) |
| FD | 8.0 ± 0.994 | 7.959 ± 1.02 | -0.040 [0.130] | -0.29 \| 0.21 | 0.928 (0.749) | -0.020 [0.130] | -0.27 \| 0.23 | 0.876 (0.876) |
| FC | 0.137 ± 0.674 | 0.105 ± 0.667 | -0.050 [0.030] | -0.12 \| 0.02 | 0.492 (0.141) | -0.030 [0.030] | -0.1 \| 0.04 | 0.918 (0.394) |
| **Dorsal cingulum bundle** | | | | | | | | |
| FDC | 4.244 ± 0.538 | 4.215 ± 0.643 | -0.030 [0.070] | -0.18 \| 0.11 | 0.815 (0.634) | 0.010 [0.070] | -0.14 \| 0.15 | 0.919 (0.919) |
| FD | 4.182 ± 0.485 | 4.149 ± 0.571 | -0.030 [0.070] | -0.16 \| 0.1 | 0.928 (0.623) | 0.010 [0.070] | -0.12 \| 0.15 | 0.876 (0.836) |
| FC | 0.122 ± 0.616 | 0.141 ± 0.687 | 0.000 [0.030] | -0.06 \| 0.06 | 0.993 (0.976) | 0.010 [0.030] | -0.05 \| 0.07 | 0.918 (0.741) |
| **Ventral cingulum bundle** | | | | | | | | |
| FDC | 3.891 ± 0.723 | 3.958 ± 0.755 | 0.060 [0.090] | -0.12 \| 0.24 | 0.815 (0.492) | 0.120 [0.090] | -0.06 \| 0.3 | 0.867 (0.189) |
| FD | 3.842 ± 0.722 | 3.894 ± 0.723 | 0.050 [0.090] | -0.13 \| 0.23 | 0.928 (0.567) | 0.110 [0.090] | -0.07 \| 0.29 | 0.876 (0.223) |
| FC | 0.114 ± 0.635 | 0.132 ± 0.696 | -0.000 [0.040] | -0.07 \| 0.07 | 0.993 (0.993) | -0.010 [0.040] | -0.08 \| 0.06 | 0.918 (0.840) |
| **Posterior thal rad** | | | | | | | | |
| FDC | 4.734 ± 0.487 | 4.769 ± 0.506 | 0.030 [0.050] | -0.07 \| 0.13 | 0.815 (0.599) | 0.030 [0.050] | -0.07 \| 0.13 | 0.867 (0.574) |
| FD | 4.669 ± 0.405 | 4.693 ± 0.377 | 0.020 [0.050] | -0.07 \| 0.12 | 0.928 (0.623) | 0.020 [0.050] | -0.08 \| 0.12 | 0.876 (0.658) |
| FC | 0.119 ± 0.612 | 0.138 ± 0.678 | 0.000 [0.030] | -0.05 \| 0.06 | 0.993 (0.960) | 0.010 [0.030] | -0.05 \| 0.06 | 0.918 (0.823) |
| **Sag Stratum** | | | | | | | | |
| FDC | 5.515 ± 0.656 | 5.536 ± 0.753 | 0.010 [0.080] | -0.14 \| 0.16 | 0.957 (0.887) | 0.070 [0.080] | -0.08 \| 0.22 | 0.867 (0.330) |
| FD | 5.444 ± 0.55 | 5.451 ± 0.615 | 0.010 [0.070] | -0.14 \| 0.15 | 0.928 (0.928) | 0.050 [0.070] | -0.1 \| 0.19 | 0.876 (0.518) |
| FC | 0.109 ± 0.625 | 0.128 ± 0.693 | 0.000 [0.030] | -0.07 \| 0.07 | 0.993 (0.990) | -0.000 [0.030] | -0.07 \| 0.06 | 0.918 (0.918) |
| **SLF** | | | | | | | | |
| FDC | 4.084 ± 0.425 | 4.118 ± 0.461 | 0.030 [0.050] | -0.07 \| 0.12 | 0.815 (0.573) | 0.040 [0.050] | -0.06 \| 0.13 | 0.867 (0.412) |
| FD | 4.013 ± 0.33 | 4.052 ± 0.36 | 0.040 [0.040] | -0.05 \| 0.12 | 0.928 (0.367) | 0.050 [0.040] | -0.04 \| 0.13 | 0.876 (0.292) |
| FC | 0.131 ± 0.623 | 0.13 ± 0.677 | -0.020 [0.030] | -0.08 \| 0.04 | 0.993 (0.512) | -0.010 [0.030] | -0.06 \| 0.05 | 0.918 (0.840) |
| **uncF** | | | | | | | | |
| FDC | 6.299 ± 1.069 | 6.304 ± 1.079 | -0.010 [0.130] | -0.25 \| 0.24 | 0.957 (0.957) | 0.050 [0.130] | -0.21 \| 0.3 | 0.867 (0.718) |
| FD | 6.2 ± 0.933 | 6.187 ± 0.966 | -0.010 [0.120] | -0.25 \| 0.22 | 0.928 (0.913) | 0.020 [0.120] | -0.22 \| 0.26 | 0.876 (0.875) |
| FC | 0.127 ± 0.625 | 0.161 ± 0.689 | 0.020 [0.030] | -0.04 \| 0.08 | 0.993 (0.582) | 0.030 [0.030] | -0.03 \| 0.09 | 0.918 (0.347) |
| Reported fixel measures are adjusted for site using Combat. False Discovery rate (FDR) significant differences are marked in bold. P<0.05, uncorrected significant results are underlined. *Corrected for intracranial volume, age, sex and education in years; Abbreviations: FDC = fiber density and cross-section, FD = fiber density, FC = fiber cross-section. , SLF = superior longitudinal fascicle, CC genu = genu of the corpus callosum, CC body = body of the corpus callosum, CC splenium = splenium of the corpus callosum, uncF = uncinate fascicle, Sag Stratum = sagittal stratum, Posterior thal rad = posterior thalamic radiation. | | | | | | | | |

| **Supplementary Table 15 – Age of OCD onset mixed model analyses of fixel measures– random effects for site** | | | | | | |
| --- | --- | --- | --- | --- | --- | --- |
|  | **Group difference (crude model)** | | | **Group difference (adjusted model)*** | | |
|  | B [SE]  (* 10^-1^) | 95% CI  (* 10^-1^) | P (unc) | B [SE]  (* 10^-1^) | 95% CI  (* 10^-1^) | P (unc) |
| **CC genu** | | | | | | |
| FDC | -0.070 [0.110] | -0.29 \| 0.14 | 0.851 (0.508) | -0.080 [0.110] | -0.3 \| 0.13 | 0.850 (0.447) |
| FD | -0.040 [0.100] | -0.24 \| 0.17 | 0.928 (0.709) | -0.090 [0.110] | -0.29 \| 0.12 | 0.957 (0.422) |
| FC | -0.060 [0.040] | -0.13 \| 0.02 | 0.459 (0.153) | -0.020 [0.040] | -0.1 \| 0.06 | 0.989 (0.586) |
| **CC body** | | | | | | |
| FDC | 0.100 [0.160] | -0.21 \| 0.41 | 0.851 (0.529) | 0.050 [0.160] | -0.27 \| 0.37 | 0.887 (0.754) |
| FD | 0.120 [0.150] | -0.18 \| 0.43 | 0.928 (0.427) | 0.070 [0.160] | -0.24 \| 0.38 | 0.957 (0.673) |
| FC | -0.060 [0.040] | -0.14 \| 0.02 | 0.459 (0.132) | -0.020 [0.040] | -0.1 \| 0.06 | 0.989 (0.564) |
| **CC splenium** | | | | | | |
| FDC | -0.060 [0.140] | -0.33 \| 0.2 | 0.851 (0.635) | -0.040 [0.140] | -0.32 \| 0.24 | 0.887 (0.788) |
| FD | -0.030 [0.130] | -0.29 \| 0.23 | 0.928 (0.823) | -0.010 [0.140] | -0.27 \| 0.26 | 0.957 (0.957) |
| FC | -0.050 [0.040] | -0.12 \| 0.02 | 0.459 (0.139) | -0.030 [0.040] | -0.1 \| 0.04 | 0.989 (0.461) |
| **Dorsal cingulum** | | | | | | |
| FDC | -0.050 [0.080] | -0.2 \| 0.1 | 0.851 (0.544) | <0.001 [0.080] | -0.15 \| 0.16 | 0.957 (0.957) |
| FD | -0.050 [0.070] | -0.19 \| 0.09 | 0.928 (0.475) | 0.010 [0.070] | -0.13 \| 0.15 | 0.957 (0.903) |
| FC | <0.001 [0.030] | -0.06 \| 0.06 | 0.959 (0.959) | 0.010 [0.030] | -0.05 \| 0.07 | 0.989 (0.746) |
| **Ventral cingulum** | | | | | | |
| FDC | 0.050 [0.090] | -0.14 \| 0.23 | 0.851 (0.604) | 0.120 [0.100] | -0.07 \| 0.31 | 0.850 (0.206) |
| FD | 0.030 [0.090] | -0.15 \| 0.22 | 0.928 (0.724) | 0.110 [0.100] | -0.08 \| 0.3 | 0.957 (0.256) |
| FC | 0.010 [0.040] | -0.06 \| 0.08 | 0.959 (0.803) | <0.001 [0.040] | -0.07 \| 0.07 | 0.989 (0.989) |
| **Posterior thal rad** | | | | | | |
| FDC | 0.030 [0.050] | -0.07 \| 0.14 | 0.851 (0.551) | 0.040 [0.050] | -0.07 \| 0.15 | 0.850 (0.472) |
| FD | 0.020 [0.050] | -0.08 \| 0.12 | 0.928 (0.667) | 0.020 [0.050] | -0.08 \| 0.13 | 0.957 (0.640) |
| FC | 0.010 [0.030] | -0.05 \| 0.06 | 0.959 (0.855) | 0.010 [0.030] | -0.05 \| 0.07 | 0.989 (0.668) |
| **Sag Stratum** | | | | | | |
| FDC | <0.001 [0.080] | -0.15 \| 0.16 | 0.990 (0.990) | 0.090 [0.080] | -0.07 \| 0.24 | 0.850 (0.266) |
| FD | -0.010 [0.080] | -0.16 \| 0.14 | 0.928 (0.928) | 0.050 [0.080] | -0.1 \| 0.2 | 0.957 (0.498) |
| FC | 0.010 [0.030] | -0.06 \| 0.07 | 0.959 (0.847) | <0.001 [0.040] | -0.07 \| 0.07 | 0.989 (0.958) |
| **SLF** | | | | | | |
| FDC | 0.020 [0.050] | -0.07 \| 0.12 | 0.851 (0.662) | 0.040 [0.050] | -0.06 \| 0.14 | 0.850 (0.471) |
| FD | 0.030 [0.050] | -0.05 \| 0.12 | 0.928 (0.451) | 0.040 [0.050] | -0.05 \| 0.13 | 0.957 (0.367) |
| FC | -0.020 [0.030] | -0.08 \| 0.04 | 0.959 (0.449) | -0.010 [0.030] | -0.07 \| 0.05 | 0.989 (0.833) |
| **uncF** | | | | | | |
| FDC | -0.010 [0.130] | -0.26 \| 0.24 | 0.990 (0.942) | 0.060 [0.130] | -0.2 \| 0.32 | 0.887 (0.649) |
| FD | -0.020 [0.120] | -0.26 \| 0.22 | 0.928 (0.846) | 0.020 [0.130] | -0.23 \| 0.27 | 0.957 (0.883) |
| FC | 0.020 [0.030] | -0.04 \| 0.08 | 0.959 (0.540) | 0.040 [0.030] | -0.03 \| 0.1 | 0.989 (0.246) |
| False Discovery rate (FDR) significant differences are marked in bold. P<0.05, uncorrected significant results are underlined. *Corrected for age, sex and education in years; Abbreviations: SLF = superior longitudinal fascicle, CC genu = genu of the corpus callosum, CC body = body of the corpus callosum, CC splenium = splenium of the corpus callosum. | | | | | | |

| **Supplementary Table 16 – SSRI/SNRI history mixed model analyses of fixel based measures – ComBat corrected** | | | | | | | | |
| --- | --- | --- | --- | --- | --- | --- | --- | --- |
|  | **NAIVE** | **NOT NAIVE** | **Group difference (crude model)#** | | | **Group difference (adjusted model)*** | | |
|  | M ± SD  (* 10^-1^) | M ± SD  (* 10^-1^) | B [SE] | 95% CI | P (unc) | B [SE] | 95% CI | P (unc) |
| **CC genu** | | | | | | | | |
| FDC | 6.571 ± 0.935 | 6.521 ± 0.952 | -0.040 [0.110] | -0.25 \| 0.17 | 0.828 (0.719) | -0.060 [0.110] | -0.27 \| 0.15 | 0.761 (0.585) |
| FD | 6.458 ± 0.772 | 6.435 ± 0.844 | -0.020 [0.100] | -0.22 \| 0.18 | 0.924 (0.821) | -0.050 [0.100] | -0.25 \| 0.15 | 0.679 (0.604) |
| FC | 0.142 ± 0.667 | 0.111 ± 0.699 | -0.010 [0.040] | -0.09 \| 0.06 | 0.915 (0.720) | <0.001 [0.040] | -0.07 \| 0.07 | 0.988 (0.988) |
| **CC body** | | | | | | | | |
| FDC | 6.089 ± 1.226 | 6.015 ± 1.283 | -0.070 [0.150] | -0.37 \| 0.24 | 0.828 (0.670) | -0.100 [0.150] | -0.4 \| 0.2 | 0.761 (0.520) |
| FD | 5.986 ± 1.143 | 5.936 ± 1.245 | -0.050 [0.150] | -0.35 \| 0.25 | 0.924 (0.740) | -0.090 [0.150] | -0.39 \| 0.21 | 0.679 (0.556) |
| FC | 0.131 ± 0.667 | 0.118 ± 0.701 | <0.001 [0.040] | -0.07 \| 0.08 | 0.915 (0.915) | 0.020 [0.040] | -0.06 \| 0.09 | 0.949 (0.623) |
| **CC splenium** | | | | | | | | |
| FDC | 8.065 ± 1.092 | 8.1 ± 1.12 | 0.050 [0.130] | -0.21 \| 0.3 | 0.828 (0.723) | 0.060 [0.130] | -0.2 \| 0.31 | 0.761 (0.676) |
| FD | 7.939 ± 0.97 | 8.005 ± 1.033 | 0.070 [0.130] | -0.18 \| 0.31 | 0.918 (0.601) | 0.070 [0.130] | -0.18 \| 0.32 | 0.679 (0.592) |
| FC | 0.134 ± 0.64 | 0.106 ± 0.69 | -0.010 [0.030] | -0.08 \| 0.06 | 0.915 (0.753) | <0.001 [0.030] | -0.07 \| 0.07 | 0.988 (0.978) |
| **Dorsal cingulum bundle** | | | | | | | | |
| FDC | 4.215 ± 0.54 | 4.233 ± 0.64 | 0.020 [0.070] | -0.12 \| 0.16 | 0.828 (0.748) | 0.040 [0.070] | -0.1 \| 0.18 | 0.761 (0.566) |
| FD | 4.142 ± 0.488 | 4.176 ± 0.568 | 0.030 [0.070] | -0.1 \| 0.17 | 0.918 (0.612) | 0.050 [0.070] | -0.08 \| 0.18 | 0.679 (0.449) |
| FC | 0.138 ± 0.655 | 0.125 ± 0.656 | <0.001 [0.030] | -0.05 \| 0.06 | 0.915 (0.894) | 0.010 [0.030] | -0.05 \| 0.07 | 0.949 (0.738) |
| **Ventral cingulum bundle** | | | | | | | | |
| FDC | 4.009 ± 0.706 | 3.87 ± 0.76 | -0.130 [0.090] | -0.31 \| 0.04 | 0.634 (0.141) | -0.110 [0.090] | -0.29 \| 0.07 | 0.761 (0.221) |
| FD | 3.953 ± 0.698 | 3.814 ± 0.735 | -0.140 [0.090] | -0.32 \| 0.04 | 0.567 (0.126) | -0.120 [0.090] | -0.3 \| 0.06 | 0.679 (0.180) |
| FC | 0.117 ± 0.646 | 0.124 ± 0.688 | 0.020 [0.040] | -0.05 \| 0.1 | 0.915 (0.518) | 0.030 [0.040] | -0.04 \| 0.1 | 0.949 (0.428) |
| **Posterior thal rad** | | | | | | | | |
| FDC | 4.77 ± 0.499 | 4.741 ± 0.495 | -0.020 [0.050] | -0.12 \| 0.08 | 0.828 (0.671) | -0.020 [0.050] | -0.13 \| 0.08 | 0.761 (0.631) |
| FD | 4.698 ± 0.386 | 4.671 ± 0.391 | -0.030 [0.050] | -0.12 \| 0.07 | 0.918 (0.579) | -0.030 [0.050] | -0.13 \| 0.06 | 0.679 (0.493) |
| FC | 0.133 ± 0.641 | 0.124 ± 0.655 | 0.010 [0.030] | -0.05 \| 0.06 | 0.915 (0.779) | 0.010 [0.030] | -0.04 \| 0.07 | 0.949 (0.647) |
| **Sag Stratum** | | | | | | | | |
| FDC | 5.543 ± 0.694 | 5.516 ± 0.723 | -0.020 [0.080] | -0.17 \| 0.13 | 0.828 (0.828) | <0.001 [0.070] | -0.14 \| 0.15 | 0.965 (0.965) |
| FD | 5.454 ± 0.576 | 5.448 ± 0.596 | -0.010 [0.070] | -0.15 \| 0.14 | 0.936 (0.936) | <0.001 [0.070] | -0.14 \| 0.15 | 0.948 (0.948) |
| FC | 0.14 ± 0.636 | 0.1 ± 0.683 | -0.020 [0.030] | -0.09 \| 0.04 | 0.915 (0.491) | -0.020 [0.030] | -0.09 \| 0.05 | 0.949 (0.534) |
| **SLF** | | | | | | | | |
| FDC | 4.096 ± 0.488 | 4.106 ± 0.41 | 0.020 [0.050] | -0.08 \| 0.11 | 0.828 (0.728) | 0.020 [0.050] | -0.07 \| 0.12 | 0.761 (0.625) |
| FD | 4.021 ± 0.387 | 4.045 ± 0.314 | 0.020 [0.040] | -0.06 \| 0.11 | 0.918 (0.581) | 0.030 [0.040] | -0.06 \| 0.11 | 0.679 (0.534) |
| FC | 0.129 ± 0.651 | 0.129 ± 0.653 | 0.020 [0.030] | -0.04 \| 0.07 | 0.915 (0.549) | 0.030 [0.030] | -0.03 \| 0.08 | 0.949 (0.383) |
| **uncF** | | | | | | | | |
| FDC | 6.446 ± 0.932 | 6.184 ± 1.161 | -0.250 [0.120] | -0.5 \| -0.01 | 0.405 (0.045) | -0.240 [0.120] | -0.48 \| 0.01 | 0.522 (0.058) |
| FD | 6.339 ± 0.798 | 6.074 ± 1.041 | -0.260 [0.120] | -0.5 \| -0.03 | 0.243 (0.027) | -0.260 [0.120] | -0.49 \| -0.02 | 0.279 (0.031) |
| FC | 0.146 ± 0.676 | 0.144 ± 0.649 | 0.020 [0.030] | -0.04 \| 0.07 | 0.915 (0.612) | 0.020 [0.030] | -0.04 \| 0.08 | 0.949 (0.482) |
| False Discovery rate (FDR) significant differences are marked in bold. P<0.05, uncorrected significant results are underlined. *Corrected for intracranial volume, age, sex and education in years; Abbreviations: FDC = fiber density and cross-section, FD = fiber density, FC = fiber cross-section. , SLF = superior longitudinal fascicle, CC genu = genu of the corpus callosum, CC body = body of the corpus callosum, CC splenium = splenium of the corpus callosum. | | | | | | | | |

| **Supplementary Table 17 – SSRI/SNRI history mixed model analyses of fixel measures – random effects for site** | | | | | | |
| --- | --- | --- | --- | --- | --- | --- |
|  | **Group difference (crude model)** | | | **Group difference (adjusted model)*** | | |
|  | B [SE]  (* 10^-1^) | 95% CI  (* 10^-1^) | P (unc) | B [SE]  (* 10^-1^) | 95% CI  (* 10^-1^) | P (unc) |
| **CC genu** | | | | | | |
| FDC | -0.070 [0.110] | -0.28 \| 0.14 | 0.818 (0.521) | -0.080 [0.110] | -0.29 \| 0.13 | 0.796 (0.460) |
| FD | -0.050 [0.100] | -0.26 \| 0.15 | 0.818 (0.605) | -0.080 [0.100] | -0.28 \| 0.13 | 0.780 (0.459) |
| FC | -0.020 [0.040] | -0.1 \| 0.06 | 0.919 (0.644) | <0.001 [0.040] | -0.08 \| 0.07 | 0.962 (0.913) |
| **CC body** | | | | | | |
| FDC | -0.040 [0.160] | -0.35 \| 0.27 | 0.818 (0.818) | -0.060 [0.160] | -0.37 \| 0.25 | 0.796 (0.690) |
| FD | -0.030 [0.150] | -0.33 \| 0.27 | 0.856 (0.856) | -0.060 [0.150] | -0.36 \| 0.24 | 0.780 (0.698) |
| FC | <0.001 [0.040] | -0.08 \| 0.07 | 0.934 (0.907) | 0.010 [0.040] | -0.07 \| 0.09 | 0.962 (0.786) |
| **CC splenium** | | | | | | |
| FDC | 0.040 [0.140] | -0.23 \| 0.31 | 0.818 (0.762) | 0.050 [0.140] | -0.22 \| 0.32 | 0.796 (0.708) |
| FD | 0.060 [0.130] | -0.2 \| 0.32 | 0.818 (0.636) | 0.070 [0.130] | -0.19 \| 0.33 | 0.780 (0.613) |
| FC | -0.010 [0.040] | -0.08 \| 0.06 | 0.919 (0.715) | <0.001 [0.030] | -0.07 \| 0.07 | 0.962 (0.962) |
| **Dorsal cingulum** | | | | | | |
| FDC | 0.020 [0.080] | -0.13 \| 0.17 | 0.818 (0.793) | 0.040 [0.080] | -0.11 \| 0.19 | 0.796 (0.590) |
| FD | 0.020 [0.070] | -0.12 \| 0.16 | 0.856 (0.787) | 0.040 [0.070] | -0.1 \| 0.18 | 0.780 (0.561) |
| FC | 0.020 [0.030] | -0.04 \| 0.07 | 0.919 (0.581) | 0.020 [0.030] | -0.04 \| 0.08 | 0.848 (0.471) |
| **Ventral cingulum** | | | | | | |
| FDC | -0.140 [0.090] | -0.32 \| 0.05 | 0.661 (0.147) | -0.110 [0.090] | -0.29 \| 0.07 | 0.796 (0.236) |
| FD | -0.150 [0.090] | -0.33 \| 0.04 | 0.522 (0.116) | -0.120 [0.090] | -0.3 \| 0.06 | 0.780 (0.183) |
| FC | 0.040 [0.040] | -0.03 \| 0.12 | 0.919 (0.250) | 0.040 [0.040] | -0.03 \| 0.11 | 0.848 (0.247) |
| **Posterior thal rad** | | | | | | |
| FDC | -0.030 [0.050] | -0.14 \| 0.07 | 0.818 (0.516) | -0.030 [0.050] | -0.14 \| 0.07 | 0.796 (0.529) |
| FD | -0.040 [0.050] | -0.14 \| 0.06 | 0.818 (0.392) | -0.040 [0.050] | -0.14 \| 0.05 | 0.780 (0.375) |
| FC | 0.020 [0.030] | -0.04 \| 0.08 | 0.919 (0.472) | 0.020 [0.030] | -0.03 \| 0.08 | 0.848 (0.395) |
| **Sag Stratum** | | | | | | |
| FDC | -0.050 [0.080] | -0.2 \| 0.11 | 0.818 (0.558) | -0.020 [0.080] | -0.17 \| 0.13 | 0.830 (0.830) |
| FD | -0.040 [0.080] | -0.19 \| 0.11 | 0.818 (0.592) | -0.020 [0.070] | -0.17 \| 0.13 | 0.780 (0.780) |
| FC | <0.001 [0.030] | -0.07 \| 0.06 | 0.934 (0.934) | <0.001 [0.030] | -0.07 \| 0.06 | 0.962 (0.921) |
| **SLF** | | | | | | |
| FDC | 0.020 [0.050] | -0.08 \| 0.11 | 0.818 (0.731) | 0.020 [0.050] | -0.07 \| 0.12 | 0.796 (0.645) |
| FD | 0.020 [0.040] | -0.06 \| 0.11 | 0.818 (0.580) | 0.030 [0.050] | -0.06 \| 0.12 | 0.780 (0.544) |
| FC | 0.030 [0.030] | -0.03 \| 0.08 | 0.919 (0.386) | 0.030 [0.030] | -0.03 \| 0.09 | 0.848 (0.265) |
| **uncF** | | | | | | |
| FDC | -0.290 [0.130] | -0.54 \| -0.04 | 0.207 (0.023) | -0.270 [0.130] | -0.52 \| -0.02 | 0.324 (0.036) |
| FD | -0.300 [0.120] | -0.54 \| -0.07 | 0.117 (0.013) | -0.290 [0.120] | -0.53 \| -0.05 | 0.153 (0.017) |
| FC | 0.020 [0.030] | -0.04 \| 0.08 | 0.919 (0.470) | 0.030 [0.030] | -0.03 \| 0.09 | 0.848 (0.348) |
| False Discovery rate (FDR) significant differences are marked in bold. P<0.05, uncorrected significant results are underlined. *Corrected for age, sex and education in years; Abbreviations: SLF = superior longitudinal fascicle, CC genu = genu of the corpus callosum, CC body = body of the corpus callosum, CC splenium = splenium of the corpus callosum. | | | | | | |

| **Supplementary Table 18 – Mixed model analyses of fixel measures and YBOCS – ComBat corrected** | | | | | | |
| --- | --- | --- | --- | --- | --- | --- |
|  | **Group difference (crude model)** | | | **Group difference (adjusted model)*** | | |
|  | B [SE]  (* 10^-2^) | 95% CI  (* 10^-2^) | P (unc) | B [SE]  (* 10^-2^) | 95% CI  (* 10^-2^) | P (unc) |
| **CC genu** | | | | | | |
| FDC | 0.100 [0.100] | -0.2 \| 0.3 | 0.990 (0.640) | <0.001 [0.100] | -0.2 \| 0.2 | 0.999 (0.845) |
| FD | 0.100 [0.100] | -0.1 \| 0.3 | 0.905 (0.562) | <0.001 [0.100] | -0.2 \| 0.2 | 0.999 (0.787) |
| FC | <0.001 [<0.001] | -0.1 \| 0 | 0.798 (0.301) | <0.001 [<0.001] | -0.1 \| 0 | 0.657 (0.195) |
| **CC body** | | | | | | |
| FDC | <0.001 [0.200] | -0.3 \| 0.3 | 0.990 (0.908) | <0.001 [0.200] | -0.3 \| 0.3 | 0.999 (0.976) |
| FD | 0.100 [0.200] | -0.2 \| 0.4 | 0.905 (0.726) | <0.001 [0.200] | -0.3 \| 0.3 | 0.999 (0.858) |
| FC | <0.001 [<0.001] | -0.1 \| 0 | 0.798 (0.268) | -0.100 [<0.001] | -0.1 \| 0 | 0.657 (0.172) |
| **CC splenium** | | | | | | |
| FDC | -0.100 [0.100] | -0.3 \| 0.2 | 0.990 (0.542) | -0.100 [0.100] | -0.3 \| 0.2 | 0.999 (0.527) |
| FD | <0.001 [0.100] | -0.3 \| 0.2 | 0.905 (0.905) | <0.001 [0.100] | -0.3 \| 0.2 | 0.999 (0.724) |
| FC | <0.001 [<0.001] | -0.1 \| 0 | 0.798 (0.315) | <0.001 [<0.001] | -0.1 \| 0 | 0.657 (0.242) |
| **Dorsal cingulum** | | | | | | |
| FDC | <0.001 [0.100] | -0.1 \| 0.2 | 0.990 (0.930) | <0.001 [0.100] | -0.1 \| 0.2 | 0.999 (0.861) |
| FD | <0.001 [0.100] | -0.1 \| 0.2 | 0.905 (0.787) | <0.001 [0.100] | -0.1 \| 0.1 | 0.999 (0.999) |
| FC | <0.001 [<0.001] | -0.1 \| 0 | 0.798 (0.527) | <0.001 [<0.001] | -0.1 \| 0 | 0.657 (0.547) |
| **Ventral cingulum** | | | | | | |
| FDC | <0.001 [0.100] | -0.2 \| 0.2 | 0.990 (0.786) | <0.001 [0.100] | -0.2 \| 0.2 | 0.999 (0.815) |
| FD | <0.001 [0.100] | -0.1 \| 0.2 | 0.905 (0.692) | <0.001 [0.100] | -0.2 \| 0.2 | 0.999 (0.997) |
| FC | <0.001 [<0.001] | -0.1 \| 0.1 | 0.798 (0.633) | <0.001 [<0.001] | <0.001 \| 0.1 | 0.657 (0.411) |
| **Posterior thal rad** | | | | | | |
| FDC | <0.001 [0.100] | -0.1 \| 0.1 | 0.990 (0.501) | <0.001 [0.100] | -0.1 \| 0.1 | 0.999 (0.501) |
| FD | <0.001 [<0.001] | -0.1 \| 0.1 | 0.905 (0.468) | <0.001 [<0.001] | -0.1 \| 0.1 | 0.999 (0.396) |
| FC | <0.001 [<0.001] | <0.001 \| 0.1 | 0.798 (0.647) | <0.001 [<0.001] | <0.001 \| 0.1 | 0.657 (0.581) |
| **Sag Stratum** | | | | | | |
| FDC | 0.100 [0.100] | -0.1 \| 0.2 | 0.990 (0.359) | 0.100 [0.100] | -0.1 \| 0.2 | 0.999 (0.465) |
| FD | 0.100 [0.100] | -0.1 \| 0.2 | 0.905 (0.461) | <0.001 [0.100] | -0.1 \| 0.2 | 0.999 (0.735) |
| FC | <0.001 [<0.001] | -0.1 \| 0.1 | 0.798 (0.709) | <0.001 [<0.001] | <0.001 \| 0.1 | 0.657 (0.584) |
| **SLF** | | | | | | |
| FDC | <0.001 [<0.001] | -0.1 \| 0.1 | 0.990 (0.769) | <0.001 [<0.001] | -0.1 \| 0.1 | 0.999 (0.804) |
| FD | <0.001 [<0.001] | -0.1 \| 0.1 | 0.905 (0.641) | <0.001 [<0.001] | -0.1 \| 0.1 | 0.999 (0.669) |
| FC | <0.001 [<0.001] | -0.1 \| 0 | 0.798 (0.445) | <0.001 [<0.001] | -0.1 \| 0 | 0.657 (0.421) |
| **uncF** | | | | | | |
| FDC | <0.001 [0.100] | -0.3 \| 0.3 | 0.990 (0.990) | <0.001 [0.100] | -0.3 \| 0.3 | 0.999 (0.999) |
| FD | <0.001 [0.100] | -0.3 \| 0.2 | 0.905 (0.870) | <0.001 [0.100] | -0.3 \| 0.2 | 0.999 (0.823) |
| FC | <0.001 [<0.001] | -0.1 \| 0.1 | 0.908 (0.908) | <0.001 [<0.001] | -0.1 \| 0.1 | 0.947 (0.947) |
| False Discovery rate (FDR) significant differences are marked in bold. P<0.05, uncorrected significant results are underlined. *Corrected for age, sex and education in years; Abbreviations: SLF = superior longitudinal fascicle, CC genu = genu of the corpus callosum, CC body = body of the corpus callosum, CC splenium = splenium of the corpus callosum. | | | | | | |

| **Supplementary Table 19 – Mixed model analyses of fixel measures and illness duration - ComBat** | | | | | | |
| --- | --- | --- | --- | --- | --- | --- |
|  | **Group difference (crude model)** | | | **Group difference (adjusted model)*** | | |
|  | B [SE]  (* 10^-2^) | 95% CI  (* 10^-2^) | P (unc) | B [SE]  (* 10^-2^) | 95% CI  (* 10^-2^) | P (unc) |
| **CC genu** | | | | | | |
| FDC | -0.100 [<0.001] | -0.2 \| <0.001 | 0.349 (0.110) | <0.001 [0.100] | -0.1 \| 0.1 | 0.744 (0.695) |
| FD | -0.100 [<0.001] | -0.2 \| <0.001 | 0.264 (0.079) | <0.001 [0.100] | -0.1 \| 0.1 | 0.843 (0.695) |
| FC | <0.001 [<0.001] | <0.001 \| <0.001 | 0.806 (0.765) | <0.001 [<0.001] | -0.1 \| <0.001 | 0.919 (0.489) |
| **CC body** | | | | | | |
| FDC |  |  |  |  |  |  |
| FD | <0.001 [0.100] | -0.1 \| 0.1 | 0.843 (0.843) | 0.100 [0.100] | -0.1 \| 0.2 | 0.732 (0.244) |
| FC | <0.001 [<0.001] | <0.001 \| <0.001 | 0.806 (0.662) | <0.001 [<0.001] | -0.1 \| <0.001 | 0.919 (0.495) |
| **CC splenium** | | | | | | |
| FDC | <0.001 [0.100] | -0.1 \| 0.2 | 0.544 (0.484) | <0.001 [0.100] | -0.1 \| 0.2 | 0.744 (0.596) |
| FD | <0.001 [0.100] | -0.1 \| 0.1 | 0.637 (0.566) | <0.001 [0.100] | -0.1 \| 0.2 | 0.843 (0.545) |
| FC | <0.001 [<0.001] | <0.001 \| <0.001 | 0.806 (0.563) | <0.001 [<0.001] | <0.001 \| <0.001 | 0.919 (0.591) |
| **Dorsal cingulum** | | | | | | |
| FDC | <0.001 [<0.001] | <0.001 \| 0.1 | 0.349 (0.127) | <0.001 [<0.001] | -0.1 \| 0.1 | 0.744 (0.744) |
| FD | 0.100 [<0.001] | <0.001 \| 0.1 | 0.264 (0.088) | <0.001 [<0.001] | <0.001 \| 0.1 | 0.843 (0.570) |
| FC | <0.001 [<0.001] | <0.001 \| <0.001 | 0.806 (0.449) | <0.001 [<0.001] | <0.001 \| <0.001 | 0.919 (0.919) |
| **Ventral cingulum** | | | | | | |
| FDC | 0.100 [<0.001] | <0.001 \| 0.1 | 0.349 (0.155) | <0.001 [<0.001] | -0.1 \| 0.1 | 0.744 (0.642) |
| FD | <0.001 [<0.001] | <0.001 \| 0.1 | 0.605 (0.269) | <0.001 [<0.001] | -0.1 \| 0.1 | 0.843 (0.789) |
| FC | <0.001 [<0.001] | <0.001 \| 0.1 | 0.806 (0.132) | <0.001 [<0.001] | <0.001 \| <0.001 | 0.919 (0.542) |
| **Posterior thal rad** | | | | | | |
| FDC | <0.001 [<0.001] | <0.001 \| 0.1 | 0.525 (0.350) | <0.001 [<0.001] | <0.001 \| 0.1 | 0.744 (0.214) |
| FD | <0.001 [<0.001] | <0.001 \| 0.1 | 0.637 (0.498) | <0.001 [<0.001] | <0.001 \| 0.1 | 0.732 (0.234) |
| FC | <0.001 [<0.001] | <0.001 \| <0.001 | 0.806 (0.478) | <0.001 [<0.001] | <0.001 \| <0.001 | 0.919 (0.885) |
| **Sag Stratum** | | | | | | |
| FDC | <0.001 [<0.001] | <0.001 \| 0.1 | 0.517 (0.287) | <0.001 [<0.001] | -0.1 \| 0.1 | 0.744 (0.651) |
| FD | <0.001 [<0.001] | <0.001 \| 0.1 | 0.637 (0.402) | <0.001 [<0.001] | -0.1 \| 0.1 | 0.843 (0.843) |
| FC | <0.001 [<0.001] | <0.001 \| <0.001 | 0.806 (0.421) | <0.001 [<0.001] | <0.001 \| <0.001 | 0.919 (0.796) |
| **SLF** | | | | | | |
| FDC | <0.001 [<0.001] | <0.001 \| 0.1 | 0.544 (0.455) | <0.001 [<0.001] | <0.001 \| 0.1 | 0.744 (0.725) |
| FD | <0.001 [<0.001] | <0.001 \| 0.1 | 0.637 (0.439) | <0.001 [<0.001] | <0.001 \| 0.1 | 0.843 (0.559) |
| FC | <0.001 [<0.001] | <0.001 \| <0.001 | 0.806 (0.616) | <0.001 [<0.001] | <0.001 \| <0.001 | 0.919 (0.592) |
| **uncF** | | | | | | |
| FDC | 0.100 [0.100] | <0.001 \| 0.2 | 0.349 (0.055) | 0.100 [0.100] | <0.001 \| 0.2 | 0.744 (0.122) |
| FD | 0.100 [0.100] | <0.001 \| 0.2 | 0.264 (0.047) | 0.100 [0.100] | <0.001 \| 0.2 | 0.732 (0.089) |
| FC | <0.001 [<0.001] | <0.001 \| <0.001 | 0.806 (0.806) | <0.001 [<0.001] | <0.001 \| <0.001 | 0.919 (0.636) |
| False Discovery rate (FDR) significant differences are marked in bold. P<0.05, uncorrected significant results are underlined. *Corrected for age, sex and education in years; Abbreviations: SLF = superior longitudinal fascicle, CC genu = genu of the corpus callosum, CC body = body of the corpus callosum, CC splenium = splenium of the corpus callosum. | | | | | | |

| **Supplementary Table 20 – Mixed model analyses of fixel measures and age of onset as continuous measure - ComBat** | | | | | | | | |
| --- | --- | --- | --- | --- | --- | --- | --- | --- |
|  | **crude model** | | | **adjusted model*** | | | | |
|  | B [SE]  (* 10^-2^) | 95% CI  (* 10^-2^) | P (unc) | B [SE]  (* 10^-2^) | | | 95% CI  (* 10^-2^) | P (unc) |
| **CC genu** | | | | | | | | |
| FDC | <0.001 [0.100] | -0.1 \| 0.1 | 0.765 (0.414) | <0.001 [0.100] | | | -0.1 \| 0.1 | 0.962 (0.740) |
| FD | -0.100 [<0.001] | -0.1 \| <0.001 | 0.599 (0.317) | <0.001 [0.100] | | | -0.1 \| 0.1 | 0.893 (0.751) |
| FC | <0.001 [<0.001] | <0.001 \| <0.001 | 0.915 (0.776) | <0.001 [<0.001] | | | <0.001 \| <0.001 | 0.914 (0.680) |
| **CC body** | | | | | | | | |
| FDC | <0.001 [0.100] | -0.2 \| 0.1 | 0.765 (0.510) | <0.001 [0.100] | | -0.2 \| 0.1 | | 0.962 (0.871) |
| FD | -0.100 [0.100] | -0.2 \| 0.1 | 0.599 (0.466) | <0.001 [0.100] | | -0.2 \| 0.1 | | 0.893 (0.893) |
| FC | <0.001 [<0.001] | <0.001 \| <0.001 | 0.915 (0.751) | <0.001 [<0.001] | | <0.001 \| <0.001 | | 0.914 (0.658) |
| **CC splenium** | | | | | | | | |
| FDC | <0.001 [0.100] | -0.1 \| 0.1 | 0.891 (0.891) | <0.001 [0.100] | | -0.1 \| 0.1 | | 0.962 (0.791) |
| FD | <0.001 [0.100] | -0.1 \| 0.1 | 0.846 (0.752) | <0.001 [0.100] | | -0.1 \| 0.1 | | 0.893 (0.755) |
| FC | <0.001 [<0.001] | <0.001 \| <0.001 | 0.915 (0.511) | <0.001 [<0.001] | | <0.001 \| <0.001 | | 0.956 (0.954) |
| **Dorsal cingulum** | | | | | | | | |
| FDC | 0.100 [<0.001] | <0.001 \| 0.1 | 0.675 (0.079) | | <0.001 [<0.001] | <0.001 \| 0.1 | | 0.617 (0.204) |
| FD | 0.100 [<0.001] | <0.001 \| 0.1 | 0.450 (0.100) | | <0.001 [<0.001] | <0.001 \| 0.1 | | 0.486 (0.216) |
| FC | <0.001 [<0.001] | <0.001 \| <0.001 | 0.915 (0.915) | | <0.001 [<0.001] | <0.001 \| <0.001 | | 0.914 (0.710) |
| **Ventral cingulum** | | | | | | | | |
| FDC | <0.001 [<0.001] | -0.1 \| 0.1 | 0.807 (0.628) | | -0.100 [<0.001] | -0.1 \| <0.001 | | 0.617 (0.274) |
| FD | <0.001 [<0.001] | -0.1 \| 0.1 | 0.599 (0.418) | | -0.100 [<0.001] | -0.1 \| <0.001 | | 0.486 (0.190) |
| FC | <0.001 [<0.001] | <0.001 \| 0.1 | 0.915 (0.204) | | <0.001 [<0.001] | <0.001 \| 0.1 | | 0.914 (0.353) |
| **Posterior thal rad** | | | | | | | | |
| FDC | <0.001 [<0.001] | -0.1 \| <0.001 | 0.675 (0.150) | | <0.001 [<0.001] | | -0.1 \| <0.001 | 0.617 (0.179) |
| FD | <0.001 [<0.001] | -0.1 \| <0.001 | 0.450 (0.084) | | <0.001 [<0.001] | | -0.1 \| <0.001 | 0.486 (0.139) |
| FC | <0.001 [<0.001] | <0.001 \| <0.001 | 0.915 (0.699) | | <0.001 [<0.001] | | <0.001 \| <0.001 | 0.956 (0.956) |
| **Sag Stratum** | | | | | | | | |
| FDC | <0.001 [<0.001] | -0.1 \| <0.001 | 0.765 (0.475) | <0.001 [<0.001] | | -0.1 \| <0.001 | | 0.617 (0.208) |
| FD | <0.001 [<0.001] | -0.1 \| <0.001 | 0.599 (0.310) | <0.001 [<0.001] | | -0.1 \| <0.001 | | 0.486 (0.176) |
| FC | <0.001 [<0.001] | <0.001 \| <0.001 | 0.915 (0.414) | <0.001 [<0.001] | | <0.001 \| <0.001 | | 0.914 (0.567) |
| **SLF** | | | | | | | | |
| FDC | <0.001 [<0.001] | <0.001 \| 0.1 | 0.891 (0.800) | <0.001 [<0.001] | | | <0.001 \| <0.001 | 0.962 (0.962) |
| FD | <0.001 [<0.001] | <0.001 \| <0.001 | 0.994 (0.994) | <0.001 [<0.001] | | | <0.001 \| <0.001 | 0.893 (0.884) |
| FC | <0.001 [<0.001] | <0.001 \| <0.001 | 0.915 (0.824) | <0.001 [<0.001] | | | <0.001 \| <0.001 | 0.914 (0.711) |
| **uncF** | | | | | | | | |
| FDC | <0.001 [0.100] | -0.1 \| 0.2 | 0.765 (0.455) | <0.001 [0.100] | | | -0.1 \| 0.2 | 0.962 (0.612) |
| FD | <0.001 [0.100] | -0.1 \| 0.2 | 0.599 (0.406) | <0.001 [0.100] | | | -0.1 \| 0.2 | 0.875 (0.486) |
| FC | <0.001 [<0.001] | <0.001 \| <0.001 | 0.915 (0.584) | <0.001 [<0.001] | | | <0.001 \| <0.001 | 0.914 (0.324) |
| False Discovery rate (FDR) significant differences are marked in bold. P<0.05, uncorrected significant results are underlined. *Corrected for age, sex and education in years; Abbreviations: SLF = superior longitudinal fascicle, CC genu = genu of the corpus callosum, CC body = body of the corpus callosum, CC splenium = splenium of the corpus callosum. | | | | | | | | |

| **Supplementary Table 21 - Associations between fixel measures and D-YBOCS subscales** | | | | | | | |
| --- | --- | --- | --- | --- | --- | --- | --- |
|  |  | **crude model** | | | **adjusted model*** | | |
|  | subscale | B [SE]  (* 10^-1^) | 95% CI  (* 10^-1^) | P | B [SE]  (* 10^-1^) | 95% CI  (* 10^-1^) | P |
| **CC genu** | | | | | | | |
| FDC | aggressive | 0.010 [0.020] | -0.03 \| 0.06 | 0.519 | 0.010 [0.020] | -0.04 \| 0.05 | 0.793 |
|  | sexual | -0.010 [0.020] | -0.06 \| 0.04 | 0.727 | -0.010 [0.020] | -0.06 \| 0.03 | 0.599 |
|  | symmetry | 0.030 [0.020] | -0.01 \| 0.07 | 0.124 | 0.030 [0.020] | -0.01 \| 0.07 | 0.127 |
|  | contamination | -0.020 [0.020] | -0.07 \| 0.02 | 0.283 | -0.020 [0.020] | -0.07 \| 0.02 | 0.241 |
|  | hoarding | 0.020 [0.040] | -0.05 \| 0.1 | 0.545 | 0.040 [0.040] | -0.04 \| 0.12 | 0.356 |
| FD | aggressive | 0.020 [0.020] | -0.02 \| 0.06 | 0.332 | 0.010 [0.020] | -0.03 \| 0.05 | 0.570 |
|  | sexual | -0.010 [0.020] | -0.06 \| 0.03 | 0.531 | -0.020 [0.020] | -0.06 \| 0.03 | 0.388 |
|  | symmetry | 0.030 [0.020] | -0.01 \| 0.07 | 0.090 | 0.030 [0.020] | -0.01 \| 0.07 | 0.116 |
|  | contamination | -0.030 [0.020] | -0.07 \| 0.01 | 0.193 | -0.030 [0.020] | -0.07 \| 0.01 | 0.108 |
|  | hoarding | 0.020 [0.040] | -0.06 \| 0.09 | 0.637 | 0.040 [0.040] | -0.04 \| 0.11 | 0.352 |
| FC | aggressive | -0.010 [0.010] | -0.03 \| <0.001 | 0.180 | -0.010 [0.010] | -0.03 \| <0.001 | 0.123 |
|  | sexual | 0.010 [0.010] | -0.01 \| 0.03 | 0.219 | 0.010 [0.010] | <0.001 \| 0.03 | 0.131 |
|  | symmetry | -0.010 [0.010] | -0.02 \| 0.01 | 0.409 | -0.010 [0.010] | -0.02 \| 0.01 | 0.447 |
|  | contamination | <0.001 [0.010] | -0.01 \| 0.02 | 0.578 | <0.001 [0.010] | -0.01 \| 0.02 | 0.633 |
|  | hoarding | 0.010 [0.010] | -0.02 \| 0.04 | 0.371 | <0.001 [0.010] | -0.03 \| 0.03 | 0.983 |
| **CC body** | | | | | | | |
| FDC | aggressive | 0.020 [0.030] | -0.04 \| 0.08 | 0.529 | 0.010 [0.030] | -0.05 \| 0.08 | 0.646 |
|  | sexual | -0.020 [0.040] | -0.09 \| 0.05 | 0.570 | -0.030 [0.040] | -0.1 \| 0.04 | 0.448 |
|  | symmetry | 0.040 [0.030] | -0.02 \| 0.1 | 0.185 | 0.040 [0.030] | -0.02 \| 0.1 | 0.202 |
|  | contamination | -0.060 [0.030] | -0.12 \| <0.001 | 0.054 | -0.060 [0.030] | -0.12 \| <0.001 | 0.049 |
|  | hoarding | 0.020 [0.060] | -0.09 \| 0.13 | 0.691 | 0.050 [0.060] | -0.06 \| 0.17 | 0.362 |
| FD | aggressive | 0.030 [0.030] | -0.03 \| 0.1 | 0.283 | 0.030 [0.030] | -0.04 \| 0.09 | 0.410 |
|  | sexual | -0.030 [0.030] | -0.09 \| 0.04 | 0.444 | -0.030 [0.030] | -0.1 \| 0.03 | 0.324 |
|  | symmetry | 0.050 [0.030] | -0.01 \| 0.1 | 0.134 | 0.040 [0.030] | -0.02 \| 0.1 | 0.158 |
|  | contamination | -0.060 [0.030] | -0.12 \| <0.001 | 0.062 | -0.060 [0.030] | -0.12 \| <0.001 | 0.046 |
|  | hoarding | 0.020 [0.060] | -0.08 \| 0.13 | 0.657 | 0.060 [0.060] | -0.06 \| 0.17 | 0.330 |
| FC | aggressive | -0.010 [0.010] | -0.03 \| <0.001 | 0.081 | -0.020 [0.010] | -0.03 \| <0.001 | 0.052 |
|  | sexual | 0.010 [0.010] | <0.001\| 0.03 | 0.150 | 0.020 [0.010] | <0.001\| 0.03 | 0.077 |
|  | symmetry | -0.010 [0.010] | -0.02 \| 0.01 | 0.311 | -0.010 [0.010] | -0.02 \| 0.01 | 0.343 |
|  | contamination | <0.001 [0.010] | -0.01 \| 0.02 | 0.765 | <0.001 [0.010] | -0.01 \| 0.02 | 0.827 |
|  | hoarding | 0.020 [0.010] | -0.01 \| 0.04 | 0.289 | <0.001 [0.010] | -0.03 \| 0.03 | 0.960 |
| **CC splenium** | | | | | | | |
| FDC | aggressive | 0.010 [0.030] | -0.04 \| 0.06 | 0.653 | 0.010 [0.030] | -0.04 \| 0.06 | 0.701 |
|  | sexual | -0.020 [0.030] | -0.07 \| 0.04 | 0.575 | -0.020 [0.030] | -0.07 \| 0.04 | 0.577 |
|  | symmetry | -0.010 [0.030] | -0.06 \| 0.04 | 0.602 | -0.010 [0.030] | -0.06 \| 0.04 | 0.603 |
|  | contamination | -0.060 [0.030] | -0.11 \| -0.01 | 0.018 | -0.060 [0.030] | -0.11 \| -0.01 | 0.020 |
|  | hoarding | 0.050 [0.050] | -0.05 \| 0.14 | 0.339 | 0.040 [0.050] | -0.05 \| 0.14 | 0.384 |
| FD | aggressive | 0.030 [0.030] | -0.02 \| 0.08 | 0.311 | 0.020 [0.030] | -0.03 \| 0.07 | 0.426 |
|  | sexual | -0.020 [0.030] | -0.08 \| 0.03 | 0.390 | -0.030 [0.030] | -0.08 \| 0.03 | 0.375 |
|  | symmetry | <0.001 [0.020] | -0.05 \| 0.05 | 0.973 | <0.001 [0.020] | -0.05 \| 0.05 | 0.905 |
|  | contamination | -0.060 [0.020] | -0.1 \| -0.01 | 0.027 | -0.060 [0.030] | -0.11 \| -0.01 | 0.017 |
|  | hoarding | 0.050 [0.050] | -0.04 \| 0.14 | 0.257 | 0.050 [0.050] | -0.05 \| 0.14 | 0.311 |
| FC | aggressive | -0.010 [0.010] | -0.03 \| <0.001 | 0.068 | -0.010 [0.010] | -0.03 \| <0.001 | 0.054 |
|  | sexual | 0.010 [0.010] | -0.01 \| 0.03 | 0.192 | 0.010 [0.010] | <0.001\| 0.03 | 0.115 |
|  | symmetry | -0.010 [0.010] | -0.02 \| 0.01 | 0.221 | -0.010 [0.010] | -0.02 \| 0.01 | 0.246 |
|  | contamination | 0.010 [0.010] | -0.01 \| 0.02 | 0.464 | <0.001 [0.010] | -0.01 \| 0.02 | 0.511 |
|  | hoarding | 0.010 [0.010] | -0.02 \| 0.03 | 0.462 | <0.001 [0.010] | -0.03 \| 0.02 | 0.908 |
| **Dorsal cingulum** | | | | | | | |
| FDC | aggressive | 0.010 [0.010] | -0.02 \| 0.04 | 0.706 | 0.010 [0.010] | -0.02 \| 0.04 | 0.582 |
|  | sexual | 0.010 [0.020] | -0.02 \| 0.04 | 0.585 | 0.010 [0.020] | -0.02 \| 0.05 | 0.413 |
|  | symmetry | 0.010 [0.010] | -0.01 \| 0.04 | 0.336 | 0.010 [0.010] | -0.01 \| 0.04 | 0.296 |
|  | contamination | -0.020 [0.010] | -0.04 \| 0.01 | 0.266 | -0.010 [0.010] | -0.04 \| 0.01 | 0.322 |
|  | hoarding | 0.030 [0.030] | -0.03 \| 0.08 | 0.344 | <0.001 [0.030] | -0.05 \| 0.06 | 0.939 |
| FD | aggressive | 0.010 [0.010] | -0.02 \| 0.04 | 0.533 | 0.010 [0.010] | -0.02 \| 0.04 | 0.549 |
|  | sexual | 0.010 [0.020] | -0.02 \| 0.04 | 0.700 | 0.010 [0.010] | -0.02 \| 0.04 | 0.496 |
|  | symmetry | 0.010 [0.010] | -0.01 \| 0.04 | 0.306 | 0.010 [0.010] | -0.01 \| 0.04 | 0.318 |
|  | contamination | -0.010 [0.010] | -0.04 \| 0.01 | 0.291 | -0.020 [0.010] | -0.04 \| 0.01 | 0.209 |
|  | hoarding | 0.030 [0.020] | -0.02 \| 0.08 | 0.204 | <0.001 [0.030] | -0.05 \| 0.05 | 0.880 |
| FC | aggressive | -0.010 [0.010] | -0.02 \| 0.01 | 0.395 | <0.001 [0.010] | -0.02 \| 0.01 | 0.450 |
|  | sexual | 0.010 [0.010] | -0.01 \| 0.02 | 0.411 | 0.010 [0.010] | -0.01 \| 0.02 | 0.323 |
|  | symmetry | <0.001 [0.010] | -0.01 \| 0.01 | 0.688 | <0.001 [0.010] | -0.01 \| 0.01 | 0.650 |
|  | contamination | 0.010 [0.010] | -0.01 \| 0.02 | 0.349 | 0.010 [0.010] | -0.01 \| 0.02 | 0.351 |
|  | hoarding | 0.010 [0.010] | -0.02 \| 0.03 | 0.591 | <0.001 [0.010] | -0.02 \| 0.02 | 0.974 |
| **Ventral cingulum** | | | | | | | |
| FDC | aggressive | -0.020 [0.020] | -0.06 \| 0.01 | 0.242 | -0.020 [0.020] | -0.06 \| 0.01 | 0.241 |
|  | sexual | -0.010 [0.020] | -0.05 \| 0.03 | 0.549 | -0.010 [0.020] | -0.05 \| 0.03 | 0.692 |
|  | symmetry | -0.040 [0.020] | -0.07 \| <0.001 | 0.032 | -0.040 [0.020] | -0.07 \| <0.001 | 0.036 |
|  | contamination | -0.020 [0.020] | -0.05 \| 0.02 | 0.383 | -0.020 [0.020] | -0.05 \| 0.02 | 0.380 |
|  | hoarding | 0.020 [0.030] | -0.04 \| 0.09 | 0.522 | <0.001 [0.030] | -0.07 \| 0.07 | 0.962 |
| FD | aggressive | -0.010 [0.020] | -0.05 \| 0.02 | 0.464 | -0.020 [0.020] | -0.05 \| 0.02 | 0.345 |
|  | sexual | -0.010 [0.020] | -0.05 \| 0.03 | 0.487 | -0.010 [0.020] | -0.05 \| 0.03 | 0.609 |
|  | symmetry | -0.040 [0.020] | -0.07 \| <0.001 | 0.046 | -0.040 [0.020] | -0.07 \| <0.001 | 0.037 |
|  | contamination | -0.010 [0.020] | -0.05 \| 0.02 | 0.407 | -0.020 [0.020] | -0.06 \| 0.01 | 0.242 |
|  | hoarding | 0.030 [0.030] | -0.03 \| 0.09 | 0.358 | <0.001 [0.030] | -0.06 \| 0.07 | 0.924 |
| FC | aggressive | -0.020 [0.010] | -0.03 \| <0.001 | 0.044 | -0.010 [0.010] | -0.03 \| <0.001 | 0.089 |
|  | sexual | <0.001 [0.010] | -0.01 \| 0.02 | 0.625 | <0.001 [0.010] | -0.01 \| 0.02 | 0.566 |
|  | symmetry | <0.001 [0.010] | -0.02 \| 0.01 | 0.513 | <0.001 [0.010] | -0.02 \| 0.01 | 0.514 |
|  | contamination | 0.010 [0.010] | <0.001 \| 0.03 | 0.139 | 0.010 [0.010] | <0.001 \| 0.02 | 0.129 |
|  | hoarding | <0.001 [0.010] | -0.03 \| 0.02 | 0.836 | <0.001 [0.010] | -0.03 \| 0.02 | 0.856 |
| **Post Thal Rad** | | | | | | | |
| FDC | aggressive | -0.010 [0.010] | -0.03 \| 0.01 | 0.397 | -0.010 [0.010] | -0.03 \| 0.01 | 0.366 |
|  | sexual | 0.010 [0.010] | -0.01 \| 0.03 | 0.366 | 0.010 [0.010] | -0.01 \| 0.03 | 0.371 |
|  | symmetry | 0.020 [0.010] | <0.001 \| 0.04 | 0.017 | 0.020 [0.010] | <0.001 \| 0.04 | 0.018 |
|  | contamination | -0.020 [0.010] | -0.04 \| <0.001 | 0.057 | -0.020 [0.010] | -0.04 \| <0.001 | 0.067 |
|  | hoarding | -0.020 [0.020] | -0.06 \| 0.01 | 0.243 | -0.020 [0.020] | -0.06 \| 0.02 | 0.234 |
| FD | aggressive | -0.010 [0.010] | -0.02 \| 0.01 | 0.591 | -0.010 [0.010] | -0.03 \| 0.01 | 0.469 |
|  | sexual | 0.010 [0.010] | -0.02 \| 0.03 | 0.579 | 0.010 [0.010] | -0.02 \| 0.03 | 0.608 |
|  | symmetry | 0.020 [0.010] | <0.001\| 0.04 | 0.016 | 0.020 [0.010] | <0.001\| 0.04 | 0.020 |
|  | contamination | -0.020 [0.010] | -0.04 \| <0.001 | 0.028 | -0.020 [0.010] | -0.04 \| <0.001 | 0.019 |
|  | hoarding | -0.020 [0.020] | -0.05 \| 0.02 | 0.275 | -0.020 [0.020] | -0.06 \| 0.02 | 0.265 |
| FC | aggressive | <0.001 [0.010] | -0.02 \| 0.01 | 0.425 | <0.001 [0.010] | -0.02 \| 0.01 | 0.516 |
|  | sexual | 0.010 [0.010] | -0.01 \| 0.02 | 0.354 | 0.010 [0.010] | -0.01 \| 0.02 | 0.285 |
|  | symmetry | <0.001 [0.010] | -0.01 \| 0.01 | 0.714 | <0.001 [0.010] | -0.01 \| 0.01 | 0.681 |
|  | contamination | 0.010 [0.010] | <0.001 \| 0.02 | 0.208 | 0.010 [0.010] | <0.001 \| 0.02 | 0.210 |
|  | hoarding | <0.001 [0.010] | -0.02 \| 0.02 | 0.903 | <0.001 [0.010] | -0.02 \| 0.02 | 0.799 |
| **Sag Stratum** | | | | | | | |
| FDC | aggressive | 0.010 [0.020] | -0.02 \| 0.04 | 0.500 | 0.010 [0.020] | -0.02 \| 0.04 | 0.617 |
|  | sexual | 0.030 [0.020] | <0.001 \| 0.06 | 0.092 | 0.030 [0.020] | <0.001 \| 0.07 | 0.049 |
|  | symmetry | 0.010 [0.020] | -0.02 \| 0.04 | 0.692 | 0.010 [0.010] | -0.02 \| 0.04 | 0.630 |
|  | contamination | -0.030 [0.020] | -0.06 \| <0.001 | 0.032 | -0.030 [0.010] | -0.06 \| <0.001 | 0.028 |
|  | hoarding | 0.030 [0.030] | -0.03 \| 0.08 | 0.338 | <0.001 [0.030] | -0.05 \| 0.06 | 0.973 |
| FD | aggressive | 0.010 [0.010] | -0.02 \| 0.04 | 0.384 | 0.010 [0.010] | -0.02 \| 0.04 | 0.563 |
|  | sexual | 0.030 [0.020] | -0.01 \| 0.06 | 0.103 | 0.030 [0.020] | <0.001 \| 0.06 | 0.061 |
|  | symmetry | 0.010 [0.010] | -0.02 \| 0.03 | 0.681 | <0.001 [0.010] | -0.02 \| 0.03 | 0.756 |
|  | contamination | -0.040 [0.010] | -0.07 \| -0.01 | 0.008 | -0.040 [0.010] | -0.07 \| -0.02 | 0.002 |
|  | hoarding | 0.030 [0.030] | -0.02 \| 0.08 | 0.261 | <0.001 [0.030] | -0.05 \| 0.06 | 0.890 |
| FC | aggressive | -0.010 [0.010] | -0.02 \| 0.01 | 0.438 | <0.001 [0.010] | -0.02 \| 0.01 | 0.562 |
|  | sexual | <0.001 [0.010] | -0.01 \| 0.02 | 0.737 | <0.001 [0.010] | -0.01 \| 0.02 | 0.704 |
|  | symmetry | <0.001 [0.010] | -0.01 \| 0.01 | 0.978 | <0.001 [0.010] | -0.01 \| 0.01 | 0.990 |
|  | contamination | 0.010 [0.010] | <0.001 \| 0.02 | 0.116 | 0.010 [0.010] | <0.001 \| 0.02 | 0.120 |
|  | hoarding | -0.010 [0.010] | -0.03 \| 0.02 | 0.607 | -0.010 [0.010] | -0.03 \| 0.02 | 0.628 |
| **SLF** | | | | | | | |
| FDC | aggressive | <0.001 [0.010] | -0.02 \| 0.02 | 0.899 | <0.001 [0.010] | -0.02 \| 0.02 | 0.857 |
|  | sexual | <0.001 [0.010] | -0.02 \| 0.02 | 0.912 | <0.001 [0.010] | -0.02 \| 0.02 | 0.888 |
|  | symmetry | <0.001 [0.010] | -0.02 \| 0.02 | 0.743 | <0.001 [0.010] | -0.02 \| 0.02 | 0.734 |
|  | contamination | -0.010 [0.010] | -0.02 \| 0.01 | 0.551 | -0.010 [0.010] | -0.03 \| 0.01 | 0.519 |
|  | hoarding | 0.030 [0.020] | -0.01 \| 0.06 | 0.102 | 0.030 [0.020] | -0.01 \| 0.06 | 0.145 |
| FD | aggressive | <0.001 [0.010] | -0.02 \| 0.02 | 0.849 | <0.001 [0.010] | -0.02 \| 0.02 | 0.868 |
|  | sexual | <0.001 [0.010] | -0.02 \| 0.02 | 0.750 | <0.001 [0.010] | -0.02 \| 0.02 | 0.736 |
|  | symmetry | <0.001 [0.010] | -0.01 \| 0.02 | 0.575 | <0.001 [0.010] | -0.01 \| 0.02 | 0.578 |
|  | contamination | -0.010 [0.010] | -0.03 \| 0.01 | 0.302 | -0.010 [0.010] | -0.03 \| 0.01 | 0.304 |
|  | hoarding | 0.030 [0.020] | <0.001 \| 0.06 | 0.071 | 0.030 [0.020] | <0.001 \| 0.06 | 0.075 |
| FC | aggressive | -0.010 [0.010] | -0.02 \| <0.001 | 0.213 | -0.010 [0.010] | -0.02 \| <0.001 | 0.226 |
|  | sexual | 0.010 [0.010] | -0.01 \| 0.02 | 0.290 | 0.010 [0.010] | <0.001 \| 0.02 | 0.203 |
|  | symmetry | <0.001 [0.010] | -0.01 \| 0.01 | 0.820 | <0.001 [0.010] | -0.01 \| 0.01 | 0.767 |
|  | contamination | <0.001 [0.010] | -0.01 \| 0.02 | 0.505 | <0.001 [0.010] | -0.01 \| 0.02 | 0.517 |
|  | hoarding | 0.010 [0.010] | -0.01 \| 0.03 | 0.286 | <0.001 [0.010] | -0.02 \| 0.03 | 0.714 |
| **uncF** | | | | | | | |
| FDC | aggressive | -0.030 [0.030] | -0.08 \| 0.02 | 0.265 | -0.030 [0.030] | -0.08 \| 0.02 | 0.258 |
|  | sexual | -0.050 [0.030] | -0.11 \| <0.001 | 0.067 | -0.050 [0.030] | -0.1 \| 0.01 | 0.088 |
|  | symmetry | <0.001 [0.020] | -0.05 \| 0.05 | 0.976 | <0.001 [0.020] | -0.05 \| 0.05 | 0.950 |
|  | contamination | 0.010 [0.030] | -0.04 \| 0.06 | 0.621 | 0.010 [0.030] | -0.04 \| 0.06 | 0.567 |
|  | hoarding | 0.080 [0.050] | -0.01 \| 0.17 | 0.070 | 0.060 [0.050] | -0.03 \| 0.16 | 0.204 |
| FD | aggressive | -0.030 [0.020] | -0.07 \| 0.02 | 0.293 | -0.030 [0.030] | -0.08 \| 0.02 | 0.294 |
|  | sexual | -0.060 [0.030] | -0.11 \| <0.001 | 0.042 | -0.050 [0.030] | -0.11 \| <0.001 | 0.053 |
|  | symmetry | <0.001 [0.020] | -0.05 \| 0.04 | 0.874 | <0.001 [0.020] | -0.05 \| 0.04 | 0.852 |
|  | contamination | 0.010 [0.020] | -0.04 \| 0.05 | 0.778 | 0.010 [0.020] | -0.04 \| 0.05 | 0.820 |
|  | hoarding | 0.070 [0.040] | -0.01 \| 0.16 | 0.101 | 0.060 [0.050] | -0.03 \| 0.15 | 0.223 |
| FC | aggressive | <0.001 [0.010] | -0.01 \| 0.01 | 0.707 | <0.001 [0.010] | -0.01 \| 0.01 | 0.706 |
|  | sexual | 0.010 [0.010] | -0.01 \| 0.02 | 0.278 | 0.010 [0.010] | <0.001 \| 0.02 | 0.211 |
|  | symmetry | 0.010 [0.010] | -0.01 \| 0.02 | 0.360 | 0.010 [0.010] | -0.01 \| 0.02 | 0.332 |
|  | contamination | <0.001 [0.010] | -0.01 \| 0.02 | 0.502 | <0.001 [0.010] | -0.01 \| 0.02 | 0.503 |
|  | hoarding | 0.010 [0.010] | -0.02 \| 0.03 | 0.598 | <0.001 [0.010] | -0.02 \| 0.02 | 0.962 |
| Significant differences are marked in bold. *Corrected for age, sex and education in years; Abbreviations: SLF = superior longitudinal fascicle, CC genu = genu of the corpus callosum, CC body = body of the corpus callosum, CC splenium = splenium of the corpus callosum. | | | | | | | |

**CONNECTOME MEASURES**

| **Supplementary Table 22 – Case control mixed model analyses of global and nodal connectome measures – ComBat Corrected** | | | | | | | | |
| --- | --- | --- | --- | --- | --- | --- | --- | --- |
|  | **HC** | **OCD** | **Group difference (crude model)#** | | | **Group difference (adjusted model)*** | | |
|  | M ± SD | M ± SD | B [SE] | 95% CI | P (unc) | B [SE] | 95% CI | P (unc) |
| **Global measures** | | | | | | | | |
| Small-worldness | 1.848 ± 0.164 | 1.826 ± 0.164 | -2.200 [1.400] | -5.0 \| 0.7 | 0.132 | -2.600 [1.400] | -5.4 \| 0.3 | 0.076 |
| Q | 0.58 ± 0.014 | 0.579 ± 0.013 | -0.200 [0.100] | -0.4 \| 0.1 | 0.122 | -0.200 [0.100] | -0.5 \| 0.0 | 0.054 |
| Global efficiency  (x 10^-2)^ | 2.9 ± 0.5 | 2.9 ± 0.5 | 1.400 [4.500] | -7.4 \| 10.2 | 0.753 | 0.500 [4.500] | -8.4 \| 9.5 | 0.904 |
| RC coefficient | 1.138 ± 0.201 | 1.127 ± 0.224 | -1.100 [1.900] | -4.8 \| 2.6 | 0.559 | -0.900 [1.900] | -4.6 \| 2.8 | 0.631 |
| **Nodal measures (x 10^-2^)** | | | | | | | | |
| **Thalamus** | | | | | | | | |
| Eloc | 0.618 ± 0.111 | 0.632 ± 0.12 | 0.014 [0.010] | -0.01 \| 0.03 | 0.848 (0.181) | 0.012 [0.010] | -0.01 \| 0.03 | 0.986 (0.263) |
| BC | 36.88 ± 7.149 | 36.64 ± 6.956 | -0.240 [0.620] | -1.47 \| 0.98 | 0.843 (0.700) | -0.412 [0.610] | -1.61 \| 0.79 | 0.835 (0.501) |
| **Amygdala** | | | | | | | | |
| Eloc | 0.207 ± 0.061 | 0.204 ± 0.063 | -0.003 [0.000] | -0.01 \| 0.01 | 0.848 (0.588) | -0.004 [0.010] | -0.01 \| 0.01 | 0.986 (0.502) |
| BC | 1.719 ± 1.132 | 1.598 ± 1.14 | -0.121 [0.100] | -0.32 \| 0.08 | 0.755 (0.229) | -0.116 [0.100] | -0.31 \| 0.08 | 0.630 (0.252) |
| **Putamen** | | | | | | | | |
| Eloc | 0.525 ± 0.098 | 0.528 ± 0.105 | 0.002 [0.010] | -0.01 \| 0.02 | 0.848 (0.803) | 0.000 [0.010] | -0.02 \| 0.02 | 0.986 (0.986) |
| BC | 8.553 ± 3.889 | 8.193 ± 3.999 | -0.360 [0.350] | -1.04 \| 0.32 | 0.755 (0.302) | -0.444 [0.350] | -1.13 \| 0.24 | 0.630 (0.204) |
| **Pallidum** | | | | | | | | |
| Eloc | 0.341 ± 0.071 | 0.344 ± 0.08 | 0.003 [0.010] | -0.01 \| 0.02 | 0.848 (0.639) | 0.002 [0.010] | -0.01 \| 0.01 | 0.986 (0.782) |
| BC | 0.472 ± 0.703 | 0.442 ± 1.083 | -0.030 [0.080] | -0.19 \| 0.13 | 0.843 (0.709) | -0.021 [0.080] | -0.18 \| 0.14 | 0.858 (0.797) |
| **Hippocampus** | | | | | | | | |
| Eloc | 0.338 ± 0.067 | 0.339 ± 0.07 | 0.001 [0.010] | -0.01 \| 0.01 | 0.848 (0.848) | -0.001 [0.010] | -0.01 \| 0.01 | 0.986 (0.932) |
| BC | 9.361 ± 3.204 | 9.301 ± 3.539 | -0.059 [0.300] | -0.65 \| 0.53 | 0.843 (0.843) | -0.054 [0.300] | -0.65 \| 0.54 | 0.858 (0.858) |
| Reported connectome measures are adjusted for site using Combat. Significant differences are marked in bold. *Corrected for age, sex and education in years; Abbreviations: Q = modularity, RC = Rich Club, Eloc = local efficiency, BC = betweenness centrality. | | | | | | | | |

| **Supplementary Table 23 – Age of OCD onset mixed model analyses of global and nodal connectome measures – ComBat corrected** | | | | | | | | |
| --- | --- | --- | --- | --- | --- | --- | --- | --- |
|  | **ADULT** | **CHILD** | **Group difference (crude model)#** | | | **Group difference (adjusted model)*** | | |
|  | M ± SD | M ± SD | B [SE] | 95% CI | P | B [SE] | 95% CI | P |
| **Global measures** | | | | | | | | |
| Small-worldness | 1.828 ± 0.153 | 1.823 ± 0.172 | -0.500 [2.100] | -4.5 \| 3.5 | 0.808 | -1.900 [2.000] | -6 \| 2.1 | 0.345 |
| Q | 0.578 ± 0.012 | 0.579 ± 0.014 | 0.100 [0.200] | -0.3 \| 0.4 | 0.702 | -0.100 [0.200] | -0.4 \| 0.2 | 0.452 |
| Global efficiency  (x 10^-2)^ | 2.8 ± 0.5 | 3.0 ± 0.5 | 15.300 [6.600] | 2.3 \| 28.2 | **0.021** | 14.800 [6.800] | 1.5 \| 28.1 | **0.029** |
| RC coefficient | 1.109 ± 0.223 | 1.139 ± 0.225 | 3.100 [2.800] | -2.5 \| 8.6 | 0.276 | 2.400 [2.900] | -3.3 \| 8 | 0.412 |
| **Nodal measures (x 10^-2^)** | | | | | | | | |
| **Thalamus** | | | | | | | | |
| Eloc | 0.612 ± 0.106 | 0.648 ± 0.129 | 0.036 [0.010] | 0.01 \| 0.07 | 0.080 (0.016) | 0.037 [0.010] | 0.01 \| 0.07 | 0.090 (0.018) |
| BC | 36.276 ± 6.722 | 36.919 ± 7.169 | 0.643 [0.870] | -1.08 \| 2.36 | 0.578 (0.462) | 0.163 [0.870] | -1.56 \| 1.88 | 0.908 (0.853) |
| **Amygdala** | | | | | | | | |
| Eloc | 0.203 ± 0.063 | 0.206 ± 0.063 | 0.003 [0.010] | -0.01 \| 0.02 | 0.699 (0.699) | 0.003 [0.010] | -0.01 \| 0.02 | 0.676 (0.676) |
| BC | 1.591 ± 1.125 | 1.613 ± 1.152 | 0.023 [0.140] | -0.26 \| 0.3 | 0.874 (0.874) | 0.017 [0.140] | -0.27 \| 0.3 | 0.908 (0.908) |
| **Putamen** | | | | | | | | |
| Eloc | 0.52 ± 0.106 | 0.534 ± 0.103 | 0.014 [0.010] | -0.01 \| 0.04 | 0.360 (0.288) | 0.015 [0.010] | -0.01 \| 0.04 | 0.425 (0.255) |
| BC | 8.751 ± 4.079 | 7.768 ± 3.904 | -0.983 [0.500] | -1.96 \| 0 | 0.250 (0.050) | -0.832 [0.490] | -1.8 \| 0.14 | 0.465 (0.093) |
| **Pallidum** | | | | | | | | |
| Eloc | 0.336 ± 0.079 | 0.35 ± 0.08 | 0.014 [0.010] | -0.01 \| 0.03 | 0.293 (0.176) | 0.009 [0.010] | -0.01 \| 0.03 | 0.449 (0.359) |
| BC | 0.506 ± 1.278 | 0.393 ± 0.908 | -0.113 [0.140] | -0.38 \| 0.16 | 0.578 (0.407) | -0.128 [0.140] | -0.4 \| 0.15 | 0.600 (0.360) |
| **Hippocampus** | | | | | | | | |
| Eloc | 0.332 ± 0.068 | 0.345 ± 0.071 | 0.013 [0.010] | 0 \| 0.03 | 0.293 (0.136) | 0.012 [0.010] | -0.01 \| 0.03 | 0.425 (0.189) |
| BC | 9.03 ± 2.792 | 9.52 ± 4.038 | 0.491 [0.440] | -0.38 \| 1.36 | 0.578 (0.270) | 0.469 [0.460] | -0.43 \| 1.37 | 0.600 (0.305) |
| Reported connectome measures are adjusted for site using Combat. Significant differences are marked in bold. *Corrected for age, sex and education in years; Abbreviations: Q = modularity, RC = Rich Club, Eloc = local efficiency, BC = betweenness centrality. | | | | | | | | |

| **Supplementary Table 24 – SSRI/SNRI history analyses of global and nodal connectome measures – ComBat corrected** | | | | | | | | |
| --- | --- | --- | --- | --- | --- | --- | --- | --- |
|  | **NAIVE** | **NOT NAIVE** | **Group difference (crude model)#** | | | **Group difference (adjusted model)*** | | |
|  | M ± SD | M ± SD | B [SE] | 95% CI | P | B [SE] | 95% CI | P |
| **Global measures** | | | | | | | | |
| Small-worldness | 1.821 ± 0.16 | 1.832 ± 0.169 | -1.100 [2.100] | -5.1 \| 3 | 0.601 | -1.500 [2.000] | -5.5 \| 2.5 | 0.462 |
| Q | 0.578 ± 0.013 | 0.579 ± 0.013 | -0.100 [0.200] | -0.4 \| 0.2 | 0.536 | -0.200 [0.200] | -0.5 \| 0.1 | 0.253 |
| Global efficiency  (x 10^-2)^ | 2.9 ± 0.5 | 3.0 ± 0.5 | -10.700 [6.600] | -23.7 \| 2.3 | 0.107 | -11.300 [6.700] | -24.4 \| 1.9 | 0.093 |
| RC coefficient | 1.127 ± 0.231 | 1.127 ± 0.216 | <0.001 [2.800] | -5.5 \| 5.6 | 0.992 | -0.600 [2.800] | -6.2 \| 5 | 0.836 |
| **Nodal measures** | | | | | | | | |
| **Thalamus** | | | | | | | | |
| Eloc | 0.623 ± 0.117 | 0.643 ± 0.124 | -0.020 [0.010] | -0.05 \| 0.01 | 0.244 (0.195) | -0.019 [0.010] | -0.05 \| 0.01 | 0.255 (0.204) |
| BC | 36.992 ± 6.849 | 36.168 ± 7.101 | 0.824 [0.870] | -0.89 \| 2.54 | 0.562 (0.346) | 0.537 [0.860] | -1.16 \| 2.23 | 0.579 (0.533) |
| **Amygdala** | | | | | | | | |
| Eloc | 0.202 ± 0.066 | 0.207 ± 0.059 | -0.004 [0.010] | -0.02 \| 0.01 | 0.607 (0.607) | -0.004 [0.010] | -0.02 \| 0.01 | 0.634 (0.634) |
| BC | 1.657 ± 1.178 | 1.519 ± 1.086 | 0.138 [0.140] | -0.14 \| 0.42 | 0.562 (0.336) | 0.154 [0.140] | -0.13 \| 0.44 | 0.579 (0.283) |
| **Putamen** | | | | | | | | |
| Eloc | 0.518 ± 0.107 | 0.541 ± 0.101 | -0.022 [0.010] | -0.05 \| 0 | 0.145 (0.087) | -0.021 [0.010] | -0.05 \| 0 | 0.192 (0.115) |
| BC | 7.998 ± 3.787 | 8.454 ± 4.271 | -0.457 [0.500] | -1.44 \| 0.53 | 0.562 (0.363) | -0.272 [0.490] | -1.23 \| 0.69 | 0.579 (0.579) |
| **Pallidum** | | | | | | | | |
| Eloc | 0.336 ± 0.078 | 0.354 ± 0.081 | -0.017 [0.010] | -0.04 \| 0 | 0.145 (0.083) | -0.019 [0.010] | -0.04 \| 0 | 0.145 (0.058) |
| BC | 0.483 ± 1.24 | 0.387 ± 0.831 | 0.096 [0.140] | -0.17 \| 0.36 | 0.562 (0.479) | 0.096 [0.140] | -0.17 \| 0.37 | 0.579 (0.486) |
| **Hippocampus** | | | | | | | | |
| Eloc | 0.332 ± 0.069 | 0.349 ± 0.07 | -0.017 [0.010] | -0.03 \| 0 | 0.145 (0.048) | -0.018 [0.010] | -0.04 \| 0 | 0.145 (0.042) |
| BC | 9.191 ± 3.049 | 9.449 ± 4.117 | -0.258 [0.440] | -1.13 \| 0.62 | 0.562 (0.562) | -0.271 [0.450] | -1.16 \| 0.61 | 0.579 (0.547) |
| Significant differences are marked in bold. *Corrected for age, sex and education in years; Abbreviations: Q = modularity, RC = rich club, Eloc = local efficiency, BC = betweenness centrality | | | | | | | | |

| **Supplementary Table 25 – mixed model analyses of connectome measures and YBOCS – ComBat corrected** | | | | | | |
| --- | --- | --- | --- | --- | --- | --- |
|  | **crude model** | | | **adjusted model*** | | |
|  | B [SE] | 95% CI | P (unc) | B [SE] | 95% CI | P (unc) |
| **Global measures** | | | | | | |
| Small-worldness | <0.001 [0.200] | -0.4 \| 0.4 | 0.908 | 0.100 [0.200] | -0.3 \| 0.5 | 0.700 |
| Q | <0.001 [<0.001] | <0.001 \| <0.001 | 0.855 | <0.001 [<0.001] | <0.001 \| <0.001 | 0.969 |
| Global efficiency  (x 10^-2)^ | -0.700 [0.700] | -2 \| 0.6 | 0.316 | -0.700 [0.700] | -2 \| 0.6 | 0.299 |
| RC coefficient | <0.001 [0.300] | -0.6 \| 0.5 | 0.944 | <0.001 [0.300] | -0.6 \| 0.5 | 0.888 |
| **Nodal measures (x 10^-2^)** | | | | | | |
| **Thalamus** | | | | | | |
| Eloc | -0.001 [<0.001] | <0.001 \| <0.001 | 0.660 (0.396) | -0.001 [<0.001] | <0.001 \| <0.001 | 0.710 (0.449) |
| BC | 0.088 [0.090] | -0.09 \| 0.26 | 0.527 (0.316) | 0.064 [0.090] | -0.11 \| 0.24 | 0.736 (0.464) |
| **Amygdala** | | | | | | |
| Eloc | 0.001 [<0.001] | <0.001 \| <0.001 | 0.452 (0.181) | 0.001 [<0.001] | <0.001 \| <0.001 | 0.508 (0.203) |
| BC | -0.022 [0.010] | -0.05 \| 0.01 | 0.527 (0.119) | -0.018 [0.010] | -0.05 \| 0.01 | 0.590 (0.207) |
| **Putamen** | | | | | | |
| Eloc | -0.003 [<0.001] | <0.001 \| <0.001 | 0.210 (0.042) | -0.002 [<0.001] | <0.001 \| <0.001 | 0.380 (0.076) |
| BC | -0.033 [0.050] | -0.13 \| 0.07 | 0.644 (0.515) | -0.008 [0.050] | -0.11 \| 0.09 | 0.865 (0.865) |
| **Pallidum** | | | | | | |
| Eloc | <0.001 [<0.001] | <0.001 \| <0.001 | 0.836 (0.836) | <0.001 [<0.001] | <0.001 \| <0.001 | 0.904 (0.904) |
| BC | 0.006 [0.010] | -0.02 \| 0.03 | 0.667 (0.667) | 0.008 [0.010] | -0.02 \| 0.04 | 0.736 (0.589) |
| **Hippocampus** | | | | | | |
| Eloc | -0.001 [<0.001] | <0.001 \| <0.001 | 0.673 (0.538) | -0.001 [<0.001] | <0.001 \| <0.001 | 0.710 (0.568) |
| BC | 0.048 [0.040] | -0.04 \| 0.14 | 0.527 (0.283) | 0.054 [0.050] | -0.04 \| 0.14 | 0.590 (0.236) |
| Significant differences are marked in bold. *Corrected for age, sex and education in years; Abbreviations: Eloc = local efficiency, BC = betweenness centrality | | | | | | |

| **Supplementary Table 26 – Mixed model analyses of connectome measures and duration – ComBat corrected** | | | | | | |
| --- | --- | --- | --- | --- | --- | --- |
|  | **crude model** | | | **adjusted model*** | | |
|  | B [SE] | 95% CI | P (unc) | B [SE] | 95% CI | P (unc) |
| **Global measures** | | | | | | |
| Small-worldness | -0.100 [0.100] | -0.3 \| 0.1 | 0.227 | <0.001 [0.100] | -0.2 \| 0.2 | 0.667 |
| Q | <0.001 [<0.001] | <0.001 \| <0.001 | **0.009** | <0.001 [<0.001] | <0.001 \| <0.001 | 0.682 |
| Global efficiency  (x 10^-2)^ | 0.300 [0.300] | -0.3 \| 0.9 | 0.272 | 0.600 [0.300] | -0.1 \| 1.3 | 0.080 |
| RC coefficient | -0.100 [0.100] | -0.4 \| 0.1 | 0.288 | <0.001 [0.100] | -0.3 \| 0.2 | 0.789 |
| **Nodal measures (x 10^-2^)** | | | | | | |
| **Thalamus** | | | | | | |
| Eloc | 0.001 [<0.001] | <0.001 \| <0.001 | 0.208 (0.083) | 0.002 [<0.001] | <0.001 \| <0.001 | 0.170 (0.044) |
| BC | -0.071 [0.040] | -0.15 \| 0.01 | 0.280 (0.072) | 0.001 [0.040] | -0.09 \| 0.09 | 0.990 (0.990) |
| **Amygdala** | | | | | | |
| Eloc | <0.001 [<0.001] | <0.001 \| <0.001 | 0.732 (0.591) | <0.001 [<0.001] | <0.001 \| <0.001 | 0.507 (0.507) |
| BC | 0.007 [0.010] | <0.001 \| 0.02 | 0.442 (0.265) | 0.006 [0.010] | -0.01 \| 0.02 | 0.797 (0.411) |
| **Putamen** | | | | | | |
| Eloc | 0.001 [<0.001] | <0.001 \| <0.001 | 0.145 (0.029) | 0.001 [<0.001] | <0.001 \| <0.001 | 0.170 (0.068) |
| BC | 0.036 [0.020] | -0.01 \| 0.08 | 0.280 (0.112) | -0.003 [0.030] | -0.05 \| 0.05 | 0.990 (0.893) |
| **Pallidum** | | | | | | |
| Eloc | <0.001 [<0.001] | <0.001 \| <0.001 | 0.732 (0.732) | 0.001 [<0.001] | <0.001 \| <0.001 | 0.322 (0.258) |
| BC | -0.004 [0.010] | -0.02 \| 0.01 | 0.561 (0.525) | -0.005 [0.010] | -0.02 \| 0.01 | 0.797 (0.478) |
| **Hippocampus** | | | | | | |
| Eloc | <0.001 [<0.001] | <0.001 \| <0.001 | 0.555 (0.333) | 0.001 [<0.001] | <0.001 \| <0.001 | 0.197 (0.118) |
| BC | 0.012 [0.020] | -0.03 \| 0.05 | 0.561 (0.561) | 0.018 [0.020] | -0.03 \| 0.06 | 0.797 (0.445) |
| Significant differences are marked in bold. *Corrected for age, sex and education in years; Abbreviations: Eloc = local efficiency, BC = betweenness centrality | | | | | | |

| **Supplementary Table 27 – Mixed model analyses of connectome measures and age of onset as continuous measure – ComBat corrected** | | | | | | |
| --- | --- | --- | --- | --- | --- | --- |
|  | **crude model** | | | **adjusted model*** | | |
|  | B [SE] | 95% CI | P (unc) | B [SE] | 95% CI | P (unc) |
| **Global measures** | | | | | | |
| Small-worldness | -0.100 [0.100] | -0.3 \| 0.1 | 0.293 | -0.100 [0.100] | -0.30 \| 0.10 | 0.460 |
| Q | <0.001 [<0.001] | <0.001 \| <0.001 | 0.088 | <0.001 [<0.001] | <0.001 \| <0.001 | 0.444 |
| Global efficiency  (x 10^-2)^ | -0.300 [0.300] | -0.9 \| 0.3 | 0.355 | -0.300 [0.300] | -0.90 \| 0.40 | 0.420 |
| RC coefficient | -0.300 [0.100] | -0.5 \| 0 | 0.064 | -0.200 [0.100] | -0.50 \| 0.10 | 0.161 |
| **Nodal measures (x 10^-2^)** | | | | | | |
| **Thalamus** | | | | | | |
| Eloc | -0.001 [<0.001] | <0.001 \| <0.001 | 0.413 (0.165) | -0.001 [<0.001] | <0.001 \| <0.001 | 0.325 (0.127) |
| BC | -0.043 [0.040] | -0.13 \| 0.04 | 0.503 (0.316) | -0.008 [0.040] | -0.09 \| 0.08 | 0.848 (0.848) |
| **Amygdala** | | | | | | |
| Eloc | <0.001 [<0.001] | <0.001 \| <0.001 | 0.948 (0.813) | <0.001 [<0.001] | <0.001 \| <0.001 | 0.867 (0.867) |
| BC | 0.008 [0.010] | -0.01 \| 0.02 | 0.503 (0.278) | 0.006 [0.010] | -0.01 \| 0.02 | 0.784 (0.400) |
| **Putamen** | | | | | | |
| Eloc | <0.001 [<0.001] | <0.001 \| <0.001 | 0.948 (0.948) | <0.001 [<0.001] | <0.001 \| <0.001 | 0.867 (0.724) |
| BC | 0.039 [0.030] | -0.01 \| 0.09 | 0.503 (0.113) | 0.017 [0.030] | -0.03 \| 0.07 | 0.784 (0.490) |
| **Pallidum** | | | | | | |
| Eloc | <0.001 [<0.001] | <0.001 \| <0.001 | 0.550 (0.330) | <0.001 [<0.001] | <0.001 \| <0.001 | 0.757 (0.454) |
| BC | 0.006 [0.010] | -0.01 \| 0.02 | 0.503 (0.402) | 0.006 [0.010] | -0.01 \| 0.02 | 0.784 (0.382) |
| **Hippocampus** | | | | | | |
| Eloc | -0.001 [<0.001] | <0.001 \| <0.001 | 0.413 (0.118) | -0.001 [<0.001] | <0.001 \| <0.001 | 0.325 (0.130) |
| BC | -0.011 [0.020] | -0.05 \| 0.03 | 0.613 (0.613) | -0.011 [0.020] | -0.06 \| 0.03 | 0.784 (0.627) |
| Significant differences are marked in bold. *Corrected for age, sex and education in years; Abbreviations: Eloc = local efficiency, BC = betweenness centrality | | | | | | |

| **Supplementary Table 28 – Case-control mixed model analyses of connectome measures - random effects for site** | | | | | | |
| --- | --- | --- | --- | --- | --- | --- |
|  | **crude model** | | | **adjusted model*** | | |
|  | B [SE] | 95% CI | P (unc) | B [SE] | 95% CI | P (unc) |
| **Global measures** | | | | | | |
| Small-worldness | -1.500 [1.500] | -4.4 \| 1.4 | 0.302 | -1.900 [1.500] | -4.8 \| 1 | 0.208 |
| Q | -0.200 [0.100] | -0.4 \| 0.0 | 0.116 | -0.200 [0.100] | -0.5 \| 0.0 | 0.055 |
| Global efficiency  (x 10^-2)^ | 0.900 [4.500] | -7.9 \| 9.8 | 0.836 | 0.100 [4.600] | -8.9 \| 9.1 | 0.986 |
| RC coefficient | -1.000 [1.900] | -4.7 \| 2.8 | 0.613 | -0.800 [1.900] | -4.5 \| 3.0 | 0.692 |
| **Nodal measures (x 10^-2^)** | | | | | | |
| **Thalamus** | | | | | | |
| Eloc | 0.013 [0.010] | -0.01 \| 0.03 | 0.892 (0.210) | 0.011 [0.010] | -0.01 \| 0.03 | 0.955 (0.291) |
| BC | -0.165 [0.620] | -1.39 \| 1.06 | 0.790 (0.790) | -0.326 [0.610] | -1.53 \| 0.88 | 0.742 (0.594) |
| **Amygdala** | | | | | | |
| Eloc | -0.002 [0.010] | -0.01 \| 0.01 | 0.892 (0.691) | -0.002 [0.010] | -0.01 \| 0.01 | 0.955 (0.686) |
| BC | -0.145 [0.100] | -0.34 \| 0.06 | 0.700 (0.154) | -0.135 [0.100] | -0.34 \| 0.06 | 0.490 (0.186) |
| **Putamen** | | | | | | |
| Eloc | 0.001 [0.010] | -0.02 \| 0.02 | 0.892 (0.887) | -0.001 [0.010] | -0.02 \| 0.02 | 0.955 (0.955) |
| BC | -0.368 [0.350] | -1.05 \| 0.32 | 0.700 (0.293) | -0.453 [0.350] | -1.14 \| 0.23 | 0.490 (0.196) |
| **Pallidum** | | | | | | |
| Eloc | 0.002 [0.010] | -0.01 \| 0.01 | 0.892 (0.773) | 0.001 [0.010] | -0.01 \| 0.01 | 0.955 (0.904) |
| BC | 0.080 [0.100] | -0.12 \| 0.28 | 0.700 (0.420) | 0.076 [0.100] | -0.12 \| 0.27 | 0.742 (0.449) |
| **Hippocampus** | | | | | | |
| Eloc | 0.001 [0.010] | -0.01 \| 0.01 | 0.892 (0.892) | -0.001 [0.010] | -0.01 \| 0.01 | 0.955 (0.904) |
| BC | -0.082 [0.300] | -0.67 \| 0.51 | 0.790 (0.785) | -0.080 [0.310] | -0.68 \| 0.52 | 0.794 (0.794) |
| Significant differences are marked in bold. *Corrected for age, sex and education in years; Abbreviations: Eloc = local efficiency, BC = betweenness centrality | | | | | | |

| **Supplementary Table 29 – Age of OCD onset mixed model analyses of connectome measures - random effects for site** | | | | | | |
| --- | --- | --- | --- | --- | --- | --- |
|  | **crude model** | | | **adjusted model*** | | |
|  | B [SE] | 95% CI | P (unc) | B [SE] | 95% CI | P (unc) |
| **Global measures** | | | | | | |
| Small-worldness | 0.600 [2.200] | -3.7 \| 4.9 | 0.799 | -1.100 [2.200] | -5.5 \| 3.2 | 0.612 |
| Q | 0.100 [0.200] | -0.2 \| 0.5 | 0.395 | -0.100 [0.200] | -0.4 \| 0.3 | 0.700 |
| Global efficiency  (x 10^-2)^ | 16.500 [6.800] | 3.1 \| 29.9 | **0.016** | 16.400 [7.100] | 2.5 \| 30.3 | **0.021** |
| RC coefficient | 2.500 [2.900] | -3.2 \| 8.2 | 0.395 | 1.600 [3.000] | -4.3 \| 7.5 | 0.595 |
| **Nodal measures (x 10^-2^)** | | | | | | |
| **Thalamus** | | | | | | |
| Eloc | 0.039 [0.010] | 0.01 \| 0.07 | 0.060 (0.012) | 0.040 [0.020] | 0.01 \| 0.07 | 0.060 (0.012) |
| BC | 0.919 [0.900] | -0.85 \| 2.69 | 0.513 (0.308) | 0.455 [0.910] | -1.34 \| 2.25 | 0.772 (0.618) |
| **Amygdala** | | | | | | |
| Eloc | <0.001 [0.010] | -0.02 \| 0.02 | 0.976 (0.976) | 0.002 [0.010] | -0.01 \| 0.02 | 0.833 (0.833) |
| BC | 0.017 [0.140] | -0.27 \| 0.3 | 0.904 (0.904) | -0.001 [0.150] | -0.29 \| 0.29 | 0.995 (0.995) |
| **Putamen** | | | | | | |
| Eloc | 0.017 [0.010] | -0.01 \| 0.04 | 0.255 (0.204) | 0.019 [0.010] | -0.01 \| 0.05 | 0.298 (0.179) |
| BC | -1.101 [0.520] | -2.13 \| -0.07 | 0.180 (0.036) | -1.012 [0.530] | -2.05 \| 0.03 | 0.285 (0.057) |
| **Pallidum** | | | | | | |
| Eloc | 0.016 [0.010] | <0.001 \| 0.04 | 0.190 (0.114) | 0.012 [0.010] | -0.01 \| 0.03 | 0.315 (0.252) |
| BC | -0.074 [0.170] | -0.41 \| 0.27 | 0.838 (0.670) | -0.131 [0.180] | -0.48 \| 0.22 | 0.768 (0.461) |
| **Hippocampus** | | | | | | |
| Eloc | 0.015 [0.010] | <0.001 \| 0.03 | 0.190 (0.110) | 0.014 [0.010] | <0.001 \| 0.03 | 0.298 (0.148) |
| BC | 0.573 [0.460] | -0.34 \| 1.49 | 0.513 (0.217) | 0.530 [0.480] | -0.42 \| 1.48 | 0.677 (0.271) |
| Significant differences are marked in bold. *Corrected for age, sex and education in years; Abbreviations: Eloc = local efficiency, BC = betweenness centrality | | | | | | |

| **Supplementary Table 30 – SSRI/SNRI history mixed model analyses of connectome measures - random effects for site** | | | | | | |
| --- | --- | --- | --- | --- | --- | --- |
|  | **crude model** | | | **adjusted model*** | | |
|  | B [SE] | 95% CI | P (unc) | B [SE] | 95% CI | P (unc) |
| **Global measures** | | | | | | |
| Small-worldness | -0.400 [2.200] | -4.7 \| 3.9 | 0.856 | -1.000 [2.100] | -5.2 \| 3.3 | 0.654 |
| Q | -0.100 [0.200] | -0.4 \| 0.2 | 0.607 | -0.200 [0.200] | -0.5 \| 0.1 | 0.296 |
| Global efficiency  (x 10^-2)^ | -12.000 [6.800] | -25.4 \| 1.4 | 0.079 | -12.600 [6.900] | -26.1 \| 1.0 | 0.069 |
| RC coefficient | -0.400 [2.900] | -6.1 \| 5.3 | 0.879 | -0.900 [2.900] | -6.7 \| 4.8 | 0.749 |
| **Nodal measures (x 10^-2^)** | | | | | | |
| **Thalamus** | | | | | | |
| Eloc | -0.023 [0.010] | -0.05 \| 0.01 | 0.166 (0.133) | -0.024 [0.020] | -0.05 \| 0.01 | 0.162 (0.130) |
| BC | 0.839 [0.900] | -0.92 \| 2.6 | 0.593 (0.350) | 0.607 [0.880] | -1.13 \| 2.35 | 0.615 (0.492) |
| **Amygdala** | | | | | | |
| Eloc | -0.005 [0.010] | -0.02 \| 0.01 | 0.580 (0.580) | -0.004 [0.010] | -0.02 \| 0.01 | 0.632 (0.632) |
| BC | 0.130 [0.140] | -0.15 \| 0.41 | 0.593 (0.369) | 0.131 [0.140] | -0.15 \| 0.42 | 0.615 (0.362) |
| **Putamen** | | | | | | |
| Eloc | -0.025 [0.010] | -0.05 \| <0.001 | 0.133 (0.067) | -0.024 [0.010] | -0.05 \| <0.001 | 0.123 (0.074) |
| BC | -0.281 [0.520] | -1.31 \| 0.75 | 0.593 (0.593) | -0.179 [0.520] | -1.2 \| 0.84 | 0.729 (0.729) |
| **Pallidum** | | | | | | |
| Eloc | -0.018 [0.010] | -0.04 \| <0.001 | 0.133 (0.080) | -0.020 [0.010] | -0.04 \| <0.001 | 0.123 (0.053) |
| BC | 0.213 [0.170] | -0.12 \| 0.55 | 0.593 (0.214) | 0.193 [0.170] | -0.15 \| 0.53 | 0.615 (0.263) |
| **Hippocampus** | | | | | | |
| Eloc | -0.018 [0.010] | -0.04 \| <0.001 | 0.133 (0.050) | -0.019 [0.010] | -0.04 \| <0.001 | 0.123 (0.042) |
| BC | -0.302 [0.460] | -1.21 \| 0.61 | 0.593 (0.514) | -0.330 [0.470] | -1.25 \| 0.59 | 0.615 (0.480) |
| Significant differences are marked in bold. *Corrected for age, sex and education in years; Abbreviations: Eloc = local efficiency, BC = betweenness centrality | | | | | | |

| Supplementary Table 31 – all clinical measures assessed in global OCD study | |
| --- | --- |
| Clinical Measures | |
| Total Severity | Yale-Brown Obsessive-Compulsive Scale (Y-BOCS)  Obsessive-Compulsive Inventory-R |
| Dimension Severity | Dimensional Yale-Brown Obsessive-Compulsive Scale (DY-BOCS) |
| Insight | Brown Assessment of Beliefs Scale (BABS) |
| Sensory Phenomena | University of São Paulo Sensory Phenomena Scale (USP-SPS) |
| Age of Onset | Structured Clinical Interview for DSM-5 (SCID); Center for OCD and Related Disorders Age of Onset and Course Form |
| Depression | Hamilton Depression Rating Scale (HAM-D) |
| Anxiety | Hamilton Anxiety Rating Scale (HAM-A) |
| Other Clinical measures | Autism Spectrum Quotient  Center for OCD and Related Disorders Tic Questionnaire  Disgust Propensity and Sensitivity Scale  Impulsive-Compulsive Behaviours Checklist  Obsessive-Compulsive Personality Disorder Questionnaire |
| Functioning | World Health Organization Disability Assessment Schedule 2.0 (WHODAS) |
| Socioeconomic Status | Work and Meaning Inventory (WAMI) |
| Trauma | Childhood Trauma Questionnaire |
| Religiosity | Religious Behaviors and Beliefs Questionnaire |

| **Supplementary Table 32 –Demographic and clinical characteristics of sample used for template creation for the fixel-based analysis** | | | |
| --- | --- | --- | --- |
|  | **N=125 template sample** | | |
|  | | **OCD (N=62)** | **HC (n=63)** |
| **Sex (N (%))** | |  |  |
| **Male** | | 29 (46.8%) | 25 (39.7%) |
| **Female** | | 33 (53.2%) | 38 (60.3%) |
| **Age (years)** | | 30.2 (8.7) | 30.0 (8.7) |
| **Education (years)** | | 14.8 (2.3) | 16.1 (1.9) |
| **Estimated IQ** | | 104.2 (14.4) | 107.9 (10.9) |
| **YBOCS** | | 25.0 (4.8) | 0.3 (1.2) |
| **Duration of illness^a^** | | 13.0 (8.9) |  |
| **Age of onset (years)** | | 17.3 (7.7) | - |
| **OCD onset (N (%))^a^** | |  |  |
| Child onset | | 39 (62.9%) | - |
| Adult onset | | 22 (35.5%) | - |
| Data are presented as mean (SD) unless otherwise indicated. a = duration of illness/age of onset missing for 1 patient. Abbreviations: YBOCS = Yale-Brown Obsessive-compulsive Scale, | | | |

**REFERENCES**

Alstott, Jeff, Pietro Panzarasa, Mikail Rubinov, Edward T. Bullmore, and Petra E. Vértes. 2014. “A Unifying Framework for Measuring Weighted Rich Clubs.” *Scientific Reports* 4 (1): 7258. https://doi.org/10.1038/srep07258.

Baldi, Samantha, Stijn Michielse, Chris Vriend, Martijn P van den Heuvel, Odile A van den Heuvel, Koen RJ Schruers, and Liesbet Goossens. 2022. “Abnormal White‐matter Rich‐club Organization in Obsessive–Compulsive Disorder.” *Human Brain Mapping* 43 (15): 4699–4709.

Bastiani, M., M. Cottaar, S. P. Fitzgibbon, S. Suri, F. Alfaro-Almagro, S. N. Sotiropoulos, S. Jbabdi, and J. L. R. Andersson. 2019. “Automated Quality Control for within and between Studies Diffusion MRI Data Using a Non-Parametric Framework for Movement and Distortion Correction.” *Neuroimage* 184 (January): 801–12. https://doi.org/10.1016/j.neuroimage.2018.09.073.

Chen, Gang, Yaqiong Xiao, Paul A Taylor, Justin K Rajendra, Tracy Riggins, Fengji Geng, Elizabeth Redcay, and Robert W Cox. 2019. “Handling Multiplicity in Neuroimaging through Bayesian Lenses with Multilevel Modeling.” *Neuroinformatics* 17: 515–45.

Jeurissen, B., A. Leemans, J. D. Tournier, D. K. Jones, and J. Sijbers. 2013. “Investigating the Prevalence of Complex Fiber Configurations in White Matter Tissue with Diffusion Magnetic Resonance Imaging.” *Hum Brain Mapp* 34 (11): 2747–66. https://doi.org/10.1002/hbm.22099.

Pouwels, P. J. W., C. Vriend, F. Liu, N. T. de Joode, M. C. G. Otaduy, B. Pastorello, F. C. Robertson, et al. 2023. “Global Multi-Center and Multi-Modal Magnetic Resonance Imaging Study of Obsessive-Compulsive Disorder: Harmonization and Monitoring of Protocols in Healthy Volunteers and Phantoms.” *Int J Methods Psychiatr Res* 32 (1): e1931. https://doi.org/10.1002/mpr.1931.

Raffelt, D. A., J. D. Tournier, R. E. Smith, D. N. Vaughan, G. Jackson, G. R. Ridgway, and A. Connelly. 2017. “Investigating White Matter Fibre Density and Morphology Using Fixel-Based Analysis.” *Neuroimage* 144 (Pt A): 58–73. https://doi.org/10.1016/j.neuroimage.2016.09.029.

Tournier, J. D., R. Smith, D. Raffelt, R. Tabbara, T. Dhollander, M. Pietsch, D. Christiaens, B. Jeurissen, C. H. Yeh, and A. Connelly. 2019. “MRtrix3: A Fast, Flexible and Open Software Framework for Medical Image Processing and Visualisation.” *Neuroimage* 202 (November): 116137. https://doi.org/10.1016/j.neuroimage.2019.116137.
